# Photophysical characterization and fluorescence cell imaging applications of 4-*N*-substituted benzothiadiazoles†

**DOI:** 10.1039/d2ra01404a

**Published:** 2022-05-13

**Authors:** Susanne Doloczki, Karl O. Holmberg, Ignacio Fdez. Galván, Fredrik J. Swartling, Christine Dyrager

**Affiliations:** Department of Chemistry – BMC, Uppsala University Box 576 75123 Uppsala Sweden christine.dyrager@kemi.uu.se; Department of Immunology, Genetics and Pathology, Rudbeck Laboratory, Uppsala University 75185 Uppsala Sweden

## Abstract

In this work, a series of fluorescent 2,1,3-benzothiadiazole derivatives with various *N*-substituents in the 4-position was synthesized and photophysically characterized in various solvents. Three compounds emerged as excellent fluorescent probes for imaging lipid droplets in cancer cells. A correlation between their high lipophilicity and lipid droplet specificity could be found, with log *P* ≥ 4 being characteristic for lipid droplet accumulation.

## Abstract 2

Herein we report the structure–photophysical relationship and cell imaging utility of a series of *N*-substituted benzothiadiazoles.

## Graphical abstract

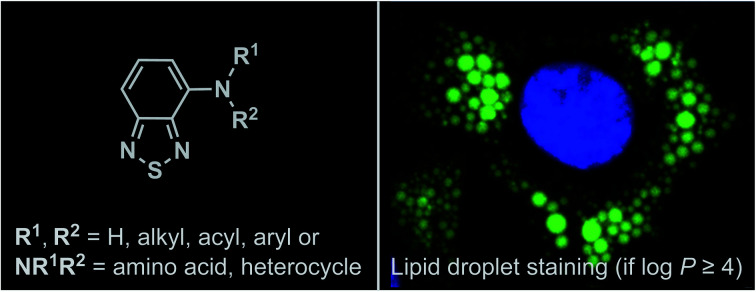

## Introduction

**Fig. 1:**
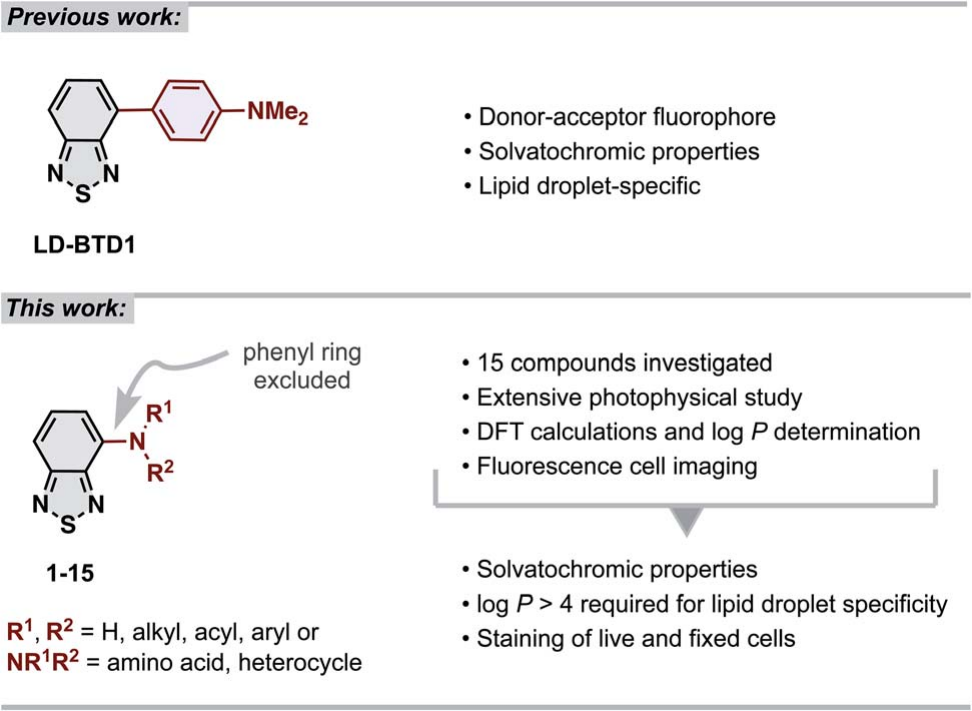
(Top) previous work: our previously reported lipid droplet dye, LD-BTD1.^11^ (Bottom) this work: extensive photophysical study of 4-*N*-substituted BTD derivatives and their utility as imaging agents for fluorescence cell microscopy.

The 2,1,3-benzothiadiazole (BTD) unit is a key component of numerous highly tuneable fluorophores. BTD is electron-deficient, and its derivatives generally exhibit high photostability, large Stokes shifts and solvatochromic properties.^1–4^ These advantages have been broadly recognized in materials science for use as building blocks in conjugated polymers (*e.g.*, in solar cell components).^5–10^ Recently, the BTD-motif has also shown its potential as a bioimaging scaffold for fluorescence cell microscopy.^2,3^ Our contribution to the latter has been the development of BTD-based dyes that specifically stain intracellular lipid droplets (LDs),^11–13^ lipid-rich organelles that are strongly associated with cancer progression and cancer cell survival.^14–17^ LDs serve as a biomarker for cancer diagnosis and prognosis and their relative quantity can be used as a measure for therapeutic response.^14–17^ Thus, LD-specific fluorophores are important molecular tools in the field of oncology. Several fluorescent probes for imaging LDs have been reported,^18,19^ including a few BTD derivatives described by other research groups.^3,20–23^ While many of these have shown advantages over the commonly used commercialized fluorescent probes (*e.g.*, Nile Red and BODIPY™ 493/503), they often feature drawbacks, including unspecific staining, small Stokes shifts or not being easily accessible.^18^ Our previously reported LD-specific dye, LD-BTD1 (Fig. 1), overcomes each of these drawbacks.^11^ LD-BTD1 is readily synthesized in one step and displays large Stokes shifts, strong solvatochromism, and high fluorescence quantum yields in apolar solvents, as well as specific and bright staining of lipid droplets in fixed or live cancer cells.

**Scheme 1:**
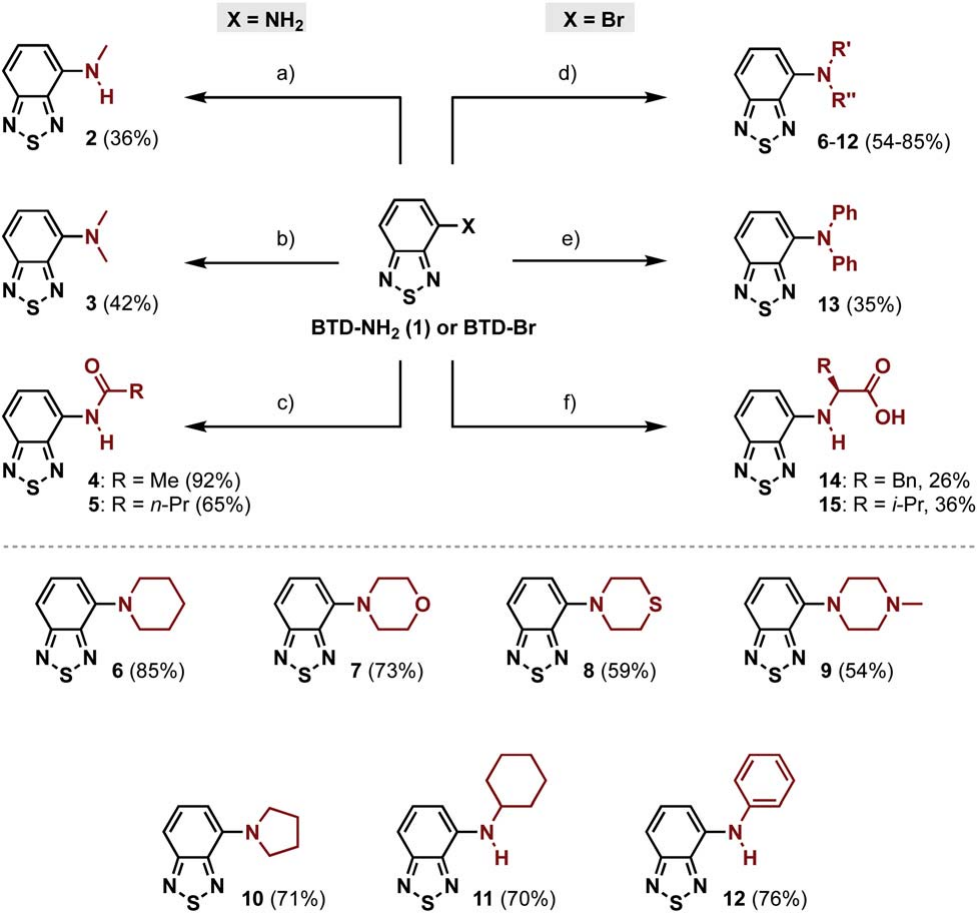
Synthesis of amino- and amido-BTD derivatives (2–15) starting from BTD-NH_2_ (2–5) or BTD-Br (6–15). (a) MeI, K_2_CO_3_, DMF, 50 °C, 20.5 h; (b) paraformaldehyde, NaBH_3_CN, AcOH, rt, 26 h; (c) acyl chloride, pyridine, CH_2_Cl_2_, 0 °C to rt, 2.5–3.5 h; (d) amine, PEPPSI-IPr, *t*-BuOK, toluene, 120 °C, 3.5–4.5 h; (e) HNPh_2_, Pd_2_(dba)_3_, RuPhos, Cs_2_CO_3_, toluene, 120 °C, 24 h; (f) l-amino acid, CuI, K_2_CO_3_, DMA, 90 °C, 5–6 d.

In this work we have explored the photophysical properties and cell imaging potential of 4-*N*-substituted BTDs – structures that lack the phenyl ring between the acceptor unit (BTD) and the amine donor, as present in LD-BTD1 (Fig. 1). We hypothesized that this approach could provide new solvatochromic fluorophores that retain selectivity for lipid droplets if they possess an appropriate hydrophobicity. Fourteen BTD derivatives with various *N*-substituents in the 4-position were synthesized and, together with 4-amino-BTD (BTD-NH_2_, 1, Scheme 1), subjected to an extensive photophysical study in solvents of different polarity. The octanol/water partition coefficient (log *P*) was calculated and, if practicable, experimentally determined. In addition, all compounds were investigated as potential fluorescent probes for cancer cell imaging (*e.g.*, for staining LDs).

## Results and discussion

### Synthesis

The 4-amino-BTDs (2, 3 and 6–15) were synthesized from BTD-NH_2_(1) or BTD-Br in one step, *e.g.*, *via* metal-catalyzed cross-coupling reactions, whereas the 4-amido-BTDs (4 and 5) were obtained through nucleophilic addition (Scheme 1).

### Photophysical characterization

The photophysical properties of compounds 1–15 were investigated using UV-vis absorption and fluorescence spectroscopy in solvents of different polarity (Table 1). Measurements in aqueous solution were performed in water with 5% DMSO to facilitate solubilization.

**Table 1:** Photophysical data of the 4-amino- and 4-amido-substituted BTD derivatives (1–15) in solvents of different polarity

Compd.	Solvent	*λ*_Amax_ a	*λ*_Emax_ a	Stokes shift^a^	*ε* b	*φ*_F_
1	Hexane	400	501	101 (5040)	2000	0.37
	Toluene	410	537	127 (5768)	2700	0.24
	THF	425	568	143 (5924)	2200	0.14
	DMSO	437	618	181 (6702)	3000	<0.01
	MeOH	421	628	207 (7829)	2700	<0.01
	H_2_O^c^	403	n.e.	—	2400	—

2	Hexane	420	513	93 (4316)	3500	0.42
	Toluene	430	553	123 (5173)	3200	0.34
	THF	437	568	131 (5278)	3400	0.22
	DMSO	446	615	169 (6161)	2800	0.03
	MeOH	437	629	192 (6985)	3300	<0.01
	H_2_O^c^	429	n.e.	—	2300	—

3	Hexane	424	523	99 (4464)	3600	0.41
	Toluene	432	558	126 (5227)	2800	0.34
	THF	434	576	142 (5680)	2700	0.24
	DMSO	440	624	184 (6702)	3200	0.06
	MeOH	432	625	193 (7148)	2800	<0.01
	H_2_O^c^	394	n.e.	—	2800	—

4	Hexane	372	436	64 (3946)	3100	0.50
			465	93 (5376)		
	Toluene	374	483	109 (6034)	3400	0.79
	THF	374	488	114 (6246)	3900	0.72
	DMSO	373	510	137 (7202)	4200	0.72
	MeOH	362	516	154 (8244)	3800	0.17
	H_2_O^c^	352	536	184 (9752)	3200	0.02

5	Hexane	373	439	66 (4031)	4800	0.58
			467	94 (5396)		
	Toluene	375	485	110 (6048)	3400	0.76
	THF	374	486	112 (6162)	4000	0.69
	DMSO	373	509	136 (7163)	4100	0.73
	MeOH	364	515	151 (8055)	3700	0.16
	H_2_O^c^	354	537	183 (9627)	3100	0.02

6	Hexane	413	533	120 (5451)	3700	0.48
	Toluene	420	569	149 (6235)	3000	0.38
	THF	420	586	166 (6745)	3100	0.28
	DMSO	423	627	204 (7692)	2500	0.07
	MeOH	413	635	222 (8465)	2700	<0.01
	H_2_O^c^	388	575^d^	187 (8382)	n.d.^e^	n.d.^d^

7	Hexane	403	530	127 (5946)	3300	0.59
	Toluene	409	563	154 (6688)	4000	0.50
	THF	408	578	170 (7209)	3800	0.32
	DMSO	412	620	208 (8143)	3000	0.08
	MeOH	402	629	227 (8977)	2900	<0.01
	H_2_O^c^	380	n.e.	—	2600	—

8	Hexane	405	526	121 (5680)	3100	0.63
	Toluene	414	557	143 (6201)	3000	0.60
	THF	412	573	161 (6820)	3200	0.36
	DMSO	417	614	197 (7694)	2800	0.13
	MeOH	408	618	210 (8329)	2700	<0.01
	H_2_O^c^	381	n.e.	—	n.d.^e^	—

9	Hexane	409	532	123 (5653)	3500	0.55
	Toluene	414	565	151 (6455)	2700	0.19
	THF	414	583	169 (7002)	4000	0.05
	DMSO	416	620	204 (7909)	3100	<0.01
	MeOH	402	624	222 (8850)	3100	<0.01
	H_2_O^c^	380	n.e.	—	2800	—

10	Hexane	446	526	80 (3410)	4900	0.53
	Toluene	458	563	105 (4072)	5000	0.44
	THF	457	575	118 (4491)	4200	0.28
	DMSO	464	620	156 (5423)	3100	0.08
	MeOH	459	633	174 (5989)	4300	<0.01
	H_2_O^c^	460	579^d^	119 (4468)	n.d.^e^	n.d.^d^

11	Hexane	431	523	92 (4081)	3500	0.52
	Toluene	438	554	116 (4781)	3500	0.34
	THF	442	570	128 (5081)	3400	0.23
	DMSO	451	619	168 (6018)	3200	0.03
	MeOH	443	629	186 (6675)	3100	<0.01
	H_2_O^c^	438	n.e.	—	n.d.^e^	—

12	Hexane	432	511	79 (3579)	6300	0.58
	Toluene	439	544	105 (4397)	5300	0.43
	THF	443	563	120 (4811)	6000	0.25
	DMSO	450	615	165 (5962)	5500	<0.01
	MeOH	441	n.e.	—	5400	—
	H_2_O^c^	441	562^d^	121 (4882)	n.d.^e^	n.d.^d^

13	Hexane	448	531	83 (3489)	4900	0.45
	Toluene	453	573	120 (4623)	4000	0.29
	THF	451	594	143 (5338)	3100	0.13
	DMSO	451	638	187 (6499)	3700	0.01
	MeOH	449	n.e.	—	3900	—
	H_2_O^c^	Insufficient solubility				

14	Hexane	409	507	98 (4726)	n.d.^e^	0.53
	Toluene	414	535	121 (5463)	2800	0.54
	THF	426	562	136 (5681)	2900	0.34
	DMSO	434	598	164 (6319)	3000	0.05
	MeOH	427	617	190 (7212)	2800	<0.01
	H_2_O^c^	430	n.e.	—	2800	—

15	Hexane	411	507	96 (4607)	2200	0.52
	Toluene	416	535	119 (5347)	2800	0.47
	THF	427	553	126 (5336)	2800	0.35
	DMSO	433	600	167 (6428)	2700	0.07
	MeOH	428	615	187 (7104)	3200	<0.01
	H_2_O^c^	434	n.e.	—	2200	—

a In nm and (cm^−1^).

b In M^−1^ cm^−1^.

c 5% DMSO in water.

d Emission from precipitating particles.

e Not determined due to poor solubility. n.e. = no emission detected. n.d. = not determined.

**Fig. 2:**
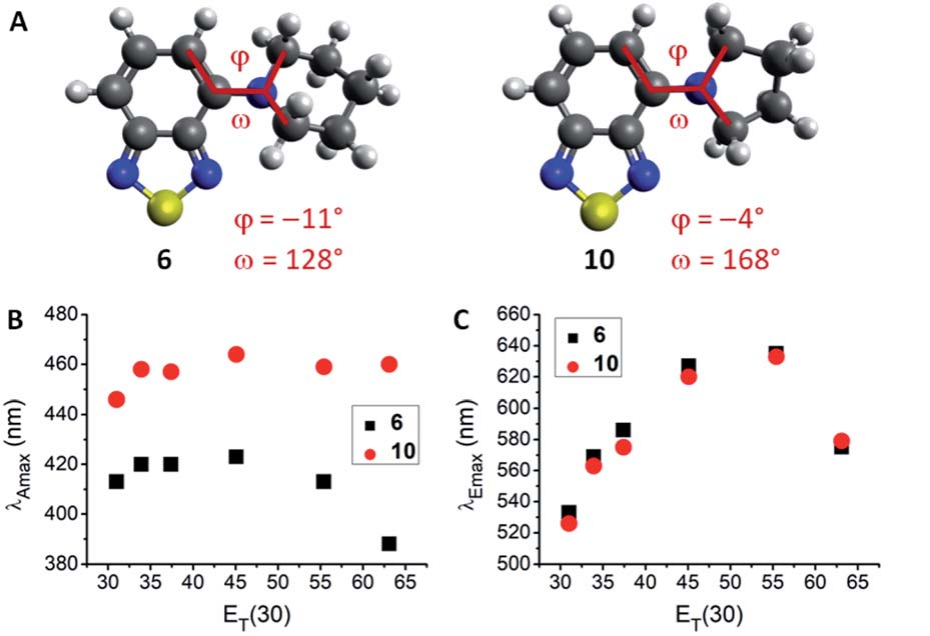
Comparison of 6 and 10. (A) Optimized ground state geometries in hexane. Dihedral angles (*φ* and *ω*) indicating the geometry around the nitrogen atoms are displayed, with 6 being more pyramidalized. (B) Absorption wavelength dependence on *E*_T_(30) solvent polarity parameter. (C) Emission wavelength dependence on *E*_T_(30) solvent polarity parameter. Emission in water could be ascribed to aggregates/precipitation.

The lowest energy absorption maximum (*λ*_Amax_) of most amines (1–3, 6–12, 14 and 15) displayed small bathochromic shifts (a few nm) with increasing solvent polarity (*i.e.*, positive solvatochromism). However, this trend did not generally apply in polar protic solvents (MeOH and H_2_O). For tertiary amines (3, 6–9) even a hypsochromic shift of about 25 nm in water compared to hexane was observed. Blue-shifted *λ*_Amax_ of aromatic amines in polar protic solvents are commonly ascribed to hydrogen bonding interactions between the nitrogen lone pair and solvent protons.^24–26^ Primary and secondary amines are capable of acting as hydrogen bond donors (in addition to their hydrogen bond acceptor ability) which leads to an inverse effect (*i.e.*, red-shift) on *λ*_Amax_. Therefore, primary and secondary amines 1, 2, 11, 12, 14, and 15 displayed red-shifted *λ*_Amax_, whereas tertiary amines 3 and 6–9 featured blue-shifted *λ*_Amax_ in water compared to hexane (for correlations with hydrogen bond energy see ESI, Fig. S27†). A few deviations from these trends were found among the studied compounds. For instance, the absorption wavelength of 13 was independent of solvent polarity, likely due to N lone pair conjugation with the three aromatic rings. The amides 4 and 5 were insensitive to aprotic solvents but exhibited hypsochromic shifts in polar protic solvents (*e.g.*, *ca.* 20 nm in water compared to hexane). Furthermore, pyrrolidino-BTD 10 did not behave like the other tertiary amines as it featured red-shifted absorption in water (14 nm compared to hexane). DFT calculations of 6 and 10 indicated that the nitrogen atom is less pyramidalized in the latter case (Fig. 2A). A flattened geometry around the nitrogen atom facilitates conjugation of the nitrogen lone pair with the BTD π-system and makes 10 less susceptible to hydrogen bonding with the solvent (calculated hydrogen bond energies are 21.4 kJ mol^−1^ for 6 and 12.0 kJ mol^−1^ for 10).^24^ Further consequences of this were red-shifted *λ*_Amax_ (Fig. 2B) as well as higher molar extinction coefficients (*ε*) and brightness (*ε* × *φ*_F_) of 10 in comparison with the larger cyclic amines (6–9).

Regarding the maximum emission wavelength (*λ*_Emax_), a clear trend was observed for all amino-BTDs, independent of their hydrogen bonding properties. For instance, compounds 6 and 10 showed the same behaviour in terms of *λ*_Emax_ (Fig. 2C). Furthermore, all studied compounds (1–15) showed positive solvatochromic fluorescence with a linear relationship between *λ*_Emax_ and Reichardt's *E*_T_(30) solvent polarity parameter^27^ in aprotic solvents (for amides also in protic solvents; ESI, Fig. S20†).

Emission wavelengths for the amines were in the range of 501–638 nm and blue-shifted to 436–537 nm for the amides. A linear correlation in aprotic solvents was also observed between fluorescence quantum yields (*φ*_F_) and *E*_T_(30) (ESI, Fig. S20†). Thus, the fluorescence quantum yields of the amino-BTDs (1–3 and 6–15) decreased steadily upon increasing the solvent polarity, with complete quenching in polar protic solvents. The detected fluorescence of 6, 10 and 12 in water could be ascribed to aggregation and visible precipitation of the compounds, which are fluorescent in the solid state. Their blue-shifted emission was also indicative of the presence of aggregates.^28^ The fluorescence quantum yields for the amido-BTDs (4 and 5) remained high in aprotic solvents (*φ*_F_ ≈ 0.70) while MeOH (*φ*_F_ = 0.17 and 0.16, respectively) and water (*φ*_F_ = 0.02) caused partial quenching. The reason for less pronounced quenching in comparison with the amines might be decreased hydrogen bonding ability due to electron delocalization. Another unique feature within the series of compounds was observed for 4 and 5, as these showed dual emission in hexane (ESI, Fig. S4 and S5†). This feature is in line with findings from our recent study on substituted aryl-BTDs, which showed dual emission arising from the locally excited (LE) and intramolecular charge transfer (ICT) excited states – the latter of which was promoted by strongly electron-donating substituents and polar solvents.^12^ This ICT effect has also been discussed for other BTD-based fluorophores.^29,30^ It therefore seems reasonable to suggest that single emission profiles from amines in this work arise from the ICT state, whereas the weakened donor ability of the amides causes competing LE/ICT emission in hexane that is pushed to sole ICT emission in more polar solvents.

A few other notable differences in photophysical properties for some of the compound classes could be observed. For instance, the six-membered cyclic amines (6–9) had comparable photophysical behaviour independent of the integrated heteroatom. However, 9 experienced considerably stronger fluorescence quenching with increasing solvent polarity. Compounds 6–9 featured very large Stokes shifts (120–227 nm), with 7 representing the largest value of all our studied BTD derivatives. Large Stokes shifts were seen throughout the whole series (1–15) – a characteristic that is favourable for bioimaging applications.

Many of the compounds showed low molar extinction coefficients (*ε*), typically <4000 M^−1^ cm^−1^. However, addition of one phenyl substituent on the nitrogen (12) increased *ε* almost two-fold in comparison with the corresponding aliphatic derivative 11 (*cf.* 6300 *vs.* 3500 M^−1^ cm^−1^ in hexane). Consequently, 12 displayed the highest brightness among the studied amines 1–3 and 6–15 (notably, the amides were brighter in most solvents). Addition of a second phenyl substituent (13) did not have an amplifying effect. On the contrary, it generated lower *ε* and *φ*_F_ values. Nevertheless, compound 13 featured the most red-shifted fluorescence (*e.g.*, 638 nm in DMSO) in the series of compounds.

Methylation of 1 to give 2 led to red-shifted absorption and emission (20 and 12 nm in hexane, respectively). The impact of a second methyl group (3), especially on *λ*_Amax_, was smaller than that of the first methyl group (only 4 nm red-shift in hexane). The *ε* and *φ*_F_ values for 2 and 3 were comparable and in general higher than for 1. It should be noted that compound 3 in comparison to its π-extended equivalent LD-BTD1 (ref. 11) (Fig. 1) featured similar absorption and emission wavelengths. The fluorescence quantum yields, however, were generally lower for 3.

Plots of Stokes shifts *vs.* the *E*_T_(30) solvent polarity parameter showed a better linear correlation (*R*^2^ ≥ 0.892) than ordinary Lippert–Mataga plots^31,32^ (*R*^2^ ≥ 0.608), which are based on the solvent orientation polarizability parameter (ESI, Fig. S16–S19†). This linearity indicated that the observed spectral shifts are mainly caused by general solvent effects such as polarity. The relatively large positive slopes of the Lippert–Mataga plots indicated a significant increase in dipole moment upon excitation, which is consistent with the expected increase in charge separation in the ICT excited state.

The smallest compounds in this series, 1–4, were chosen for computational studies using hexane, THF and water as representative solvents of various polarity (ESI, Table S2†). DFT and TDDFT calculations using CAM-B3LYP/6-31G** level of theory resulted in absorption wavelengths that are in good agreement with the experimental data (maximal 30 nm deviation). Trends based on solvent (red-shift from hexane to THF and blue-shift in water) or molecular changes (red-shift with increasing donor strength) were largely well-described. Calculated emission wavelengths (based on optimized S1 excited state geometries) for 1–4 in hexane and for 4 in water and THF were in good agreement with the experimental data (1–3 were not emissive in water). A conformer and tautomer search did not reveal any likely candidates for the dual emission of 4, supporting the hypothesis of LE/ICT competition. The red-shifted emission with increasing solvent polarity was correctly represented in the calculations. However, it was notably overestimated, indicating an excessive stabilization of the excited state (for 1–3). Trends in *λ*_Emax_ based on molecular modifications were well-represented in the calculated data (except for 1 in THF). Good starting geometries with explicit solvent molecules hydrogen-bonded (as both, hydrogen bond donor and -acceptor) to the compound of interest were crucial for reliable computational results. Natural transition orbital analysis showed that the lowest transition corresponds to a HOMO–LUMO excitation, where the HOMO is mainly localized on the amine nitrogen and the benzo-moiety of the BTD, whereas the LUMO is BTD-centred (ESI, Table S3†). This indicated charge-transfer from the amine/amide substituent to the BTD acceptor for absorption into the first excited state. Comparison of optimized ground state (S0) and excited state (S1) structures of 1–4 in hexane further supported the putative ICT (ESI, Fig. S26†). Planarization of the amine moiety (coplanar to BTD) and shortening of the BTD–N bond in S1 indicated a strong sp^2^ character of the amine nitrogen after excitation. Furthermore, significant elongation of the BTD S–N bonds (*ca.* 4%) showed that the heterocycle acts as electron acceptor in the excited state. Similar effects on BTD-based fluorophores have previously been computed and discussed in literature.^29^ The length of the intramolecular hydrogen bond in compounds 1, 2 and 4 was significantly shortened (*ca.* 5%) in S1 as compared to their corresponding S0 structure, which is in line with the other observations.

### Cell studies

**Fig. 3:**
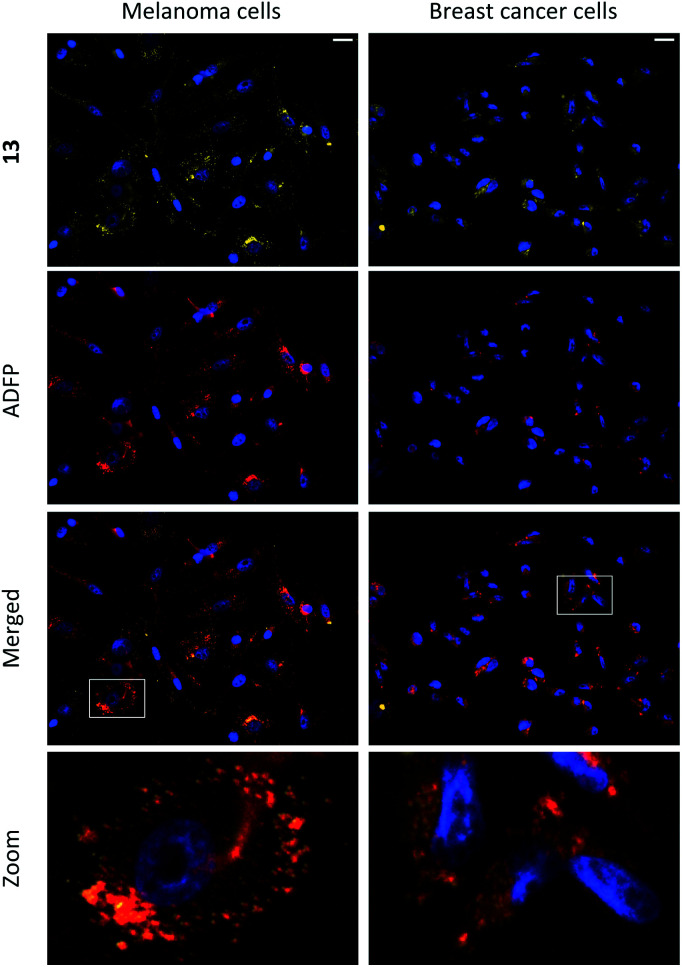
Fluorescence microscopy images of the colocalization of 13 (seen in yellow; ex. 460–500 nm; em. 512–542 nm) and anti-ADFP antibody (1 : 1000; seen in red; ex. 590–650 nm; em. 662–738 nm) in melanoma (SK-MEL-28) and breast cancer (MDA-MB-231) cells. Scale bar 20 μm. Staining was performed on live cells, which were fixed after incubation (10 μM, 24 h) and then subjected to immunocytochemistry. Cell nuclei were stained with DAPI (seen in blue; ex. 325–375 nm; em. 435–485 nm).

Compounds 1–15 were investigated as imaging agents for fluorescence cell microscopy using two different cancer cell lines: SK-MEL-28 (melanoma) and MDA-MB-231 (breast cancer). Initial compound screening of live cells (30 μM, 24 h incubation) gave three potential imaging candidates (11, 12 and 13) that showed distinct punctate staining patterns in the green channel^33^ of the fluorescence microscope. These compounds were further submitted to immunocytochemical colocalization with markers for various cellular organelles (lipid droplets, mitochondria, endosomes, lysosomes, endoplasmic reticulum, and Golgi; ESI, Fig. S33–S37†). As exemplified by 13 (Fig. 3), the results showed colocalization with an ADFP antibody, which targets an enzyme that is prevalent on the surface on lipid droplets. The initial cell experiments were performed at 30 μM to compensate for the rather low brightness of the compounds. However, due to precipitation of 13, the concentration in all further experiments was reduced to 10 μM, which was proven to be sufficient for cell imaging. The lipid droplet distribution in cells treated with 13 seemed to be more located around the nucleus (Fig. 3), whereas LDs stained with 11 or 12 were distributed in a uniform manner over the entire cell (ESI, Fig. S31 and S32†).

**Fig. 4:**
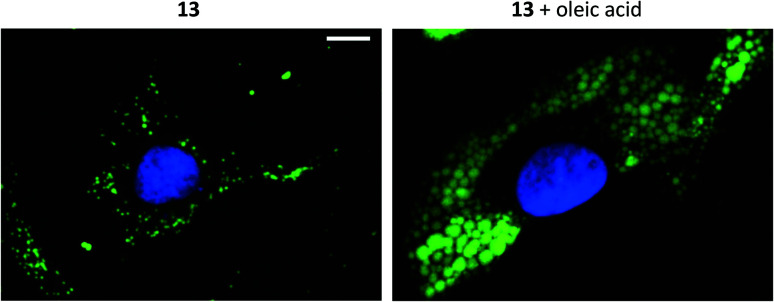
Fluorescence microscopy images of melanoma (SK-MEL-28) cells stained with 13 (seen in green; ex. 460–500 nm; em. 512–542 nm) in the absence or presence of lipid supplementation using oleic acid (100 μM, 24 h). Scale bar 10 μm. Staining with 13 (10 μM, 1 h) was performed on fixed cells. Cell nuclei were stained with DAPI (seen in blue; ex. 325–375 nm; em. 435–485 nm).

Furthermore, the cell viability after treatment with 11–13 was investigated using the resazurin assay (ESI, Fig. S38 and S39†). Compounds 11 and 12 showed no cytotoxic effects in the tested cancer cell lines at 10 μM concentration after 24 h incubation. The viability measurements of compound 13 indicated some level of toxicity, although with high statistical uncertainty after performing multiple replicates. Due to this, the possibility of staining fixed cells was explored. Staining cells with 11, 12 and 13 (10 μM, 1 h) after fixation resulted in fluorescence images with high intensity and good signal-to-background ratios (*e.g.*, 13, Fig. 4, left).

Addition of oleic acid to the cell culture medium is known to stimulate lipid accumulation in cells.^34–36^ Live cells were therefore supplemented with oleic acid (100 μM, 24 h) and fixed prior treatment with 11, 12 or 13 (10 μM, 1 h). The growth of lipid droplets could be visualized clearly in melanoma cells compared to cells without fatty acid supplementation (Fig. 4 and ESI, Fig. S28–S30†). The effects on the breast cancer cell line, which presumably contains lower amounts of LDs, were less distinct (ESI, Fig. S30†). These results further support that compounds 11–13 indeed are specific for staining lipid droplets in cancer cells. Moreover, we noticed that only the most lipophilic structures in the series were suitable for cell imaging (*i.e.*, as LD-specific dyes). Therefore, the octanol/water partition coefficients (log *P*) were calculated^37^ and for the less hydrophobic compounds also experimentally determined (ESI, Table S1†). The calculated data largely matched the experimental log *P* values (maximal deviation of 0.6). The calculated values without an experimental counterpart were therefore considered as adequate approximations. As expected, most of the compounds were less lipophilic than LD-BTD1 (calculated log *P* = 3.9). Moreover, a log *P* ≥ 4, as determined for 11–13, seemed to be required for lipid droplet accumulation – referring to the studied compounds herein (*i.e.*, 1–15).

### Further photophysical studies of the lipid droplet-specific dyes 11–13

The LD-specific dyes 11–13 were further studied for their environmental sensitivity to lipophilic media using concentrations of sodium dodecyl sulfate (SDS), below and above the critical micelle concentration (*ca.* 10 mM)^38^ in a 5% DMSO/water mixture. Compounds 11 and 12 exhibited increased fluorescence intensity in the presence of SDS, with more intense emission at higher SDS concentrations (ESI, Fig. S22 and S23†) – an effect that has previously been reported for BTD derivatives that stain lipophilic structures in cells.^21,29,39^ In contrast, 13 initially aggregated in the presence of SDS (4 mM), which resulted in increased emission intensity (ESI, Fig. S24†). However, a significant gradual decrease of the intensity was observed at higher SDS concentrations (8 and 12 mM). To further probe the aggregation behaviour, additional experiments were performed in DMSO/water mixtures with increasing fractions of water (*f*_w_). Measurements of 13 showed diminished fluorescence intensity with increasing *f*_w_ in the range of 0–80%, while *f*_w_ values of 90% and 95% engendered aggregation-induced emission (AIE)^40,41^ (see ESI, Fig. S24†). This AIE effect has been described for similar BTD structures.^12,20,39,42–44^ Compounds 11 and 12 did not exhibit this behaviour; their fluorescence intensity only decreased with increasing *f*_w_ (ESI, Fig. S22 and S23†). Based on these results, we concluded that SDS strongly affects the aggregation behaviour of 13. In addition, the photostability of compounds 11–13 was investigated in toluene using continuous irradiation for 30 min. Compound 13 showed excellent photostability while the emission intensity of 11 and 12 decreased to approximately 85% of its original value (ESI, Fig. S21†).

## Conclusions

We have described the synthesis, photophysical characterization and cell imaging utility of a series 4-*N*-substituted BTD derivatives. Measurements in solvents of different polarity revealed strong solvatochromism, large Stokes shifts and fluorescence quenching with increasing solvent polarity. Three compounds (11–13) were successfully applied as fluorescent probes for imaging lipid droplets in melanoma and breast cancer cells. These dyes displayed high signal-to-background ratios, in both live and fixed cells, and their lipid droplet specificity was found to correlate with high lipophilicity (log *P* ≥ 4).

## Conflicts of interest

There are no conflicts to declare.

## Supplementary Material

RA-012-D2RA01404A-s001
