# Supplementary material for: Photophysical characterization and fluorescence cell imaging applications of 4-N-substituted benzothiadiazoles
Source: RSC Adv. 2022 May 13;12(23):14544–50. doi: 10.1039/d2ra01404a (PMC9101220; doi:10.1039/d2ra01404a)
Supplement: RA-012-D2RA01404A-s001 [file RA-012-D2RA01404A-s001.pdf]

Electronic Supplementary Material (ESI) for RSC Advances.
This journal is © The Royal Society of Chemistry 2022

Supporting Information

Photophysical characterization and fluorescence cell imaging applications of 4-N-substituted benzothiadiazoles

Susanne Doloczki,[a] Karl O. Holmberg,[b] Ignacio Fdez. Galván,[a] Fredrik J. Swartling[b] and Christine Dyrager*,[a]

[a] Department of Chemistry – BMC, Uppsala University, Box 576, 75123 Uppsala, Sweden.
[b] Department of Immunology, Genetics and Pathology, Rudbeck Laboratory, Uppsala University, 75185 Uppsala, Sweden.

Contents

I. General Information 3
II. Synthesis of the Benzothiadiazole Derivatives **2-15** 7
4-(Methylamino)-2,1,3-benzothiadiazole (**2**).................... 7
4-(Dimethylamino)-2,1,3-benzothiadiazole (**3**) 7
4-(Acetamido)-2,1,3-benzothiadiazole (**4**).................... 7
4-(Butyramido)-2,1,3-benzothiadiazole (**5**) 8
General procedure for the synthesis of compounds **6-12** 8
4-(1-Piperidinyl)-2,1,3-benzothiadiazole (**6**).................... 8
4-(4-Morpholinyl)-2,1,3-benzothiadiazole (**7**) 9
4-(4-Thiomorpholinyl)-2,1,3-benzothiadiazole (**8**) 9
4-(4-Methylpiperazinyl)-2,1,3-benzothiadiazole (**9**).................... 9
4-(1-Pyrrolidinyl)-2,1,3-benzothiadiazole (**10**).................... 9
4-(Cyclohexylamino)-2,1,3-benzothiadiazole (**11**) 10
4-(Phenylamino)-2,1,3-benzothiadiazole (**12**) 10
4-(Diphenylamino)-2,1,3-benzothiadiazole (**13**).................... 10
4-(*L*-Phenylalanino)-2,1,3-benzothiadiazole (**14**).................... 11
4-(*L*-Valino)-2,1,3-benzothiadiazole (**15**) 11
III. Photophysical Data 12
IV. Lipophilicity (logP) Data 36
V. Computational Results.................... 36
VI. Fluorescence Cell Imaging Pictures 39
VII. NMR Spectra 50
VIII. Optimized structures from computations 65
IX. References and Notes.................... 89

I. General Information

Compounds **2**,[1] **3**,[2] **4**,[3] **5**[4] and **10**[5] have previously been reported in the literature.

Materials. All reagents were purchased from Sigma-Aldrich (Merck) or Fluorochem and used without further purification unless otherwise stated. Solvents used for synthesis or photophysical characterization were obtained from Sigma-Aldrich (Merck) or VWR Chemicals and used without further purification unless otherwise indicated. Toluene was distilled prior to use. Aqueous solutions for spectroscopic measurements were prepared with 5% DMSO in distilled water to enhance compound solubility. 4-Amino-2,1,3-benzothiadiazole (**BTD-NH$_2$**, **1**) was purchased from Sigma-Aldrich (Merck) and purified by sublimation before photophysical characterization and cell studies.

Chromatography. Thin-layer chromatography (TLC) was carried out using aluminum plates coated with silica gel (60F$_{254}$, Merck) and visualized through exposure to UV light (λ = 254 nm and 365 nm). Purification by column chromatography was performed using silica gel (particle size 40–63 µm, VWR Chemicals).

Chemical characterization. NMR spectra were recorded at 298 K using an Agilent MR400-DD2 instrument at 400 MHz (^{1}H) and 101 MHz (^{13}C). Chemical shifts (δ) are reported in ppm using the residual solvent peak in $CDCl_3$ (δ_H = 7.26 and δ_C = 77.16 ppm) as internal reference.[6] Coupling constants (*J*) are given in Hz and the apparent resonance multiplicity is reported as s (singlet), d (doublet), t (triplet), sext (sextet) or m (multiplet). High-resolution mass spectrometry (HRMS) data (ESI-TOF and APCI) was determined at the Division of Mass Spectrometry, Department of Chemistry, Imperial College London, UK.

Photophysical characterization. UV-vis absorption spectra were acquired on a UV-1650PC Shimadzu instrument at room temperature using quartz cuvettes (10 mm). Absorption maxima (λ_{Amax}) are reported in nm and the molar extinction coefficient (ε) in M^{-1} cm^{-1} with a margin of error of up to 3%. For each compound three data points with known different concentrations were acquired and the measured absorbances (≤ 1.0) were plotted against the concentrations. The molar extinction coefficients were then determined according to the Beer-Lambert law as the slope of the linear fit. Fluorescence measurements were carried out using a Spex Fluorolog 1680 0.22m Double Spectrometer instrument (experiments shown in Figures S21-24 were performed using a Shimadzu RF-6000 spectrofluorophotometer). Fluorescence quantum yields (Φ_F) were determined relative to fluorescein in 20 mM aq. NaOH (Φ_F = 0.93)[7] or quinine sulfate in 0.1 M aq. H_2SO_4 (Φ_F = 0.55)[8]. Three fluorescence spectra were recorded per compound per solvent, and integrated peak areas were plotted against the absorbances at the excitation wavelength. The quantum yields were then calculated from the slope of the linear fit *via* the comparative method, with a margin of error of up to 10%. Refractive indices of solvents were adjusted for quantum yield calculations based on the excitation wavelength used.[9-11] The excitation wavelength (360 or 430 nm) and concentrations for the quantum yield measurements were selected so that the absorbance was below 0.1 to prevent self-absorption effects. Obtained raw data was processed using OriginPro 8 software. Absorption and emission spectra illustrated herein were normalized to the peak maximum of interest. Additionally, emission spectra were smoothed using an FFT filter function with 5 point window. Spectra with weak emission and low signal-to-noise ratio (Φ_F < 0.03) were smoothed using an FFT filter function with 30 point window.

Photostability measurements. Photostability experiments (spectra and time course) were performed on a Shimadzu RF-6000 spectrofluorophotometer. The compound (**11**, **12**, or **13**) was dissolved in toluene and the concentration was adjusted to correspond to an absorbance value at 0.10 (λ_{abs} = 445 nm). The solution (2.50 mL) was transferred to a fluorescence cuvette (10x10x35 mm) and a fluorescence spectrum was measured (λ_{ex} = 445 nm, ex./em. bandwidth = 5/5 nm). The sample was then irradiated in the fluorometer for 6 × 5 min periods (*i.e.*, total irradiation time = 30 min). Between these periods the cuvette was briefly shaken to ensure that the measured intensity values represent a homogenous sample solution. The initial intensity of each irradiation period was plotted against the corresponding accumulated irradiation time. Fluorometer settings: λ_{ex} = 445 nm; λ_{em} = 554 (**11**), 544 (**12**) or 573 nm (**13**); ex./em. bandwidth = 20/1 nm, accumulation time = 50 ms. A new fluorescence spectrum was measured after the total irradiation time (30 min).

Determination of lipophilicity. The octanol/water partition coefficient (logP) of most compounds (**1-5**, **7**, **9**, **14**, **15**) was determined using the shake-flask method. The compound was dissolved in octanol/water 1:1 (4.0 mL) followed by vigorous shaking. After phase separation, a 0.5 mL sample was taken from the octanol phase and 1.0 mL from the water phase. Octanol (0.5 mL), water (1.0 mL) and methanol (3.0 mL) were added to the octanol sample, while octanol (1.0 mL) and methanol (3.0 mL) were added to the water sample. Thus, the final solvent mixture of octanol/water/methanol was 1:1:3 for both samples. Subsequently, the absorption spectra of both samples were recorded and the logP value was determined as

$$\mathrm{logP} = \log\left(\frac{2\,\mathrm{Abs_{octanol}}}{\mathrm{Abs_{water}}}\right).$$

Reported experimental logP values represent the average of triplicates. LogP values for **6**, **8** and **10-13** were calculated instead due to poor water solubility. The Molinspiration Cheminformatics web service was used to calculate logP values.[12] Nevertheless, comparison of calculated and experimental logP values indicated that the calculated values are reasonably good approximations (deviations ≤ 0.6).

Computational details. All density functional theory (DFT) calculations were performed using Gaussian 09[13] software and CAM-B3LYP/6-31G** level of theory. DFT was applied to ground states and time-dependent density functional theory (TDDFT) was applied to excited states. To cover a wide range of solvent polarity, calculations of **1-4** were carried out for solutions in hexane, THF and water. The polarizable continuum model (PCM) was used to account for implicit solvation effects. Explicit solvent effects in terms of hydrogen bonding were allowed for by defining one explicit solvent molecule of THF or water. For **1** and **2** two explicit water molecules were considered; one as hydrogen bond donor, the other as hydrogen bond acceptor (see Figure S25). Vertical transitions (ten excited states) were generally calculated with state-specific solvation. Optimized ground-state geometries were used for absorption and optimized excited-state geometries were used for emission. Optimized geometries were confirmed as energetic minimum by vibrational frequency calculations. Natural transition orbitals of **1-4** were calculated in hexane. To compare the pyramidalization of the amine nitrogen in compounds **6** and **10**, geometry optimizations were performed in hexane using the described method. Hydrogen bond energies were calculated at the same level of theory but without implicit solvation using one explicit water molecule either as hydrogen bond donor (for tertiary amines) or as hydrogen bond acceptor (secondary amines). For primary amine **1**, two explicit water molecules were computed (both as hydrogen bond acceptors) and the resulting energy was divided by two to obtain the averaged hydrogen bond energy per water molecule. The basis set superposition

error was corrected by applying the counterpoise method. Computed structures and orbitals were visualized using Avogadro 1.2.0.[14-15]

Cell cultures. SK-MEL-28 and MDA-MB-231 were grown in Dulbecco's Modified Eagle Medium (DMEM), high glucose, GlutaMAX™ (Thermo Fisher Scientific, Cat#61965026) supplemented with 10% heat inactivated Fetal Bovine Serum (Thermo Fisher Scientific, Cat#10270106) and Penicilin-Streptomycin (Sigma-Aldrich, Cat#P0781) using 100mm TC treated dishes (VWR, Cat#734-0006) and a humidified incubator at 37 °C with 5% CO_2. Cells were passaged using Trypsin – EDTA Solution (Sigma-Aldrich, Cat#T3924) after washing with Dulbecco's Phosphate-Buffered Saline (DPBS) (Thermo Fisher Scientific, Cat#14190094).

Cell viability assay. Cells were counted using a Countess II Automated Cell Counter (Thermo Fisher Scientific, Cat#AMQAX1000) and seeded in 96 well plates, 10 000 live cells/well, the day before treatment. Compounds were dissolved in dimethyl sulfoxide (DMSO) (Sigma-Aldrich, Cat#317275) and further diluted in extra cell culture medium before cell treatment. Cells were incubated with compounds for 24 hours at a final concentration of 10 µM or 30 µM (*i.e.*, 0.2 vol% or 0.6 vol% DMSO) with DMSO treated cells as control. Cell viability was analyzed after 2.5 hours incubation with Resazurin Reagent (1:10) (Sigma-Aldrich, Cat#R7017) using a Synergy™ HTX Multi-Mode Reader (BioTek), and the fluorescence was detected by excitation at 530 nm and emission at 590 nm. Experiments were run in sextuplets and repeated three to six times.

Cell staining and fluorescence microscopy. SK-MEL-28 and MDA-MB-231 cells were counted (as mentioned above) and seeded in chamber slides (Sarstedt, Cat#94.6140.802) the day before treatment at 80 000 live cells/well. Stock solutions of the compounds in DMSO were prepared at 5.0 mM concentration, which was diluted with cell medium to the indicated final compound concentration used. Live cells were incubated with compounds at a final concentration of 10 µM or 30 µM for 24 hours with DMSO treated cells as control (*i.e.*, 0.2 vol% or 0.6 vol% DMSO). Subsequent fixation was performed using a 4% Buffered Formaldehyde Solution (Histolab Cat#02176) for 15 minutes at room temperature, followed by incubation with DAPI (1:1000) (Sigma-Aldrich, Cat#D9564) diluted in Tris-Buffered Saline (VWR, Cat# 97062-370) with 1% TWEEN® 20 (Sigma-Aldrich, Cat#P1379) (TBST) for 15 minutes at room temperature. Cells not stained with DAPI were incubated with TBST alone. Slides were then washed in TBST and mounted using Fluoromount (Sigma-Aldrich, Cat#F4680). For the staining of fixated cells, cells were first fixated (as described above) two days after seeding, permeabilized using 0.2% Triton X/ PBS for 5 min at 4 °C,[16] washed with PBS (×2), and then incubated with the compounds in PBS (10 µM; *i.e.*, 0.2 vol% DMSO) for 1 h at 37 °C, followed by washing with PBS and incubation with DAPI (1:1000) diluted in TBST for 15 min at room temperature, washing in TBST and mounting. For lipid supplementation experiments, a stock solution of oleic acid in DMSO was prepared at 50 mM concentration and either added to live cells (100 µM final concentration) and incubated for 24 h together with the compounds (10 µM; *i.e.*, 0.4 vol% DMSO in total) or added to live cells, incubated for 24 h (*i.e.*, 0.2 vol% final DMSO concentration) and then following the procedure for cell fixation and staining of fixated cells. Staining was performed at least twice for each compound to ensure reproducibility. Fluorescence cell imaging pictures were taken using a Leica DMi8 microscope.[17] The acquired raw data was processed using ImageJ (Fiji distribution).[18-19] Background subtraction, brightness and contrast adjustments were made to improve visibility.

Colocalization experiments. Organelle localization was investigated using the Organelle Localization IF Antibody Sampler Kit from Cell Signaling (Cat#8653T) and Anti-ADFP antibody [EPR3713] from Abcam (ab108323).[20] Anti-rabbit IgG Fab2 Alexa Fluor® 647 from Cell Signaling (Cat#4414S) was used as secondary antibody. The procedure for staining living cells (as mentioned above) was followed (10 µM or 30 µM final compound concentration). After fixation, cells were permeabilized with 0.2% Triton X/ PBS for 5 min at 4 °C, washed with PBS (×2), and blocked with 0.5% BSA/ PBS for 30 min at room temperature. The cells were then incubated with the primary antibody diluted in 0.5% BSA/ PBS (200 µL; 1:1000) for 2 h at room temperature, washed with PBS (3 × 5 min), followed by incubation with secondary antibody (1:1000) together with DAPI (1:1000) diluted in 0.5% BSA/ PBS (200 µL) for 1 h at room temperature, washing with PBS (3 × 5 min) and mounting. Fluorescence microscopy and data processing was performed as described above.

II. Synthesis of the Benzothiadiazole Derivatives 2-15

4-(Methylamino)-2,1,3-benzothiadiazole (2)

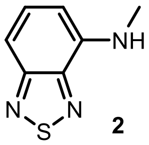
2

The procedure was adapted from reported literature[21] as follows: methyl iodide (0.11 mL, 1.77 mmol, 2.7 equiv) was added to a solution of 4-amino-2,1,3-benzothiadiazole (99 mg, 0.66 mmol, 1.0 equiv) and K_2CO_3 (380 mg, 2.75 mmol, 4.2 equiv) in dimethylformamide (2.0 mL). The reaction mixture was stirred at 50 °C for 20.5 h. After cooling down to rt, saturated aq. $NaHCO_3$ solution (25 mL) was added and the aqueous layer was extracted with EtOAc (4 × 15 mL). The combined organic layers were washed with brine (20 mL), dried over Na_2SO_4 and concentrated under reduced pressure. To remove dimethylformamide residues, the crude material was dissolved again in EtOAc (20 mL) and washed thoroughly with brine (4 × 25 mL). The organic layer was dried over Na_2SO_4 and concentrated under reduced pressure. Purification by column chromatography (hexane/EtOAc 96:4 to 9:1, R_f = 0.42) gave **2** as an orange solid (39 mg, 36%). ^{1}H NMR (400 MHz, $CDCl_3$) δ 7.46 (dd, *J* = 8.8, 7.4 Hz, 1H), 7.24 (dd, *J* = 8.8, 0.8 Hz, 1H), 6.33 (d, *J* = 7.4 Hz, 1H), 5.26 (s_{br}, 1H), 3.04 (d, *J* = 4.9 Hz, 3H). ^{13}C NMR (101 MHz, $CDCl_3$) δ 155.9, 148.0, 141.5, 132.0, 108.3, 101.0, 30.2. HRMS (ESI-TOF) calcd for $[C_7H_7N_3S + H]^+$ 166.0439; found 166.0443.

4-(Dimethylamino)-2,1,3-benzothiadiazole (3)

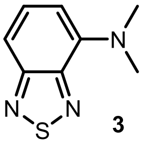
3

The procedure was adapted from reported literature[22] as follows: 4-amino-2,1,3-benzothiadiazole (97 mg, 0.64 mmol, 1.0 equiv) was dissolved in glacial acetic acid (12 mL) under nitrogen atmosphere. Paraformaldehyde (366 mg, 12.2 mmol, 19 equiv) was added followed by $NaBH_3CN$ (457 mg, 7.27 mmol, 11 equiv). The reaction mixture was stirred at rt for 26 h. The mixture was carefully basified with 3 M aq. NaOH solution (75 mL) and then extracted with CH_2Cl_2 (4 × 20 mL). The combined organic layers were dried over Na_2SO_4 and concentrated under reduced pressure. Purification by column chromatography (hexane/EtOAc 96:4, R_f = 0.29; then pentane/Et_2O 95:5, R_f = 0.35) gave **3** as an orange-red oil (49 mg, 42%). ^{1}H NMR (400 MHz, $CDCl_3$) δ 7.47 – 7.41 (m, 2H), 6.56 (d_{br}, *J* = 6.7 Hz, 1H), 3.27 (s, 6H). ^{13}C NMR (101 MHz, $CDCl_3$) δ 157.0, 149.4, 144.5, 130.9, 111.3, 109.1, 42.7. HRMS (ESI-TOF) calcd for $[C_8H_9N_3S + H]^+$ 180.0595; found 180.0598.

4-(Acetamido)-2,1,3-benzothiadiazole (4)

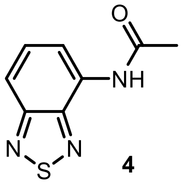
4

The procedure was adapted from reported literature[23] as follows: pyridine (0.10 mL, 1.24 mmol, 2.0 equiv) was added to a solution of 4-amino-2,1,3-benzothiadiazole (94 mg, 0.62 mmol, 1.0 equiv) in dry CH_2Cl_2 (1.0 mL) under nitrogen atmosphere. The mixture was cooled down to 0 °C before acetyl chloride (52 µL, 0.73 mmol, 1.2 equiv) was added dropwise. The reaction mixture was then allowed to reach rt and further stirred for 3.5 h. CH_2Cl_2 (1 mL) was added and the mixture was washed with aqueous HCl solution (1 M, 1.5 mL), water (1.5 mL) and brine (1.5 mL). The organic layer was dried over Na_2SO_4 and concentrated under reduced pressure. Purification by column chromatography (hexane/EtOAc 7:3, R_f = 0.28) gave **4** as a colorless, crystalline solid (111 mg, 92%). ^{1}H NMR (400 MHz, $CDCl_3$) δ 8.47 (d_{br}, *J* = 7.3 Hz, 2H), 7.67 (dd, *J* = 8.9, 1.0 Hz, 1H), 7.59 (dd, *J* = 8.9, 7.3 Hz, 1H), 2.34 (s,

3H). ^{13}C NMR (101 MHz, $CDCl_3$) δ 168.8, 154.9, 147.8, 131.3, 130.1, 115.9, 115.1, 25.0. HRMS (ESI-TOF) calcd for $[C_8H_7N_3OS + H]^+$ 194.0388; found 194.0395.

4-(Butyramido)-2,1,3-benzothiadiazole (5)

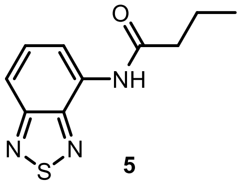

5

The procedure was adapted from reported literature[23] as follows: pyridine (0.11 mL, 1.37 mmol, 2.1 equiv) was added to a solution of 4-amino-2,1,3-benzothiadiazole (99 mg, 0.66 mmol, 1.0 equiv) in dry CH_2Cl_2 (1.0 mL) under nitrogen atmosphere. The mixture was cooled down to 0 °C before butyryl chloride (90 µL, 0.87 mmol, 1.3 equiv) was added dropwise. The reaction mixture was allowed to reach rt and was then further stirred for 2.5 h. CH_2Cl_2 (5 mL) was added and the mixture was washed with aqueous HCl solution (1 M, 5 mL), water (5 mL) and brine (5 mL). The organic layer was dried over Na_2SO_4 and concentrated under reduced pressure. Purification by column chromatography (hexane/CH_2Cl_2 1:3 to 0:1, R_f (CH_2Cl_2) = 0.38) gave **5** as a yellowish solid (94 mg, 65%). ^1H NMR (400 MHz, $CDCl_3$) δ 8.48 (d, J = 7.3 Hz, 1H), 8.45 (s_{br}, 1H), 7.65 (dd, J = 8.9, 1.1 Hz, 1H), 7.57 (dd, J = 8.9, 7.3 Hz, 1H), 2.51 (t, J = 7.4 Hz, 2H), 1.83 (sext, J = 7.4 Hz, 2H), 1.05 (t, J = 7.4 Hz, 3H). ^{13}C NMR (101 MHz, $CDCl_3$) δ 171.8, 154.9, 147.8, 131.3, 130.1, 115.7, 115.0, 39.9, 19.1, 13.9. HRMS (ESI-TOF) calcd for $[C_{10}H_{11}N_3OS + H]^+$ 222.0701; found 222.0701.

General procedure for the synthesis of compounds 6-12

The procedure was adapted from reported literature[24] as follows: 4-bromo-2,1,3-benzothiadiazole (100 mg, 0.47 mmol, 1.0 equiv) and the relevant amine (0.51 mmol, 1.1 equiv) were added to a suspension of *t*-BuOK (63 mg, 0.56 mmol, 1.2 equiv) and PEPPSI-IPr (7 mg, 0.01 mmol, 2 mol%) in dry toluene (1.6 mL). The reaction mixture was stirred at 120 °C for 3.5 h under nitrogen atmosphere, unless otherwise specified. The mixture was allowed to cool down to rt, followed by filtration through Celite. The solvent was removed under reduced pressure and the crude product was purified by column chromatography.

4-(1-Piperidinyl)-2,1,3-benzothiadiazole (6)

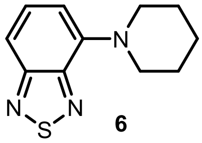

6

The compound was synthesized according to the general procedure described above using piperidine (51 µL, 0.51 mmol, 1.1 equiv). Purification by column chromatography (hexane/EtOAc 96:4, R_f = 0.28; then hexane/CH_2Cl_2 4:1 to 1:1) gave **6** as an orange-brown solid (86 mg, 85%). ^1H NMR (400 MHz, $CDCl_3$) δ 7.50 (dd, J = 8.7, 1.1 Hz, 1H), 7.44 (dd, J = 8.7, 7.2 Hz, 1H), 6.72 (dd, J = 7.2, 1.1 Hz, 1H), 3.49 – 3.46 (m, 4H), 1.87 – 1.82 (m, 4H), 1.71 – 1.65 (m, 2H). ^{13}C NMR (101 MHz, $CDCl_3$) δ 156.8, 150.1, 145.5, 130.7, 113.1, 111.5, 51.8, 26.1, 24.7. HRMS (ESI-TOF) calcd for $[C_{11}H_{13}N_3S + H]^+$ 220.0906; found 220.0908.

4-(4-Morpholinyl)-2,1,3-benzothiadiazole (7)

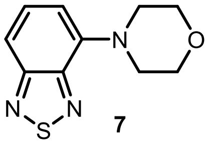

7

The compound was synthesized according to the general procedure, on half the scale described above, using morpholine (22 µL, 0.25 mmol, 1.1 equiv). Purification by column chromatography (hexane/EtOAc 9:1, R_f = 0.21) gave **7** as a yellow-orange solid (37 mg, 73%). ^{1}H NMR (400 MHz, $CDCl_3$) δ 7.57 (dd, *J* = 8.8, 0.9 Hz, 1H), 7.48 (dd, *J* = 8.8, 7.3 Hz, 1H), 6.75 (d, *J* = 7.3 Hz, 1H), 4.01 – 3.98 (m, 4H), 3.56 – 3.53 (m, 4H). ^{13}C NMR (101 MHz, $CDCl_3$) δ 156.8, 149.7, 144.1, 130.5, 114.2, 111.5, 67.0, 50.7. HRMS (ESI-TOF) calcd for $[C_{10}H_{11}N_3OS + H]^+$ 222.0701; found 222.0707.

4-(4-Thiomorpholinyl)-2,1,3-benzothiadiazole (8)

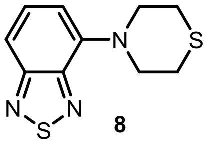

8

The compound was synthesized according to the general procedure described above using thiomorpholine (52 µL, 0.51 mmol, 1.1 equiv). Purification by column chromatography (hexane/EtOAc 96:4, R_f = 0.23) gave **8** as a yellow-orange solid (64 mg, 59%). ^{1}H NMR (400 MHz, $CDCl_3$) δ 7.55 (dd, *J* = 8.7, 1.0 Hz, 1H), 7.46 (dd, *J* = 8.7, 7.3 Hz, 1H), 6.77 (d, *J* = 7.3 Hz, 1H), 3.83 – 3.81 (m, 4H), 2.92 – 2.90 (m, 4H). ^{13}C NMR (101 MHz, $CDCl_3$) δ 156.8, 149.9, 144.5, 130.5, 114.0, 112.4, 53.0, 27.6. HRMS (ESI-TOF) calcd for $[C_{10}H_{11}N_3S_2 + H]^+$ 238.0473; found 238.0474.

4-(4-Methylpiperazinyl)-2,1,3-benzothiadiazole (9)

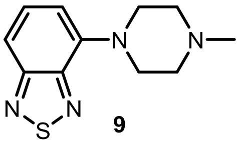

9

The compound was synthesized according to the general procedure described above using 1-methylpiperazine (57 µL, 0.51 mmol, 1.1 equiv). Purification by column chromatography (CH_2Cl_2/MeOH 95:5, R_f = 0.18) gave **9** as a dark yellow solid (59 mg, 54%). ^{1}H NMR (400 MHz, $CDCl_3$) δ 7.54 (dd, *J* = 8.7, 1.0 Hz, 1H), 7.46 (dd, *J* = 8.7, 7.3 Hz, 1H), 6.73 (dd, *J* = 7.3, 1.0 Hz, 1H), 3.58 (t, *J* = 5.0 Hz, 4H), 2.72 (t, *J* = 5.0 Hz, 4H), 2.41 (s, 3H). ^{13}C NMR (101 MHz, $CDCl_3$) δ 156.8, 149.8, 144.3, 130.6, 113.7, 111.6, 55.2, 50.2, 46.3. HRMS (ESI-TOF) calcd for $[C_{11}H_{14}N_4S + H]^+$ 235.1017; found 235.1011.

4-(1-Pyrrolidinyl)-2,1,3-benzothiadiazole (10)

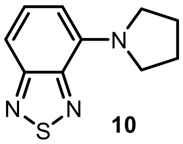

10

The compound was synthesized according to the general procedure described above using pyrrolidine (43 µL, 0.51 mmol, 1.1 equiv). Purification by column chromatography (hexane/EtOAc 96:4, R_f = 0.41) gave **10** as an orange solid (68 mg, 71%). ^{1}H NMR (400 MHz, $CDCl_3$) δ 7.40 (dd, *J* = 8.6, 7.6 Hz, 1H), 7.20 (d, *J* = 8.6 Hz, 1H), 6.23 (d, *J* = 7.6 Hz, 1H), 3.84 – 3.81 (m, 4H), 2.08 – 2.02 (m, 4H). ^{13}C NMR (101 MHz, $CDCl_3$) δ 157.2, 148.5, 141.3, 131.6, 107.3, 104.0, 50.4, 25.7. HRMS (ESI-TOF) calcd for $[C_{10}H_{11}N_3S + H]^+$ 206.0752; found 206.0750.

4-(Cyclohexylamino)-2,1,3-benzothiadiazole (11)

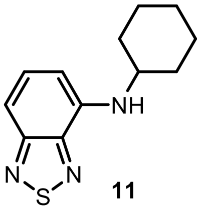

11

The compound was synthesized according to the general procedure described above using cyclohexylamine (60 µL, 0.52 mmol, 1.1 equiv) and stirring the reaction mixture at 120 °C for 4.5 h. Purification by column chromatography (hexane/EtOAc 96:4, R_f = 0.23) gave **11** as an orange-brown oil (76 mg, 70%). ^{1}H NMR (400 MHz, $CDCl_3$) δ 7.41 (dd, *J* = 8.7, 7.5 Hz, 1H), 7.18 (d, *J* = 8.7 Hz, 1H), 6.34 (d, *J* = 7.5 Hz, 1H), 5.18 (d, *J* = 6.3 Hz, 1H), 3.49 – 3.41 (m, 1H), 2.17 – 2.13 (m, 2H), 1.86 – 1.80 (m, 2H), 1.71 – 1.65 (m, 1H), 1.49 – 1.24 (m, 5H). ^{13}C NMR (101 MHz, $CDCl_3$) δ 156.0, 148.1, 139.4, 132.1, 107.5, 101.3, 51.6, 32.9, 26.0, 25.0. HRMS (ESI-TOF) calcd for $[C_{12}H_{15}N_3S + H]^+$ 234.1065; found 234.1072.

4-(Phenylamino)-2,1,3-benzothiadiazole (12)

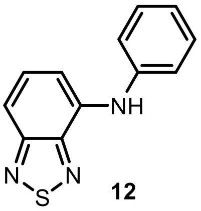

12

The compound was synthesized according to the general procedure described above using aniline (47 µL, 0.52 mmol, 1.1 equiv). The reaction mixture was stirred at 120 °C for 4 h, then allowed to cool down to rt and further stirred overnight. Purification by column chromatography (hexane/CH_2Cl_2 2:1, R_f = 0.20) gave **12** as an orange solid (80 mg, 76%). ^{1}H NMR (400 MHz, $CDCl_3$) δ 7.45 (dd, *J* = 8.8, 7.2 Hz, 1H), 7.42 – 7.38 (m, 3H), 7.36 – 7.33 (m, 2H), 7.15 (dd, *J* = 7.2, 1.1 Hz, 1H), 7.13 – 7.08 (m, 1H); integrals indicate N-H signal at around 7.15 ppm. ^{13}C NMR (101 MHz, $CDCl_3$) δ 155.9, 148.4, 140.7, 136.2, 131.4, 129.7, 123.3, 120.6, 110.9, 104.8. HRMS (ESI-TOF) calcd for $[C_{12}H_9N_3S + H]^+$ 228.0595; found 228.0603.

4-(Diphenylamino)-2,1,3-benzothiadiazole (13)

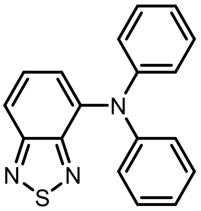

13

The procedure was adapted from reported literature[25] as follows: $Pd_2(dba)_3$ (23 mg, 0.03 mmol, 11 mol%), RuPhos (27 mg, 0.06 mmol, 12 mol%) and Cs_2CO_3 (316 mg, 0.97 mmol, 2.1 equiv) were added to a solution of freshly recrystallized diphenylamine (127 mg, 0.75 mmol, 1.6 equiv) and 4-bromo-2,1,3-benzothiadiazole (100 mg, 0.47 mmol, 1.0 equiv) in dry toluene (6.0 mL). The reaction mixture was stirred at 120 °C for 24 h under nitrogen atmosphere. After cooling down to rt, the mixture was poured into water (40 mL) and extracted with CH_2Cl_2 (4 × 20 mL). The combined organic layers were washed with brine (30 mL), dried over Na_2SO_4 and concentrated under reduced pressure. Purification by column chromatography (hexane/CH_2Cl_2 1:1, R_f = 0.37) gave **13** as an orange solid (49 mg, 35%). ^{1}H NMR (400 MHz, $CDCl_3$) δ 7.73 (dd, *J* = 8.8, 0.9 Hz, 1H), 7.48 (dd, *J* = 8.8, 7.4 Hz, 1H), 7.29 – 7.24 (m, 4H), 7.16 (dd, *J* = 7.4, 0.9 Hz, 1H), 7.09 – 7.04 (m, 6H). ^{13}C NMR (101 MHz, $CDCl_3$) δ 156.8, 151.3, 147.9, 140.3, 130.1, 129.4, 124.3, 123.5, 123.3, 116.7. HRMS (ESI-TOF) calcd for $[C_{18}H_{13}N_3S + H]^+$ 304.0908; found 304.0906.

4-(*L*-Phenylalanino)-2,1,3-benzothiadiazole (14)

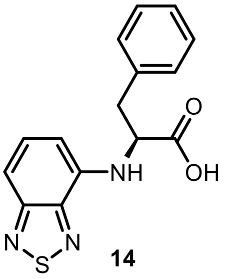

The procedure was adapted from reported literature[26] as follows: CuI (9 mg, 0.047 mmol, 10 mol%) was added to a solution of *L*-phenylalanine (76 mg, 0.46 mmol, 1.0 equiv), 4-bromo-2,1,3-benzothiadiazole (100 mg, 0.46 mmol, 1.0 equiv) and K_2CO_3 (99 mg, 0.71 mmol, 1.5 equiv) in dimethylacetamide (1.6 mL). The reaction mixture was stirred at 90 °C for 5 d. After cooling down to rt, the mixture was diluted with EtOAc (5 mL) and water (2.5 mL). While cooling to ca 10 °C, concentrated HCl solution was added dropwise until pH 2 was reached. The organic layer was separated and the aqueous layer was extracted with EtOAc (5 × 10 mL). The combined organic layers were washed with brine (10 mL) and dried over Na_2SO_4. The solvent was concentrated under reduced pressure and residual dimethylacetamide was partially removed by co-evaporation with toluene. Recrystallization from CH_2Cl_2/hexane (2:1) gave **14** as orange-red crystals (36 mg, 26%). ^{1}H NMR (400 MHz, $CDCl_3$) δ 7.40 (dd, *J* = 8.9, 7.3 Hz, 1H), 7.32 – 7.24 (m, 4H), 6.34 (d, *J* = 7.3 Hz, 1H), 5.68 (s_{br}, 1H), 4.55 (dd, *J* = 6.9, 5.7 Hz, 1H), 3.39 (dd, *J* = 13.9, 5.7 Hz, 1H), 3.26 (dd, *J* = 13.9, 6.9 Hz, 1H). ^{13}C NMR (101 MHz, $CDCl_3$) δ 176.9, 155.7, 147.8, 138.5, 135.7, 131.5, 129.4, 128.9, 127.5, 110.2, 102.9, 57.2, 38.3. HRMS (APCI) calcd for $[C_{15}H_{13}N_3O_2S + H]^+$ 300.0801; found 300.0806.

4-(*L*-Valino)-2,1,3-benzothiadiazole (15)

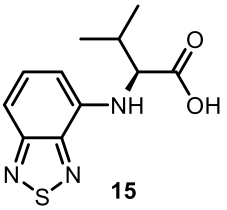

The procedure was adapted from reported literature[26] as follows: CuI (11 mg, 0.058 mmol, 12 mol%) was added to a solution of *L*-valine (54 mg, 0.46 mmol, 1.0 equiv), 4-bromo-2,1,3-benzothiadiazole (101 mg, 0.47 mmol, 1.0 equiv) and K_2CO_3 (104 mg, 0.75 mmol, 1.6 equiv) in dimethylacetamide (1.6 mL). The reaction mixture was stirred at 90 °C for 6 d. After cooling down to rt, the mixture was diluted with EtOAc (7 mL) and water (5 mL). While cooling to ca 10 °C, concentrated HCl solution was added dropwise until pH 2 was reached. The organic layer was separated and the aqueous layer was extracted with EtOAc (4 × 10 mL). The combined organic layers were washed with brine (2 × 20 mL) and dried over Na_2SO_4. The solvent was concentrated under reduced pressure and residual dimethylacetamide was partially removed by co-evaporation with toluene. Purification by column chromatography (CH_2Cl_2/MeOH 10:0 to 9:1, R_f = 0.48; then CH_2Cl_2/acetone/MeOH 100:20:5, R_f = 0.26) gave **15** as a dark orange solid (43 mg, 36%). ^{1}H NMR (400 MHz, $CDCl_3$) δ 7.40 (dd, *J* = 8.8, 7.3 Hz, 1H), 7.29 (dd, *J* = 8.8, 0.8 Hz, 1H), 6.36 (d, *J* = 7.3 Hz, 1H), 5.62 (s_{br}, 1H), 4.08 (d, *J* = 5.6 Hz, 1H), 2.42 – 2.30 (m, 1H), 1.15 (dd, *J* = 13.2, 6.8 Hz, 6H). ^{13}C NMR (101 MHz, $CDCl_3$) δ 177.8, 155.8, 147.9, 139.3, 131.6, 109.7, 102.6, 61.8, 31.4, 19.4, 18.6. HRMS (ESI-TOF) calcd for $[C_{11}H_{13}N_3O_2S + H]^+$ 252.0807; found 252.0810.

III. Photophysical Data

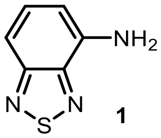

1

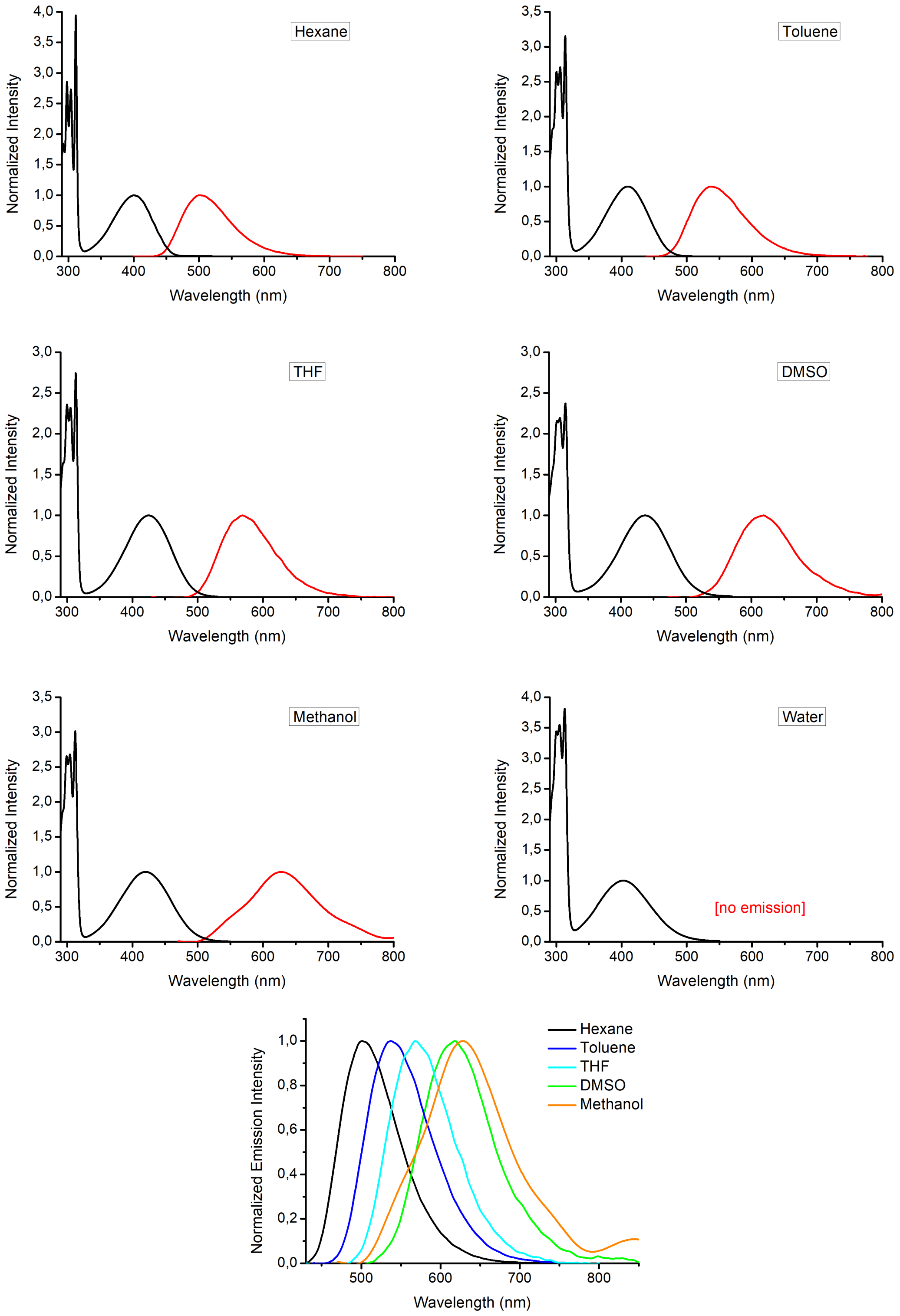

Figure S1. Normalized UV-vis absorption (black) and emission spectra (red) of **1** in solvents of different polarity. Bottom spectrum shows the solvatochromic effect on the emission.

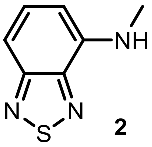

2

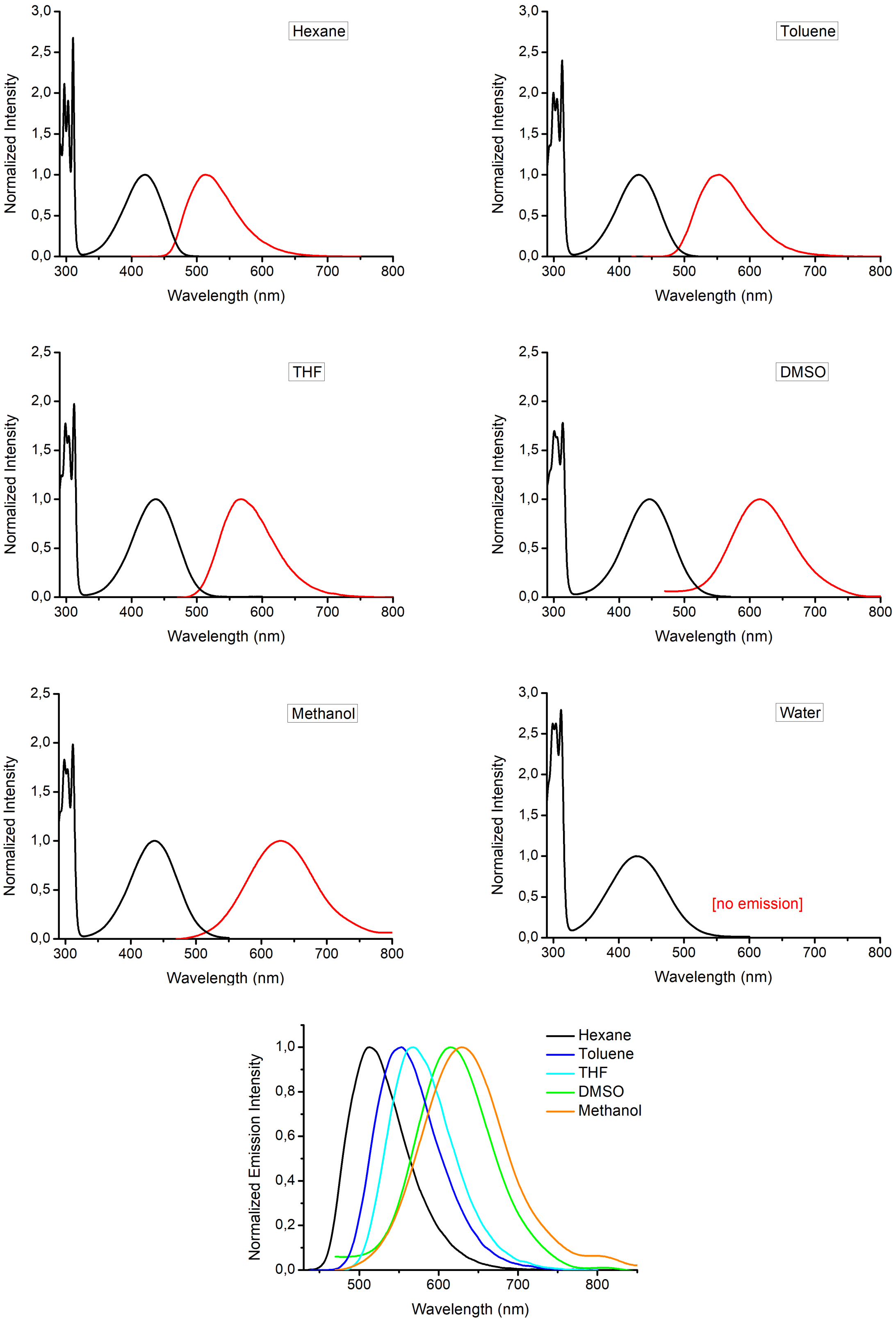

Figure S2. Normalized UV-vis absorption (black) and emission spectra (red) of **2** in solvents of different polarity. Bottom spectrum shows the solvatochromic effect on the emission.

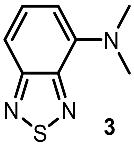

3

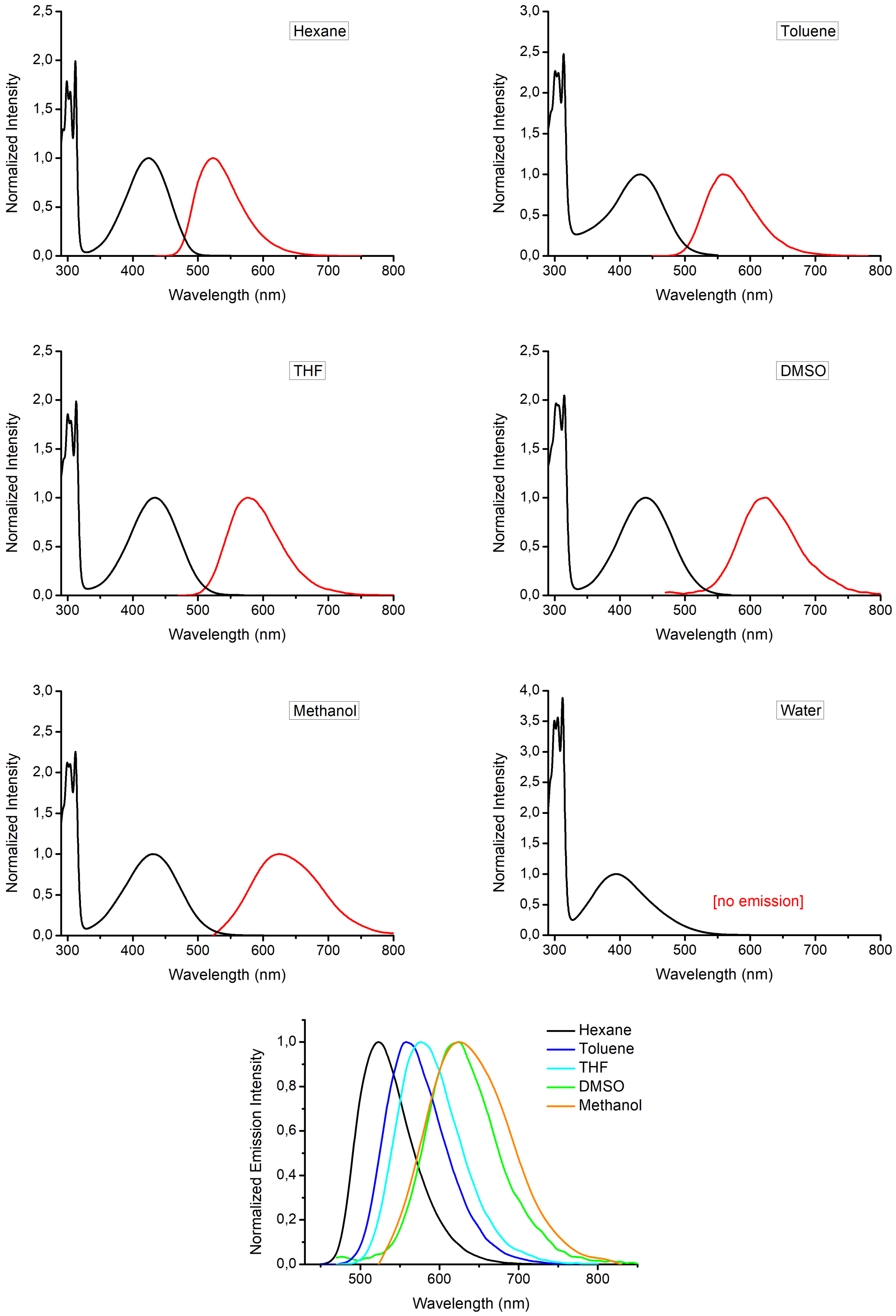

Figure S3. Normalized UV-vis absorption (black) and emission spectra (red) of **3** in solvents of different polarity. Bottom spectrum shows the solvatochromic effect on the emission.

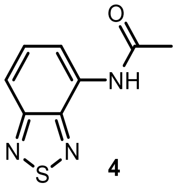

4

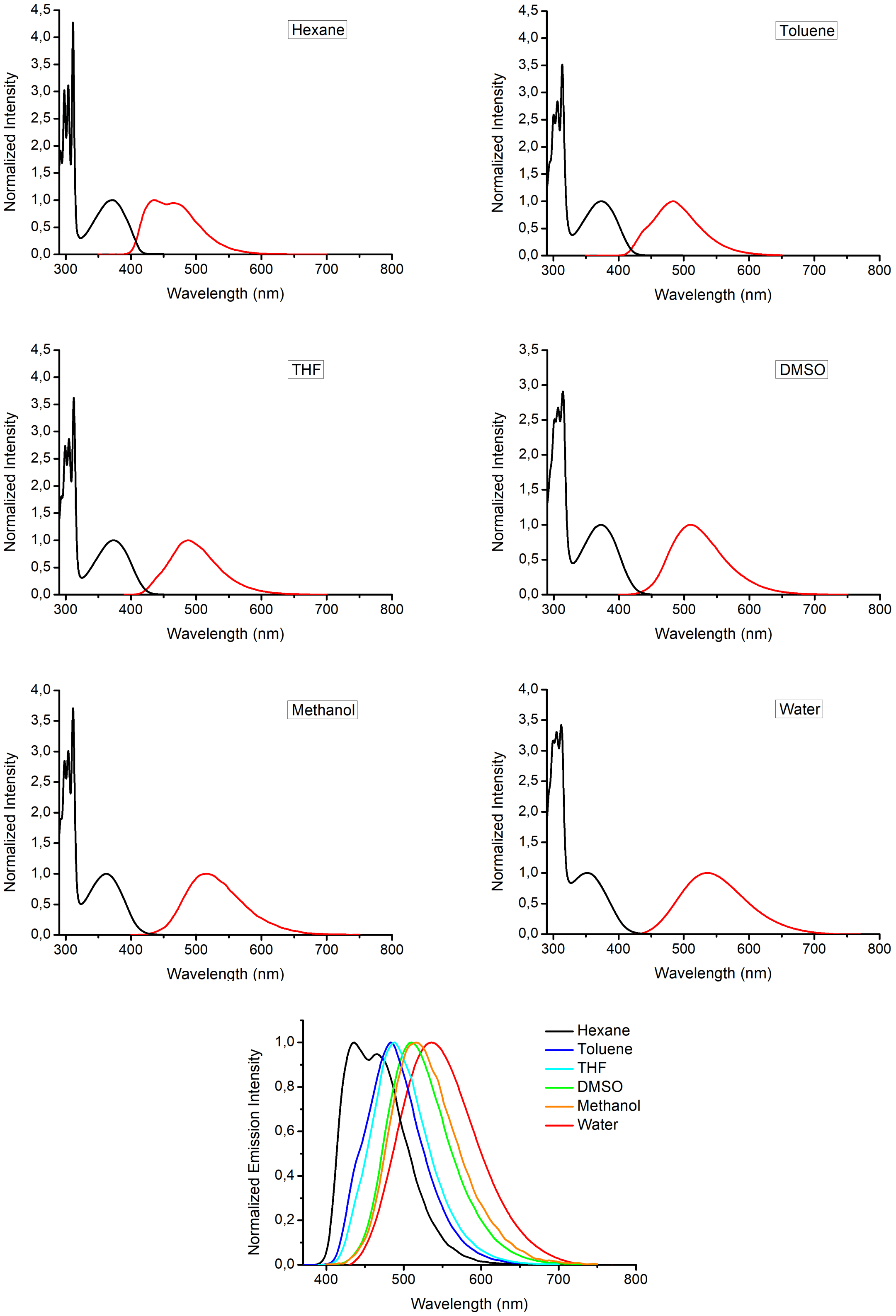

Figure S4. Normalized UV-vis absorption (black) and emission spectra (red) of **4** in solvents of different polarity. Bottom spectrum shows the solvatochromic effect on the emission.

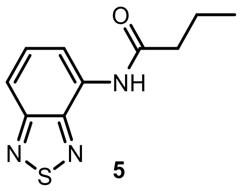

5

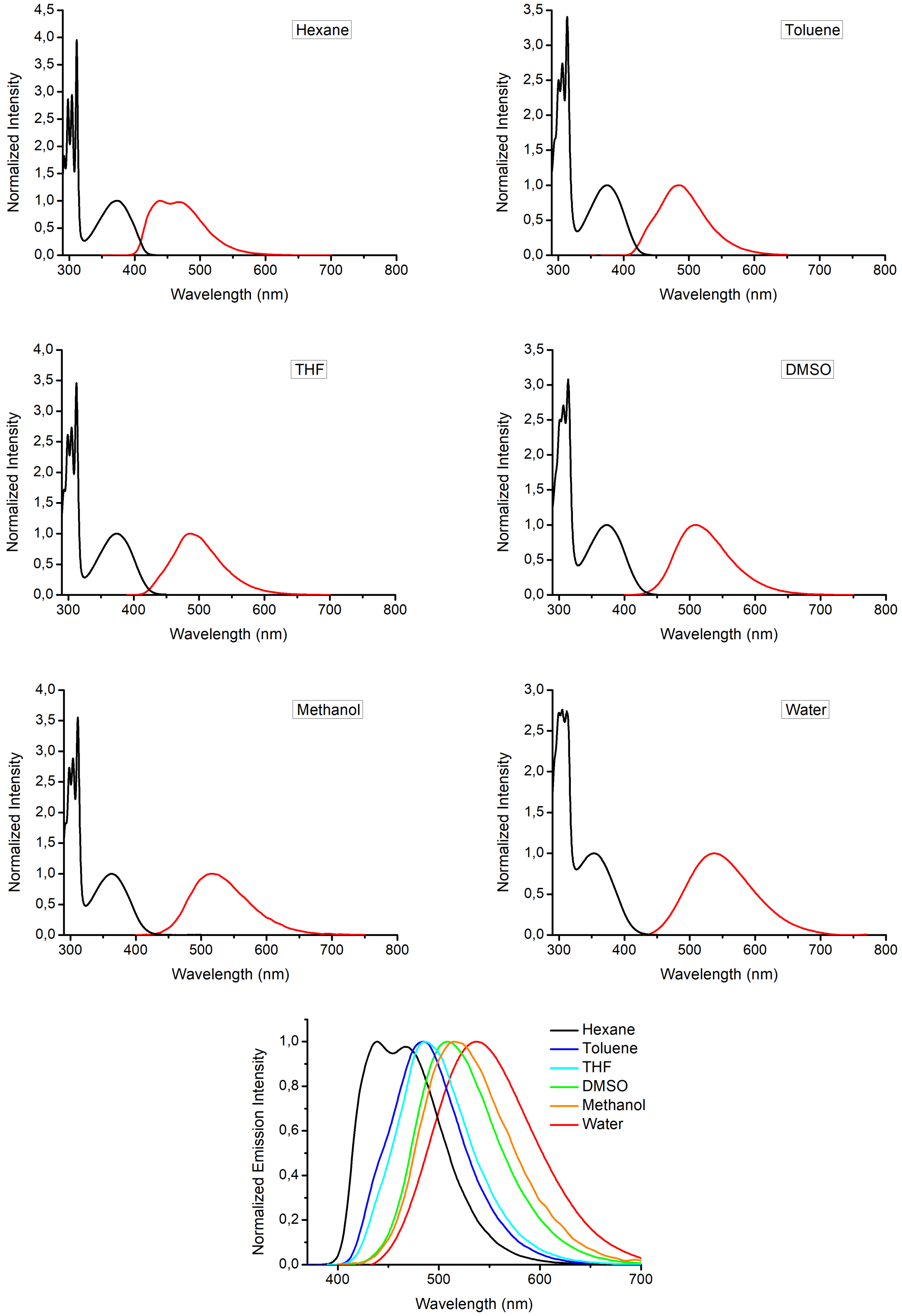

Figure S5. Normalized UV-vis absorption (black) and emission spectra (red) of **5** in solvents of different polarity. Bottom spectrum shows the solvatochromic effect on the emission.

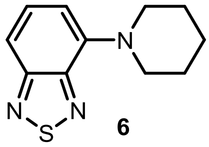

6

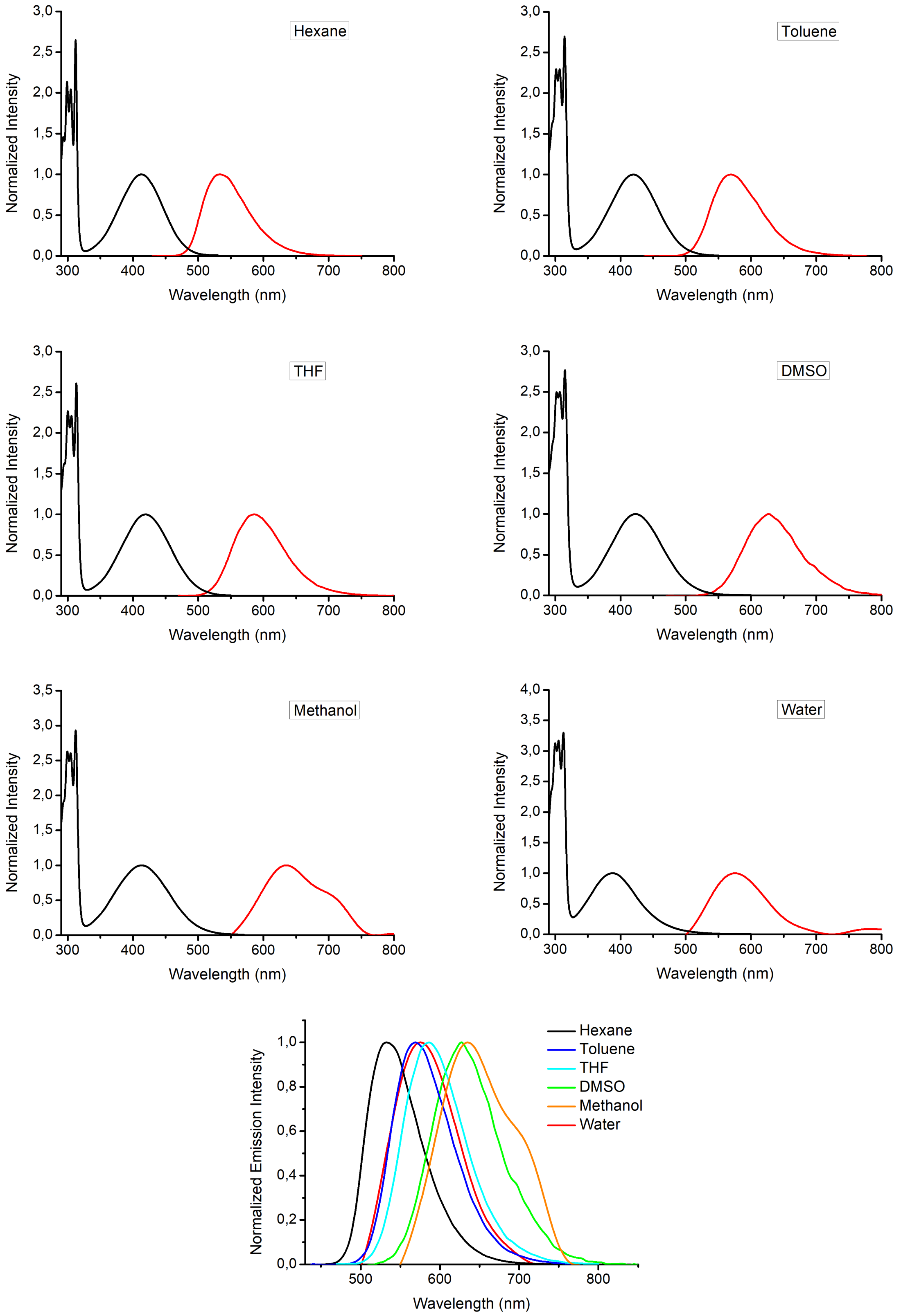

Figure S6. Normalized UV-vis absorption (black) and emission spectra (red) of **6** in solvents of different polarity. Bottom spectrum shows the solvatochromic effect on the emission. The observed emission in water likely originated from aggregates.

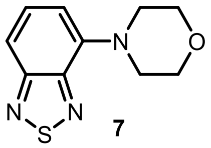

7

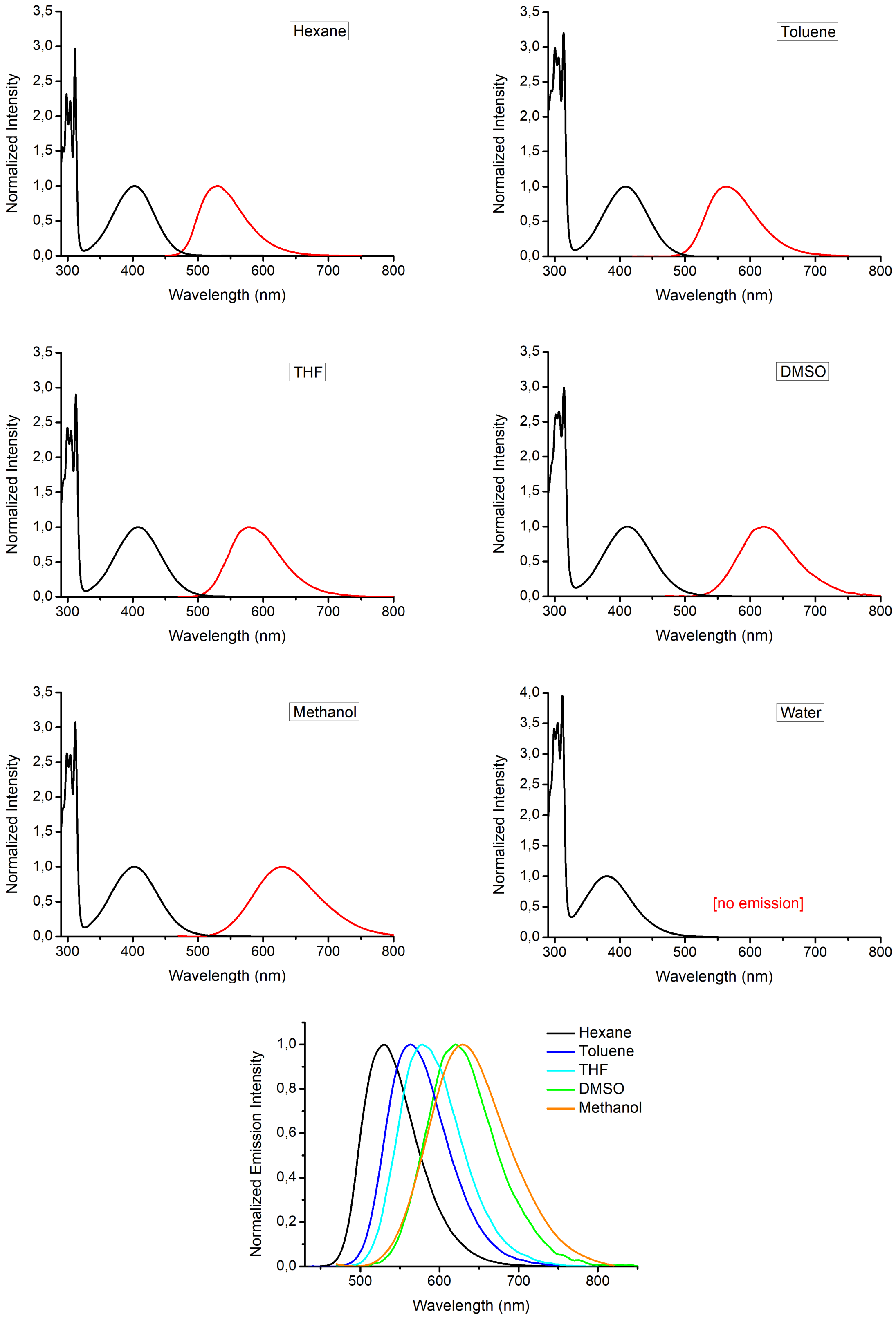

Figure S7. Normalized UV-vis absorption (black) and emission spectra (red) of **7** in solvents of different polarity. Bottom spectrum shows the solvatochromic effect on the emission.

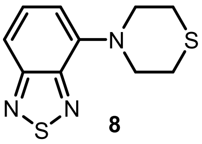

8

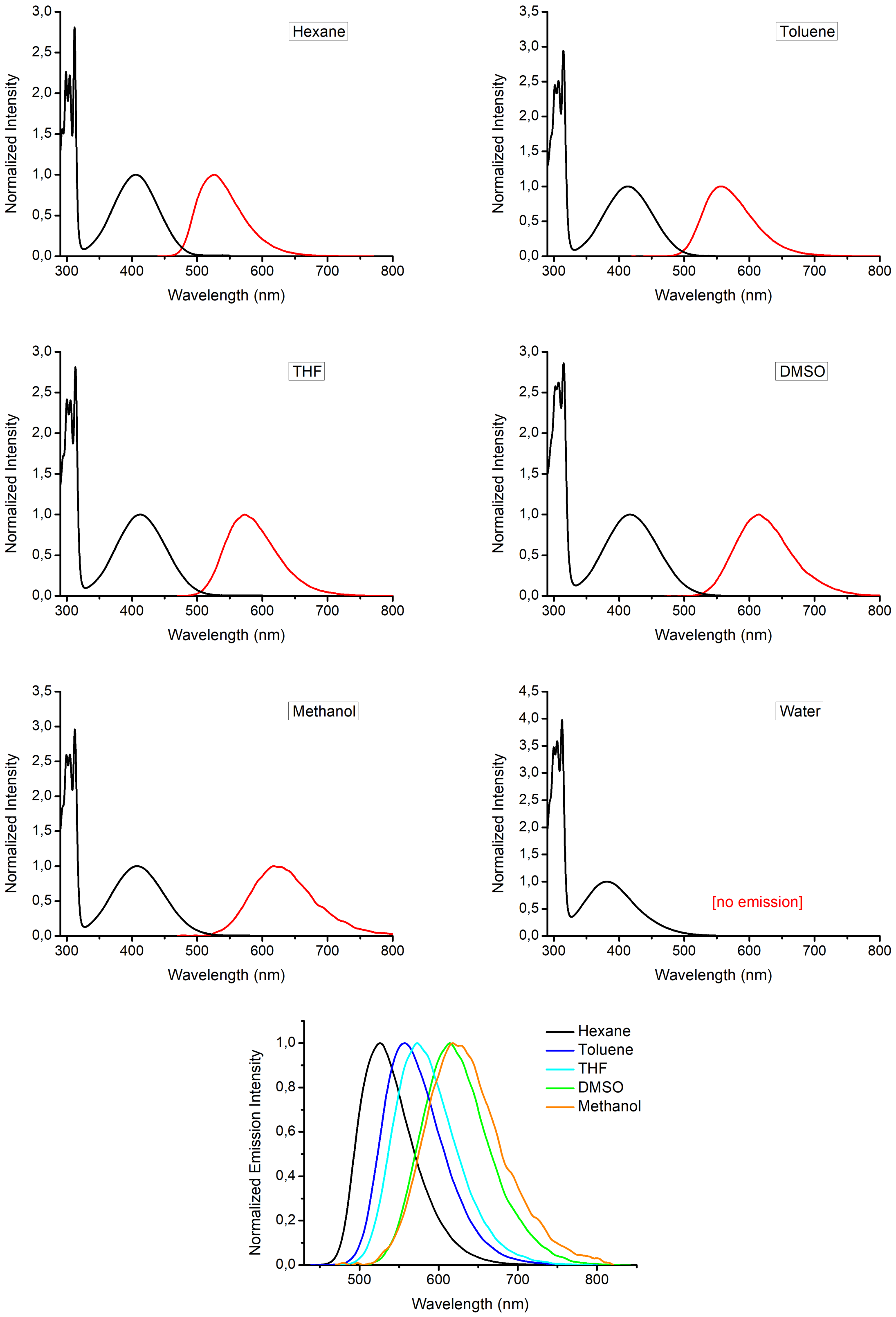

Figure S8. Normalized UV-vis absorption (black) and emission spectra (red) of **8** in solvents of different polarity. Bottom spectrum shows the solvatochromic effect on the emission.

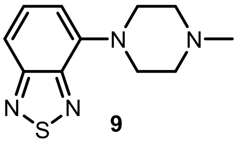

9

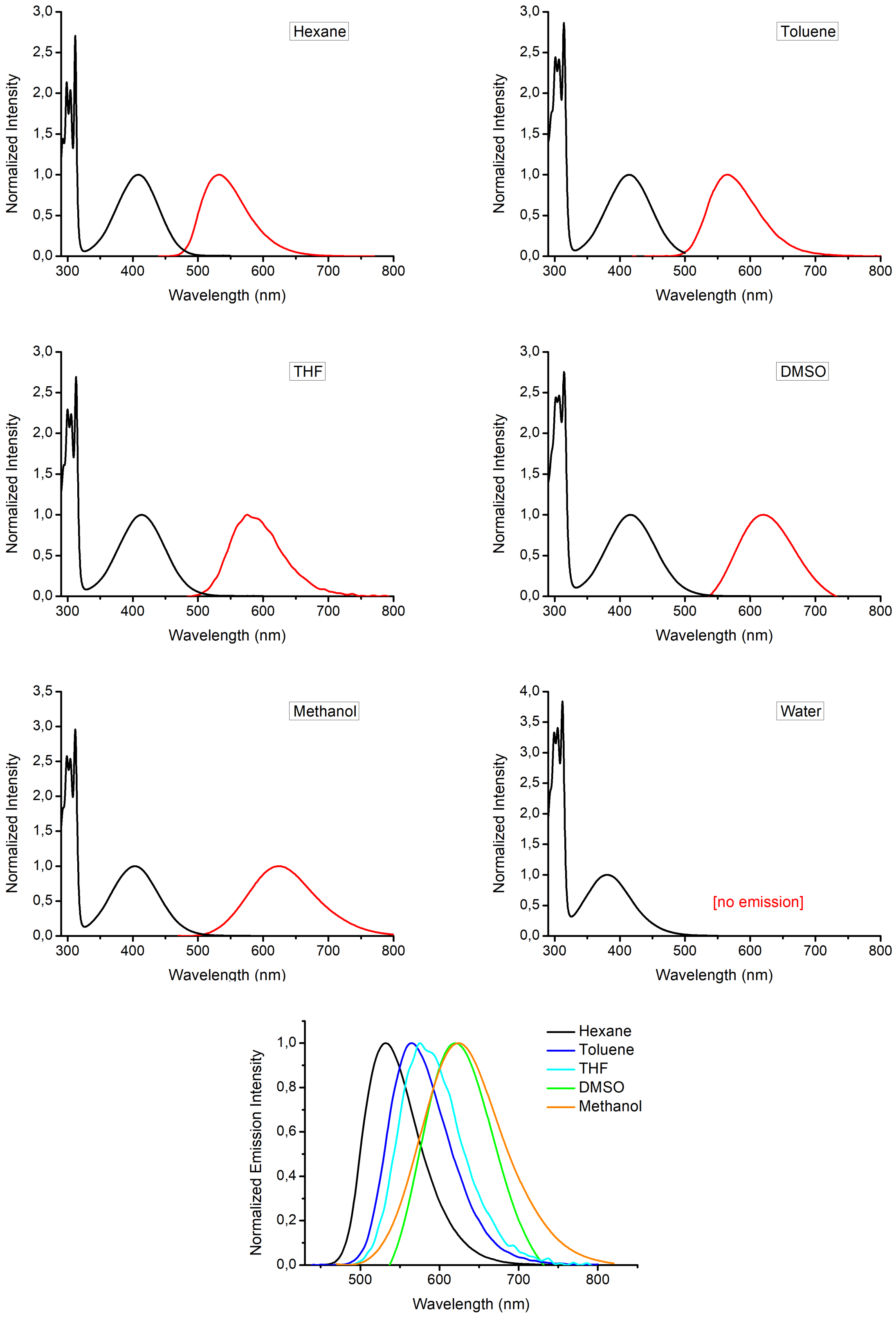

Figure S9. Normalized UV-vis absorption (black) and emission spectra (red) of **9** in solvents of different polarity. Bottom spectrum shows the solvatochromic effect on the emission.

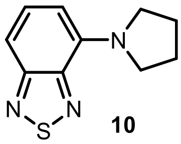

10

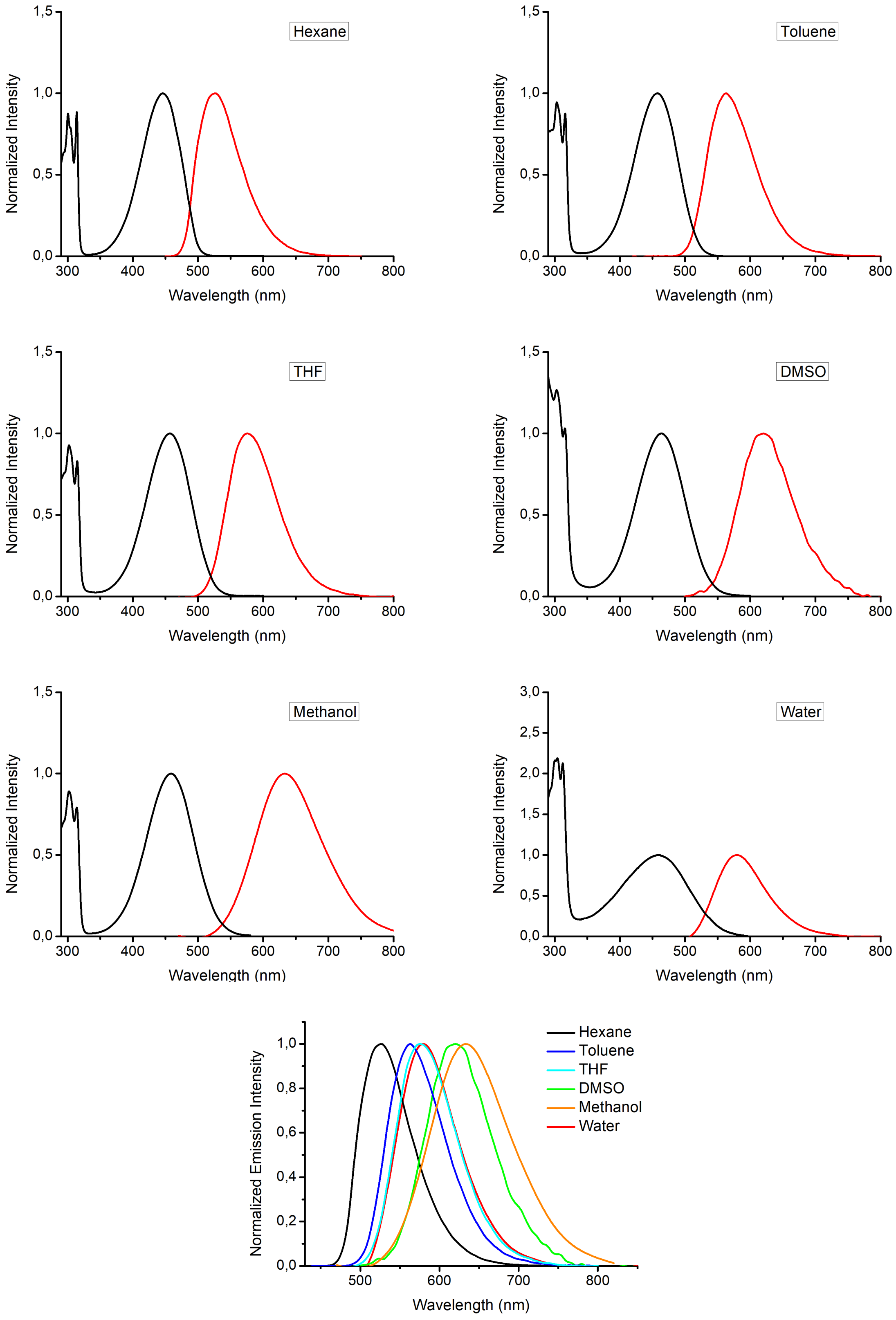

Figure S10. Normalized UV-vis absorption (black) and emission spectra (red) of **10** in solvents of different polarity. Bottom spectrum shows the solvatochromic effect on the emission. The observed emission in water likely originated from aggregates.

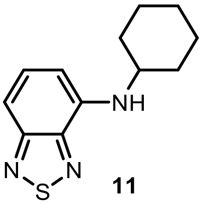

11

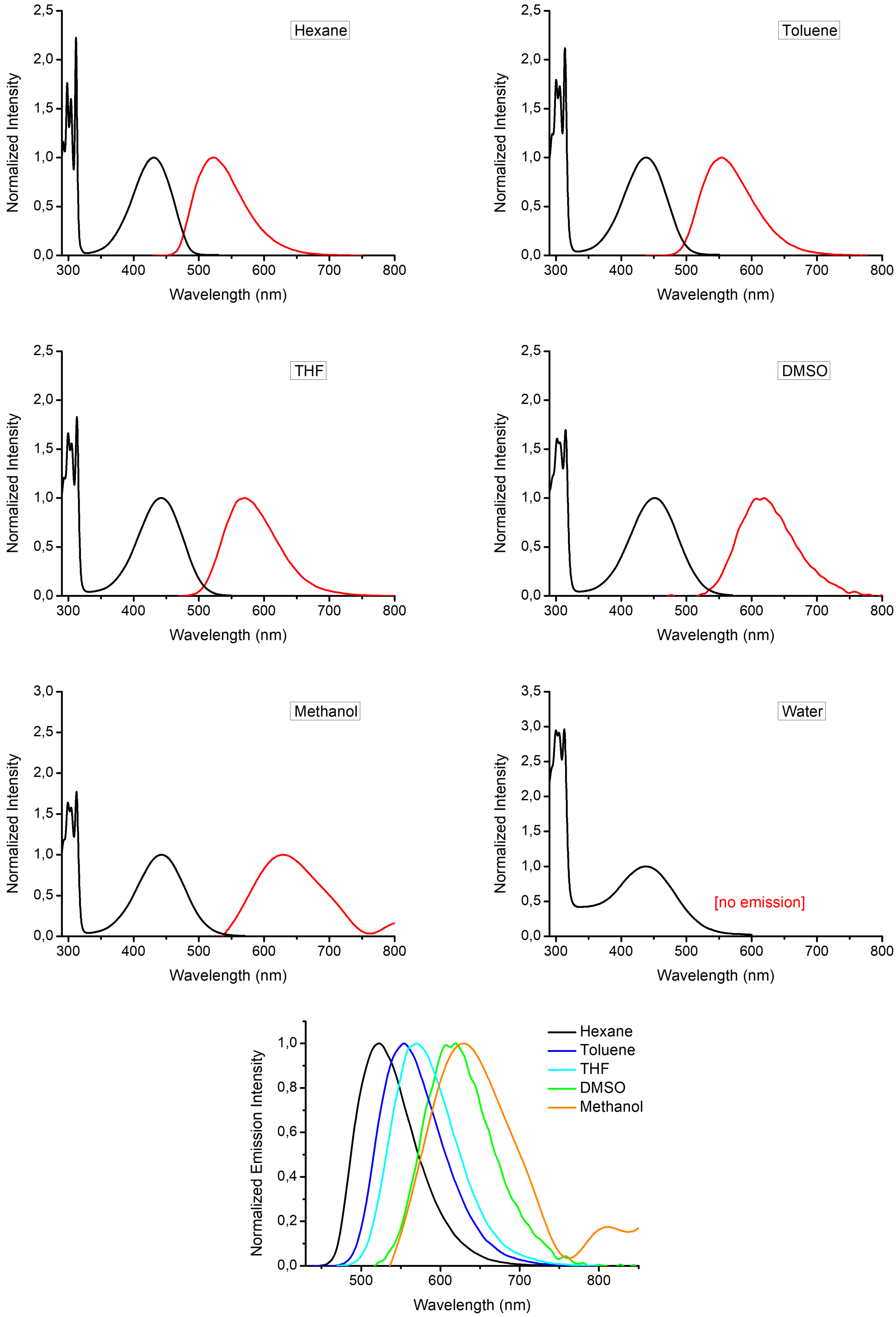

Figure S11. Normalized UV-vis absorption (black) and emission spectra (red) of **11** in solvents of different polarity. Bottom spectrum shows the solvatochromic effect on the emission.

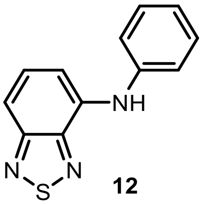

12

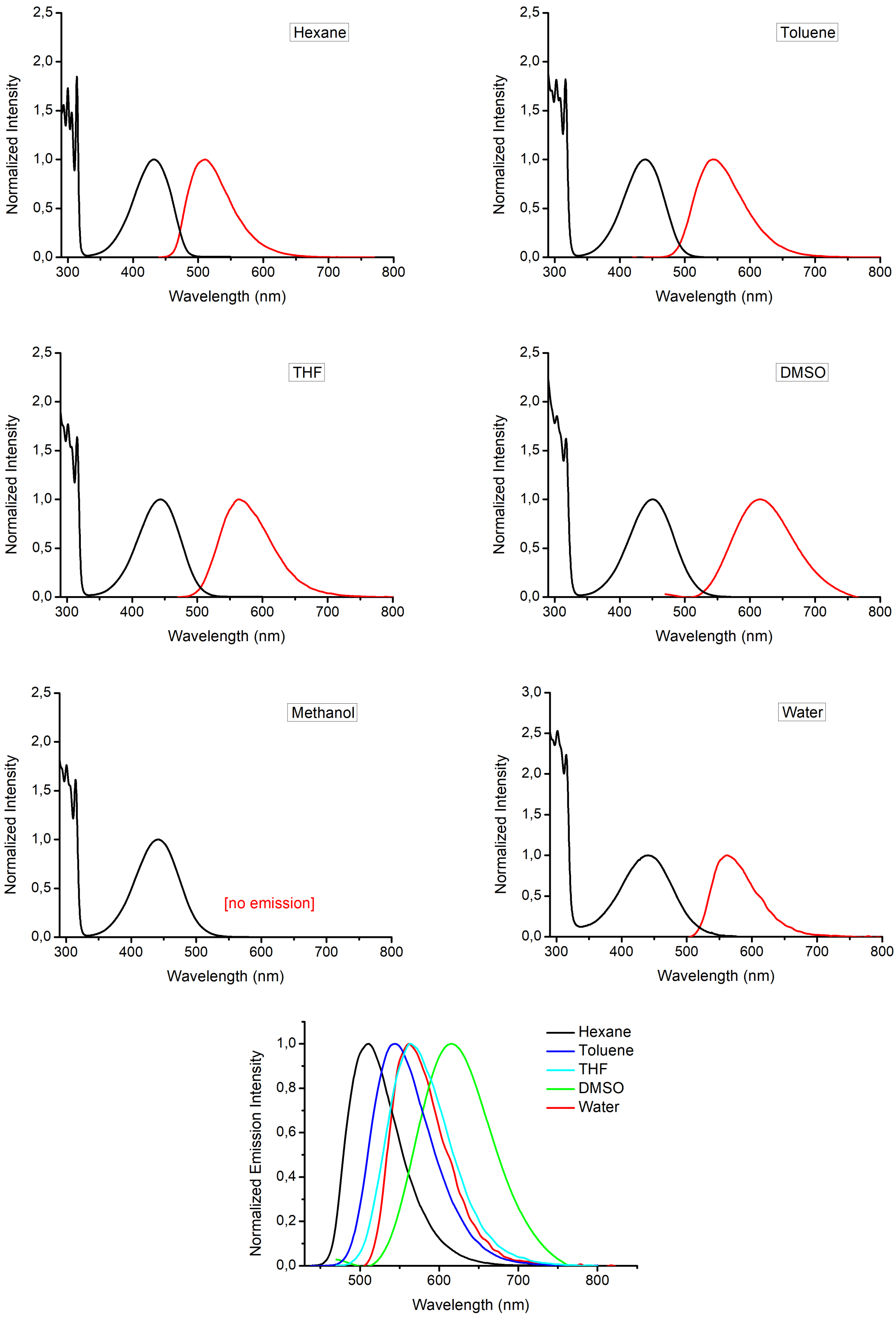

Figure S12. Normalized UV-vis absorption (black) and emission spectra (red) of **12** in solvents of different polarity. Bottom spectrum shows the solvatochromic effect on the emission. The observed emission in water likely originated from aggregates.

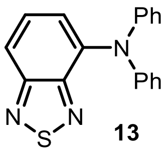

13

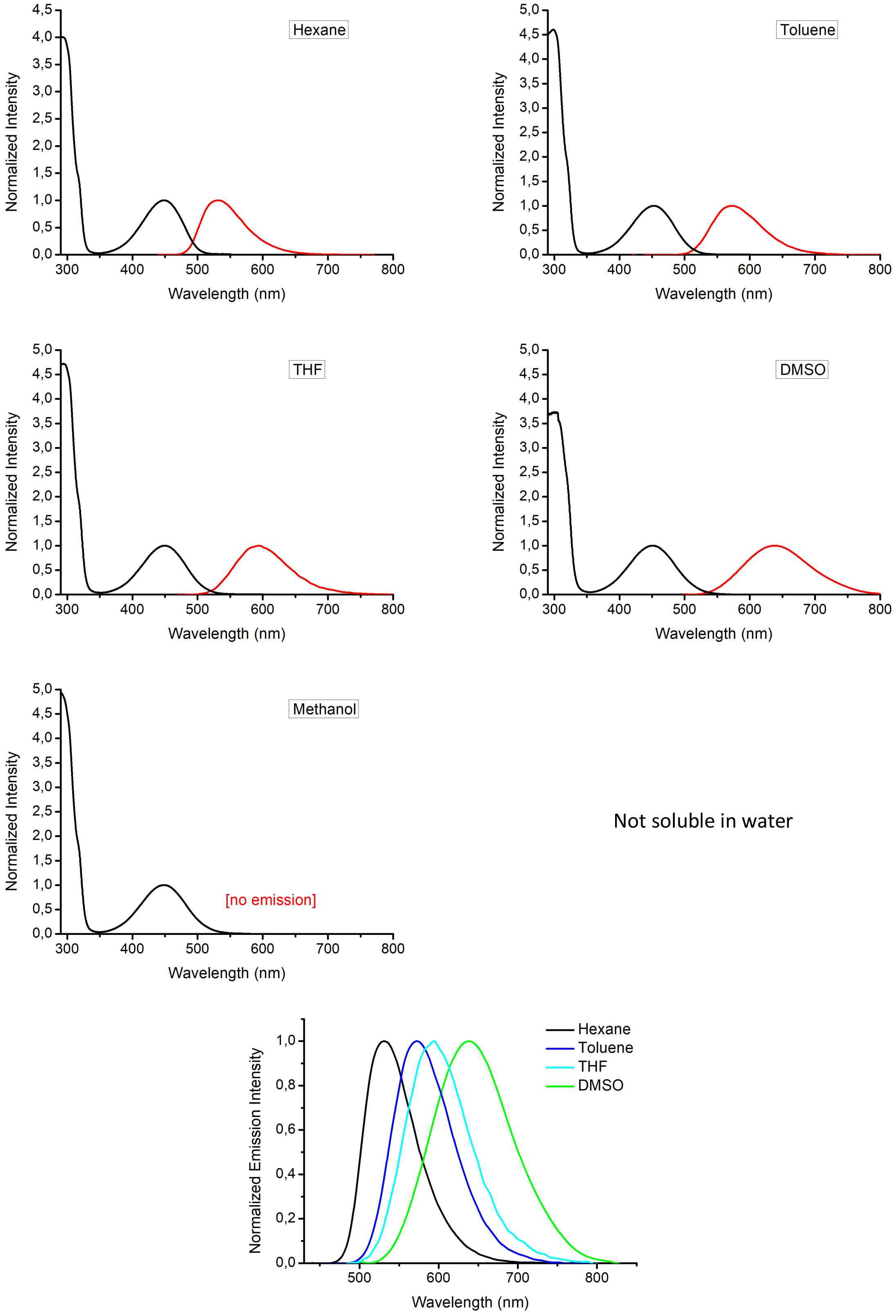

Figure S13. Normalized UV-vis absorption (black) and emission spectra (red) of **13** in solvents of different polarity. Bottom spectrum shows the solvatochromic effect on the emission.

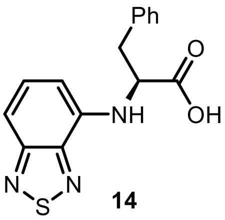

14

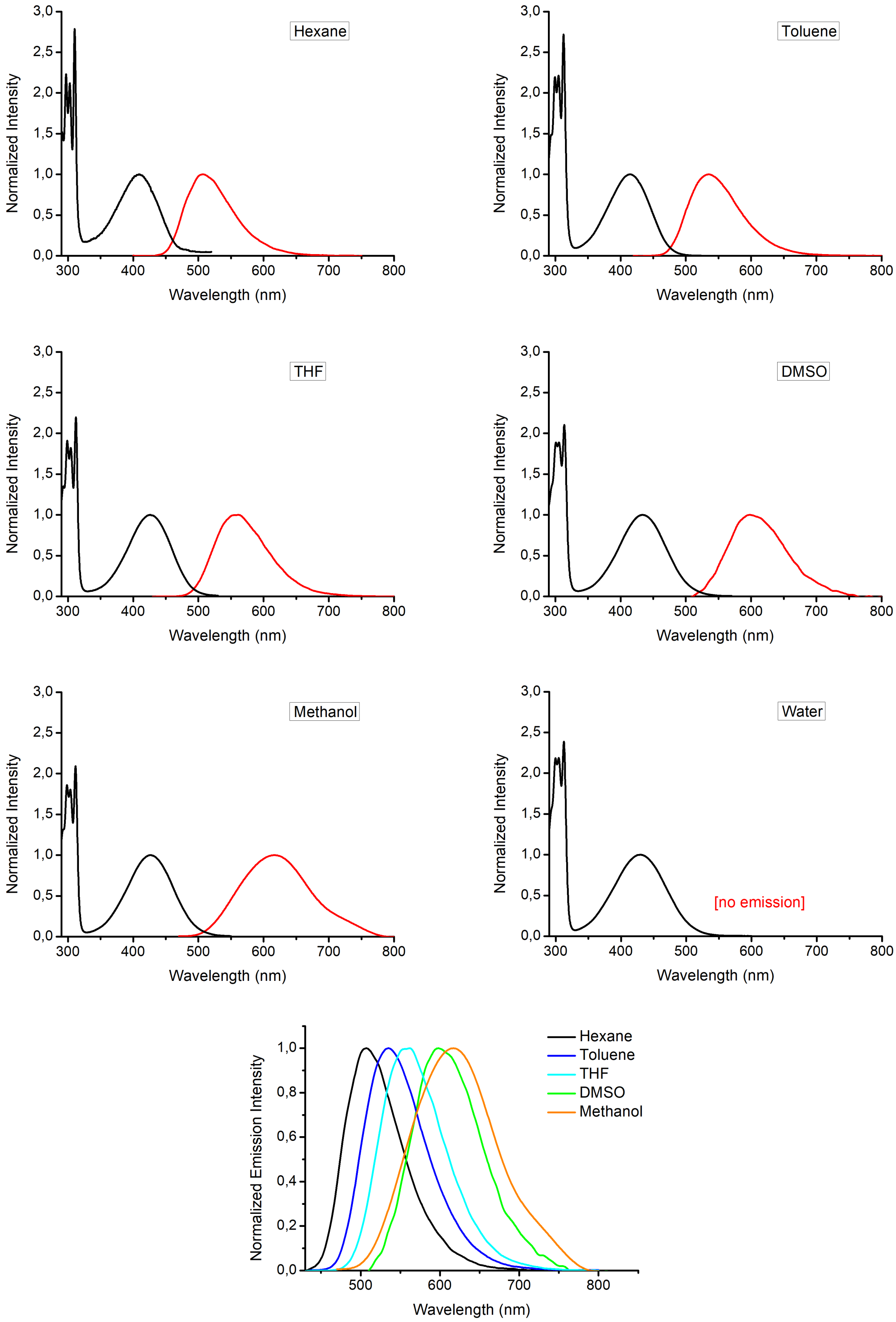

Figure S14. Normalized UV-vis absorption (black) and emission spectra (red) of **14** in solvents of different polarity. Bottom spectrum shows the solvatochromic effect on the emission.

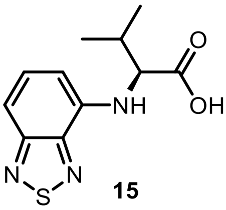

15

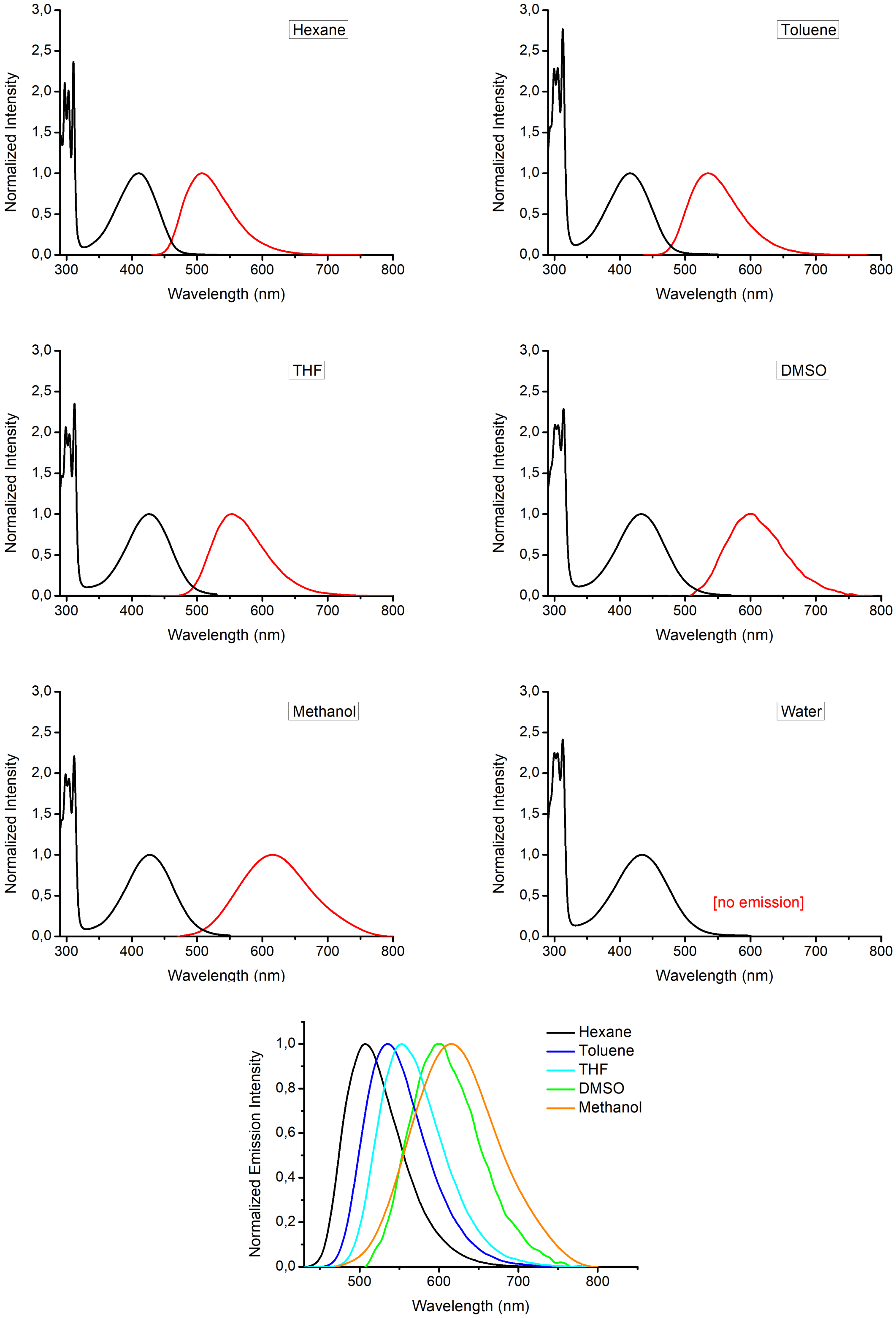

Figure S15. Normalized UV-vis absorption (black) and emission spectra (red) of **15** in solvents of different polarity. Bottom spectrum shows the solvatochromic effect on the emission.

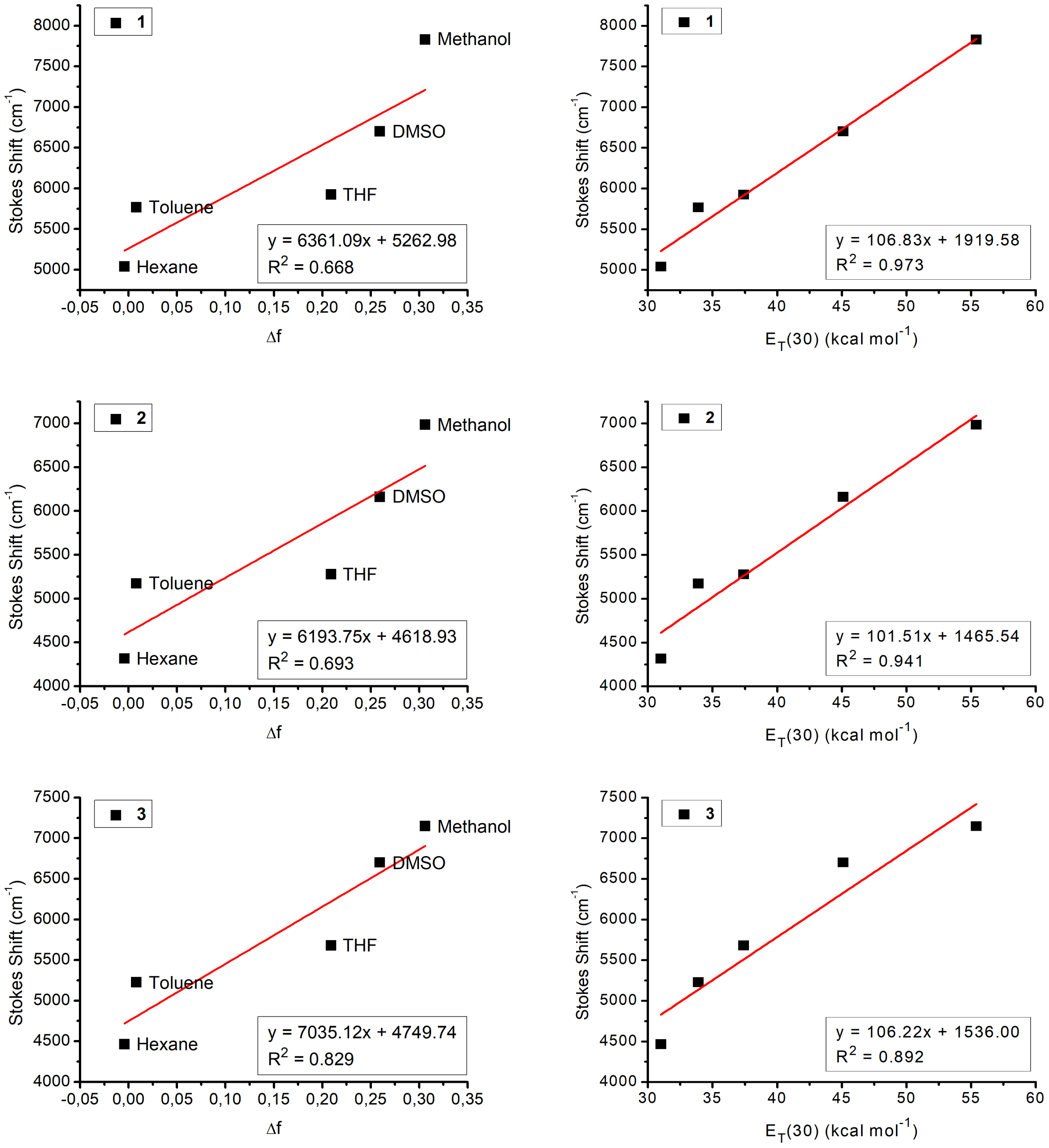

Figure S16. Lippert-Mataga plots (left) and correlation between Stokes shift and $E_T(30)$ solvent polarity parameter (right) for compounds **1-3**, with the latter showing better linearity.

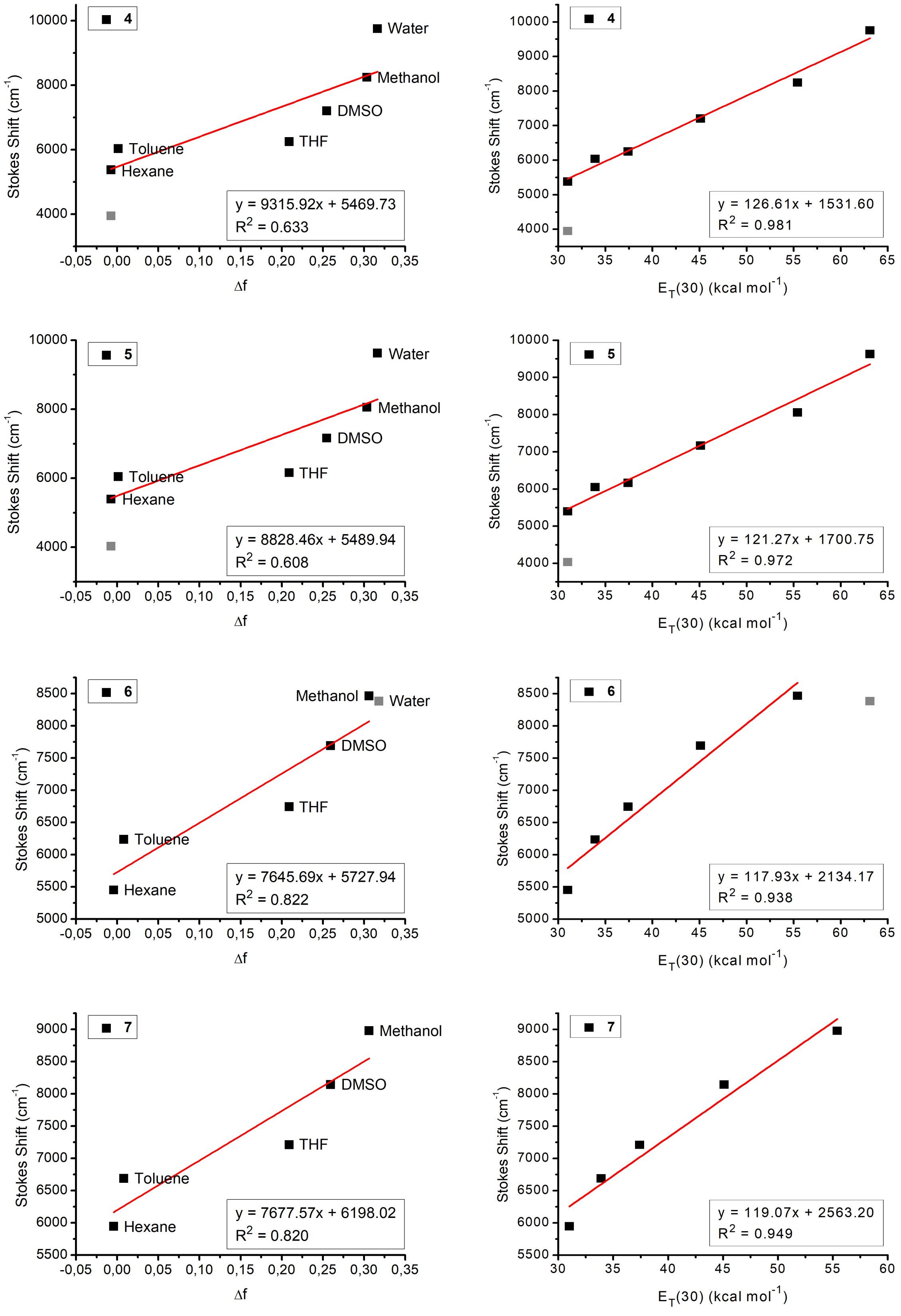

Figure S17. Lippert-Mataga plots (left) and correlation between Stokes shift and $E_T(30)$ solvent polarity parameter (right) for compounds **4-7**, with the latter showing better linearity. Grey data points were not considered for the linear fit.

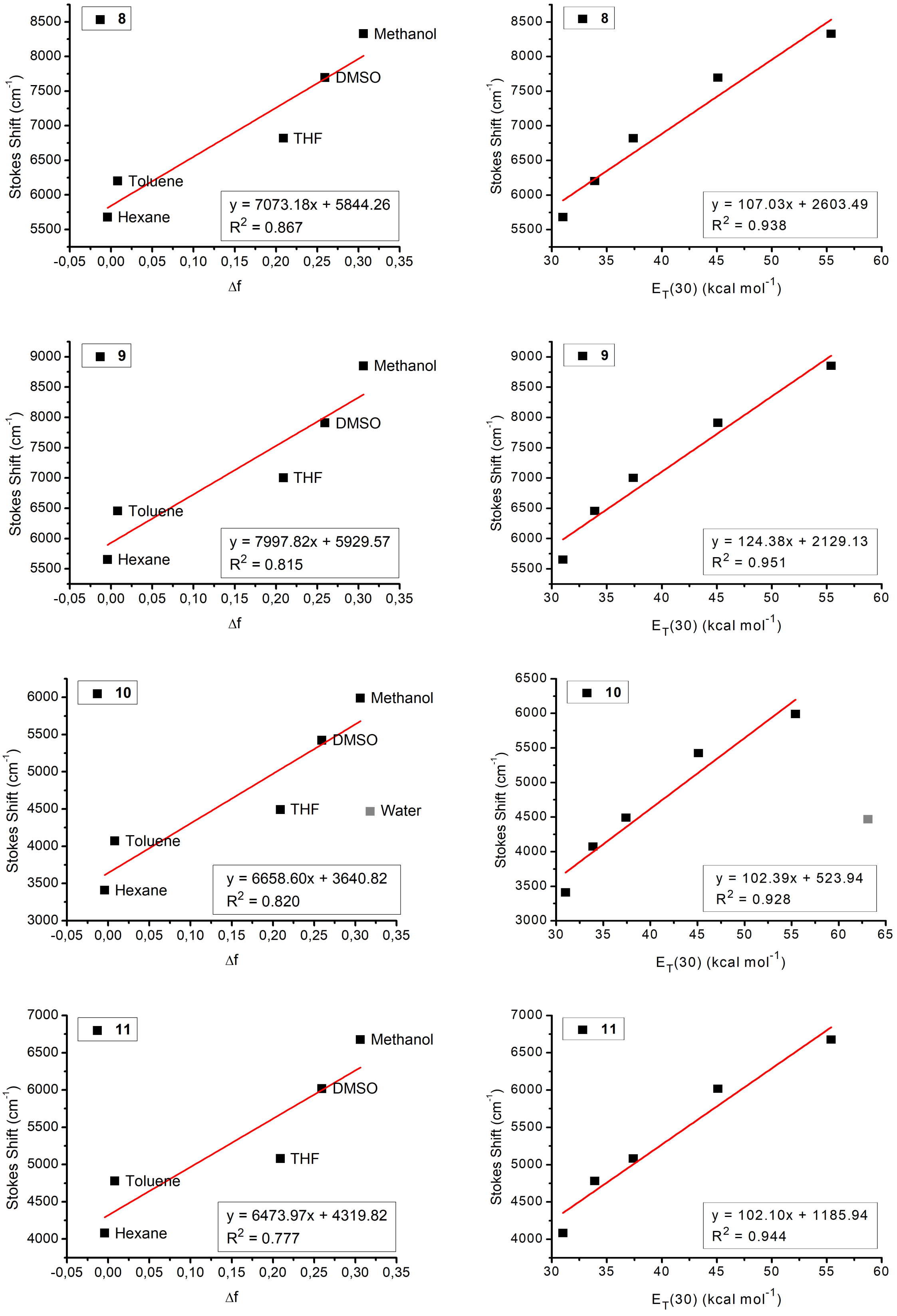

Figure S18. Lippert-Mataga plots (left) and correlation between Stokes shift and $E_T(30)$ solvent polarity parameter (right) for compounds **8-11**, with the latter showing better linearity. Grey data points were not considered for the linear fit.

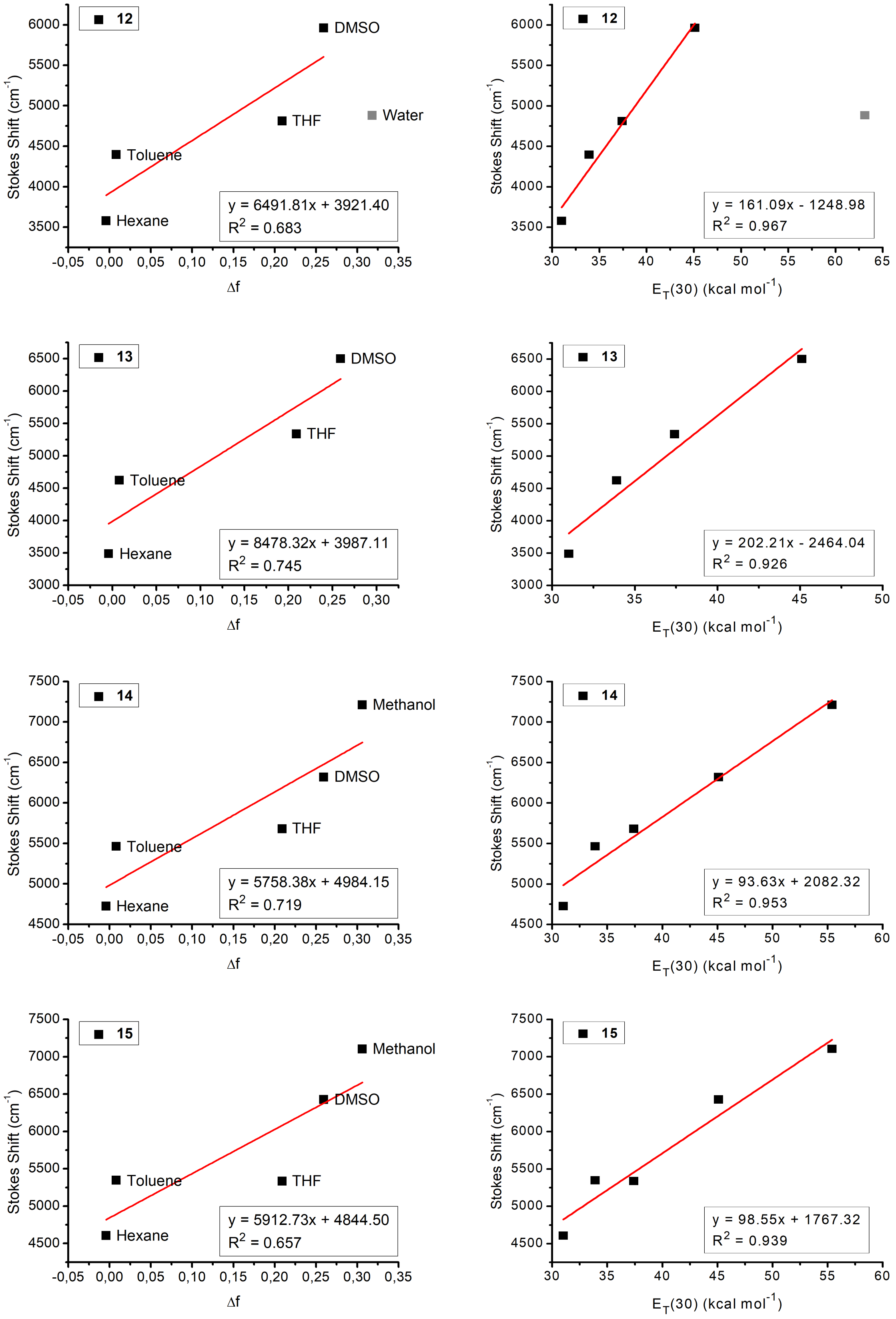

Figure S19. Lippert-Mataga plots (left) and correlation between Stokes shift and $E_T(30)$ solvent polarity parameter (right) for compounds **12-15**, with the latter showing better linearity. Grey data points were not considered for the linear fit.

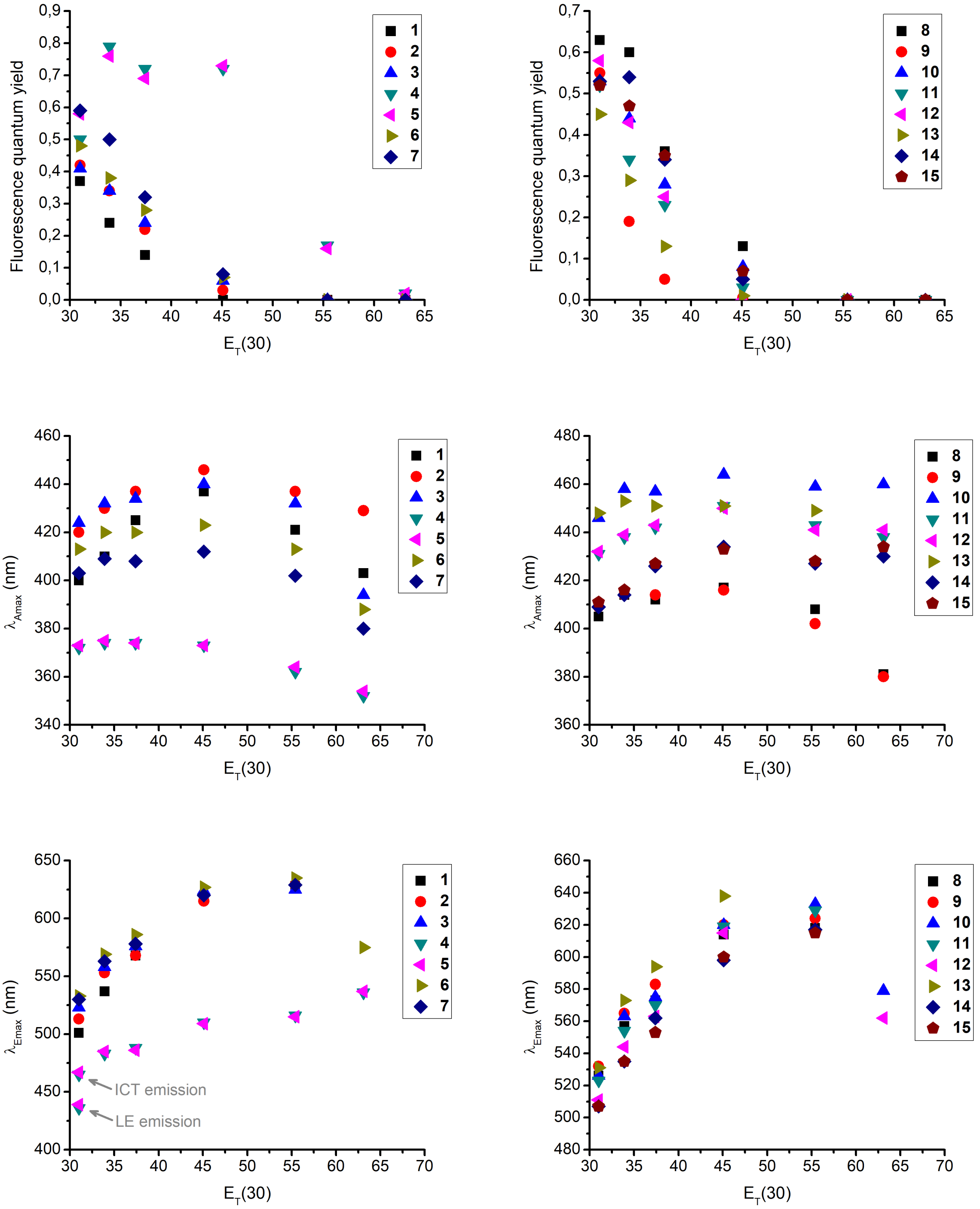

Figure S20. Correlations of fluorescence quantum yield (top), maximum absorption wavelength λ_{Amax} (middle) and maximum emission wavelength λ_{Emax} (bottom) *vs* $E_T(30)$ solvent polarity parameter.

A.

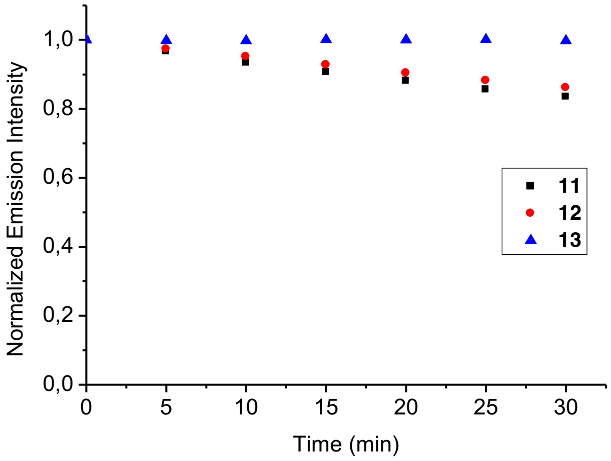

B.

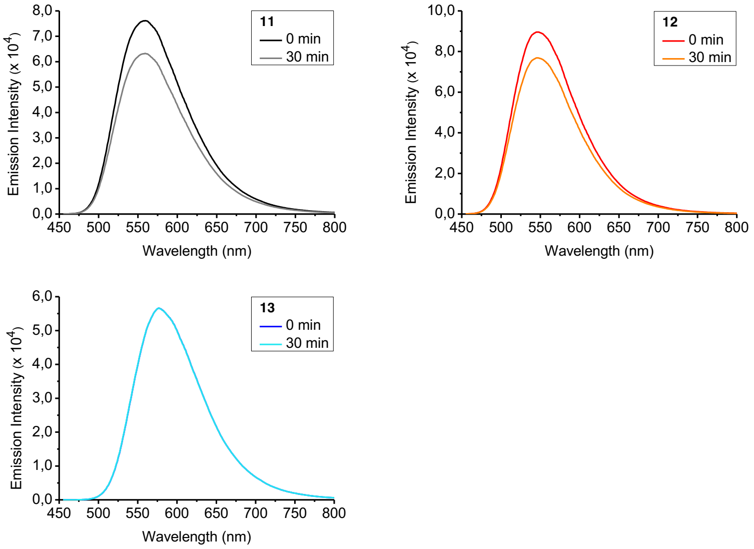

Figure S21. A) Photostability of compounds **11-13** in toluene during irradiation (λ_{ex} = 445 nm) for 30 min using the Shimadzu RF-6000 spectrofluorophotometer (ex. bandwidth = 20 nm). Each solution was prepared to give an absorbance value at 0.10 (λ_{abs} = 445 nm). B) Emission spectra (λ_{ex} = 445 nm), before and after 30 min irradiation. λ_{em} = 554 (**11**), 544 (**12**) or 573 nm (**13**).

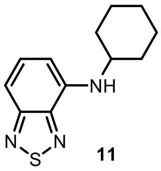

11

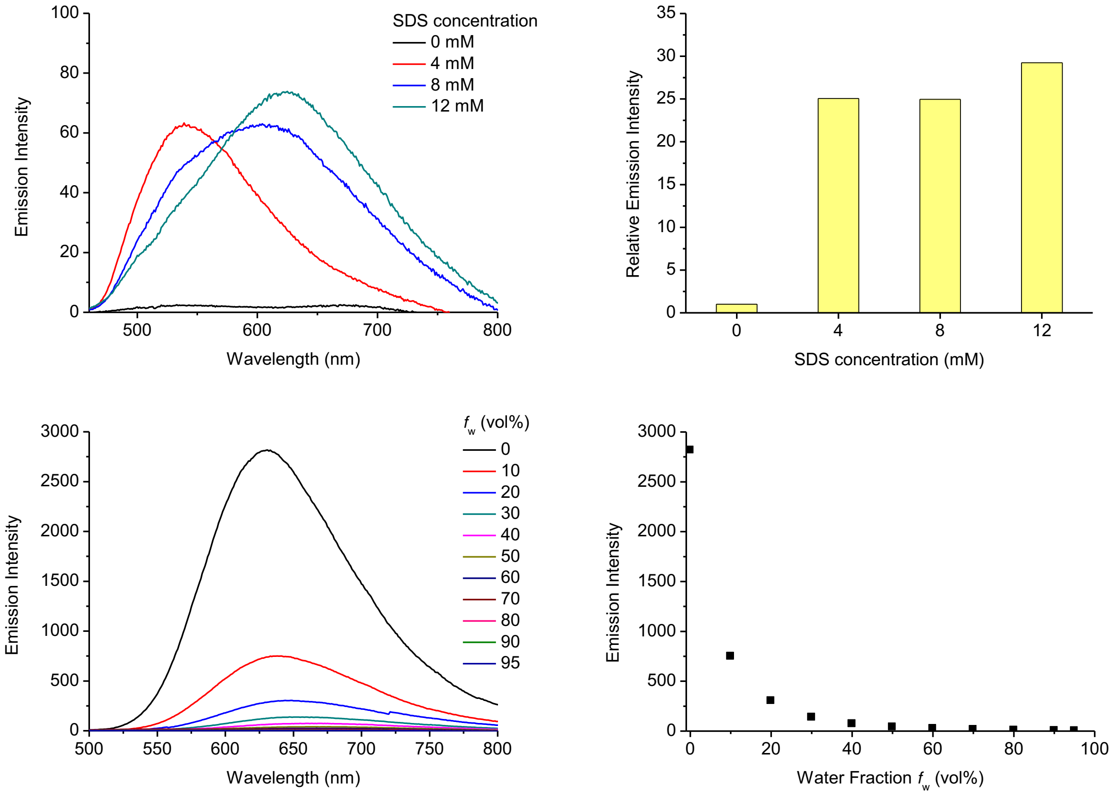

Figure S22. Top row: Emission spectra (left) (λ_{ex} = 438 nm) and corresponding relative emission intensities (right) of **11** (10 µM) in water/DMSO 95:5 in the presence of different concentrations of sodium dodecyl sulfate (SDS). Bottom row: Emission spectra (left; λ_{ex} = 451 nm) and corresponding emission intensities (right) of **11** (10 µM) in mixtures of DMSO/water with varying water fraction (f_w).

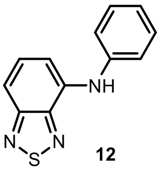

12

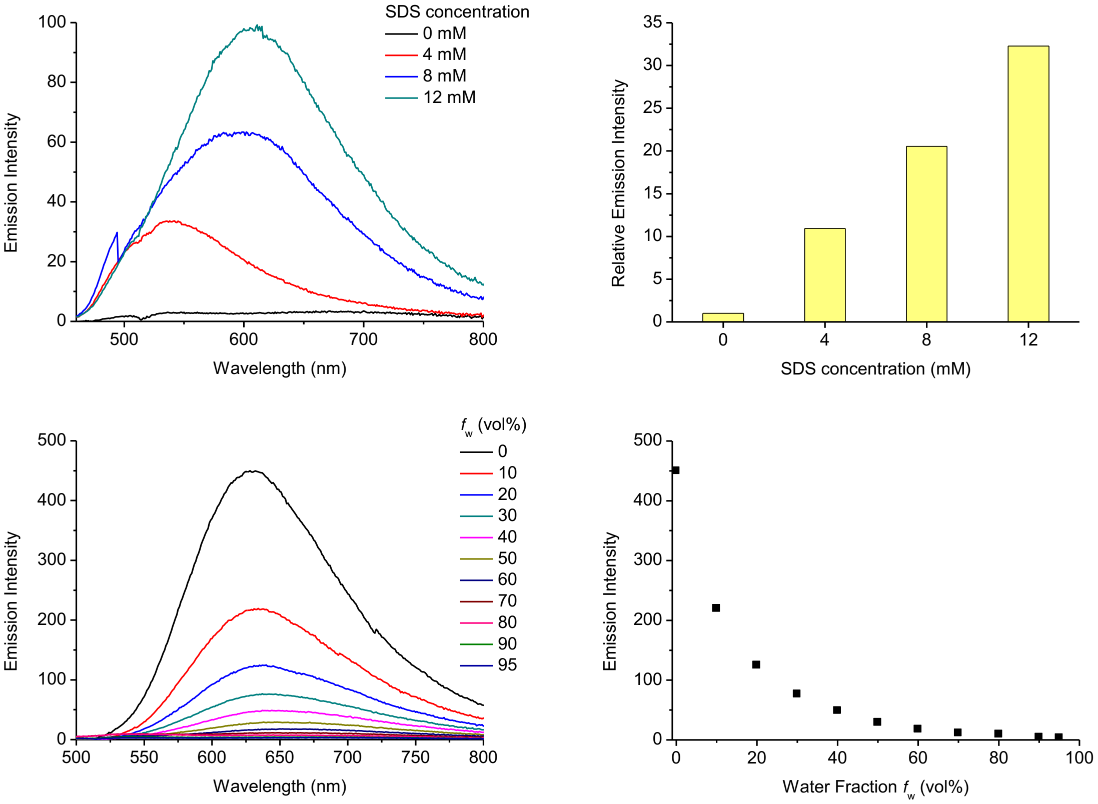

Figure S23. Top row: Emission spectra (left) (λ_{ex} = 441 nm) and corresponding relative emission intensities (right) of **12** (10 μM) in water/DMSO 95:5 in the presence of different concentrations of sodium dodecyl sulfate (SDS). Bottom row: Emission spectra (left) (λ_{ex} = 441 nm) and corresponding emission intensities (right) of **12** (10 μM) in mixtures of DMSO/water with varying water fraction (f_w).

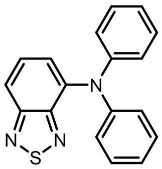

13

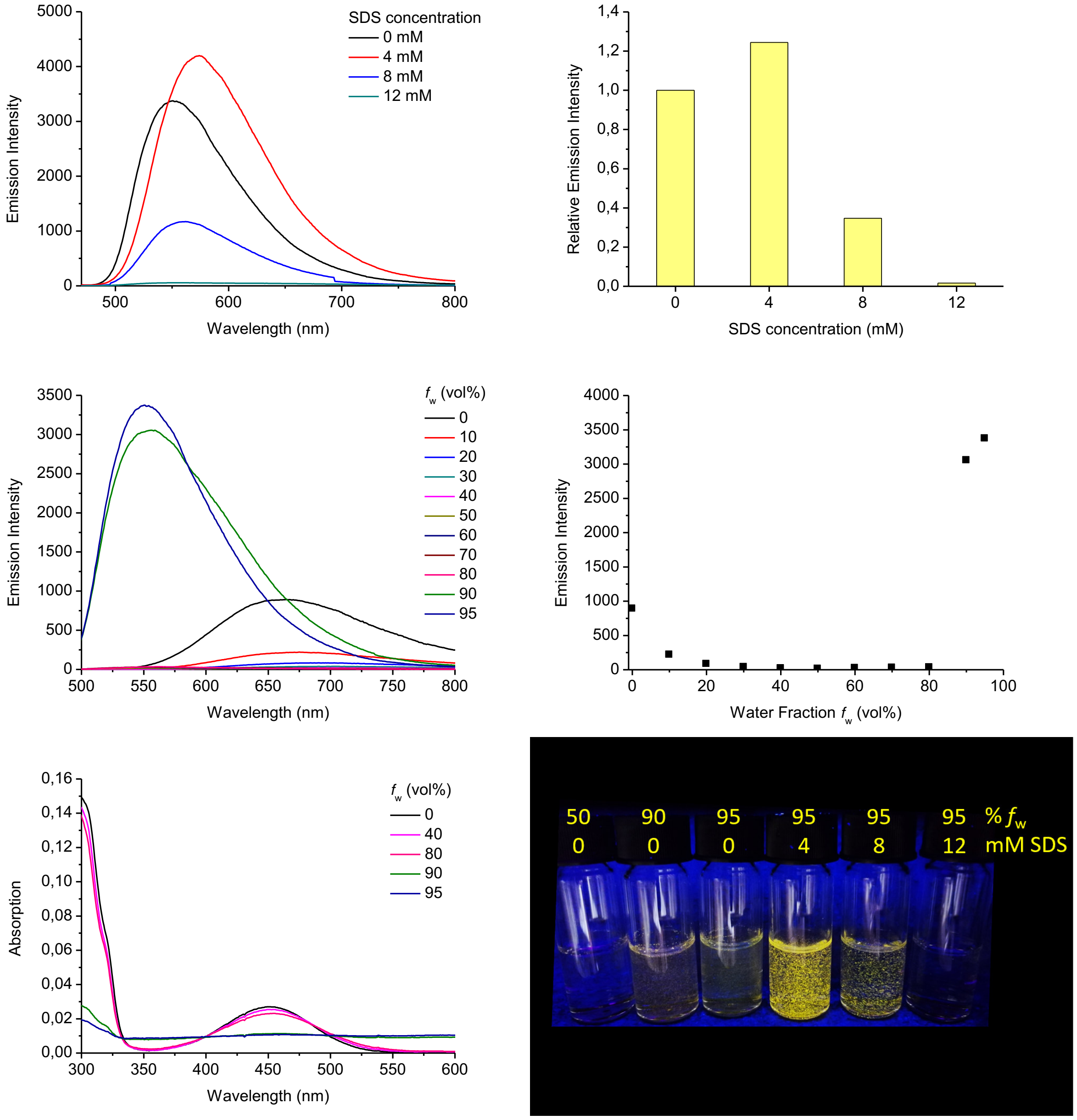

Figure S24. Top row: Emission spectra (left) (λ_{ex} = 451 nm) and corresponding relative emission intensities (right) of **13** (10 µM) in water/DMSO 95:5 in the presence of different concentrations of sodium dodecyl sulfate (SDS). Middle row: Emission spectra (left) (λ_{ex} = 451 nm) and corresponding emission intensities (right) of **13** (10 µM) in mixtures of DMSO/water with varying water fraction (f_w). Bottom left: Corresponding absorption spectra in selected DMSO/water mixtures, indicating aggregation effects at f_w ≥90%. Bottom right: Picture of selected samples of **13** (10 µM) exposed to UV light (λ = 365 nm) showing weak aggregation-induced emission at high f_w and the effect of SDS concentration on aggregation and fluorescence.

IV. Lipophilicity (logP) Data

Table S1. Experimental and calculated logP values for compounds **1-15** and **LD-BTD1**[27].

Compd.	Exp. logP	Calcd. logP[12]
**1**	1.7	1.5
**2**	2.4	1.8
**3**	2.4	2.1
**4**	1.4	1.2
**5**	2.3	2.6
**6**	n.d.	3.0
**7**	2.0	1.9
**8**	n.d.	2.5
**9**	1.5	2.0
**10**	n.d.	2.5
**11**	n.d.	4.4
**12**	n.d.	4.0
**13**	n.d.	5.5
**14**	1.5	2.1
**15**	1.5	1.4
**LD-BTD1**	3.3[27]	3.9

n.d. = not determined due to poor water solubility.

V. Computational Results

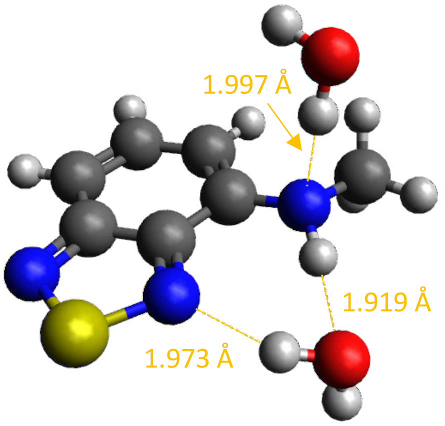

Figure S25. Optimized ground-state geometry of compound **2**. Hydrogen bonds and their lengths are indicated in yellow.

Table S2. Calculated absorption (λ_{Amax}) and emission (λ_{Emax}) in three different solvents in comparison with experimental data.

Solvent	Compd.	λ_{Amax} (nm / eV)		λ_{Emax} (nm / eV)	
		Exp.	Calcd.	Exp.	Calcd.
hexane	1	400 / 3.10	393 / 3.15	501 / 2.47	519 / 2.39
THF		425 / 2.92	422 / 2.94	568 / 2.18	674 / 1.84
H_2O		403 / 3.08	394 / 3.15	-	-
hexane	2	420 / 2.95	411 / 3.01	513 / 2.42	523 / 2.37
THF		437 / 2.84	430 / 2.88	568 / 2.18	638 / 1.94
H_2O		429 / 2.89	397 / 3.12	-	-
hexane	3	424 / 2.92	400 / 3.10	523 / 2.37	527 / 2.35
THF		434 / 2.86	412 / 3.01	576 / 2.15	645 / 1.92
H_2O		394 / 3.15	377 / 3.28	-	-
hexane	4	372 / 3.33	358 / 3.46	436;465 / 2.84;2.67	456 / 2.72
THF		374 / 3.32	349 / 3.55	488 / 2.54	510 / 2.43
H_2O		352 / 3.52	345 / 3.59	536 / 2.31	547 / 2.27

Table S3. Natural transition orbitals for compounds **1-4**.

**Compound**	**Hole NTO**		**Particle NTO**
**1**	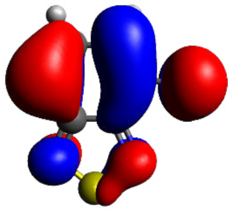	0.997 →	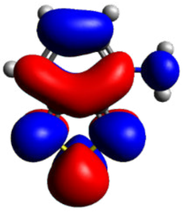
**2**	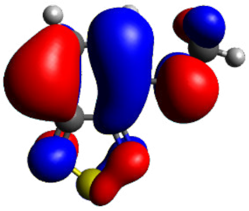	0.998 →	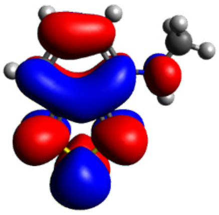
**3**	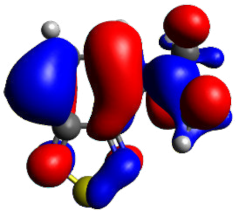	0.996 →	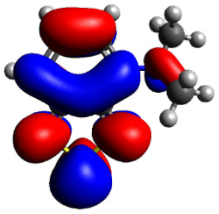
**4**	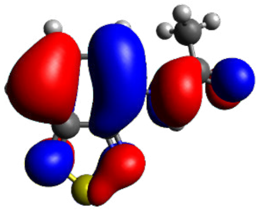	0.994 →	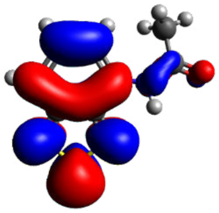

NR'R'' = NH_2 (**1**), NHMe (**2**), NMe_2 (**3**), NHAc (**4**)
Dihedral angles: φ = (5,4,N,R''), ω = (5,4,N,R')

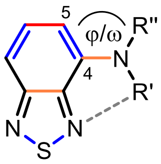

Most significant bond length changes
upon excitation from S0 to S1:
3-5% elongation
3-4% shortening
1-3% shortening
4-6% shortening of H-bond R'---N (**1**, **2**, **4**)

	**Dihedral angles (/**	
	**S0**	**S1**
**1**	-21 / -164	0 / -180
**2**	-7 / -172	0 / -180
**3**	-6 / 137	-7 / 167
**4**	-14 / 171	0 / -180

Figure S26. Calculated changes in bond lengths and dihedral angles of **1-4** upon excitation from the ground state (S0) to the first excited state (S1) based on optimized S0 and S1 structures in hexane.

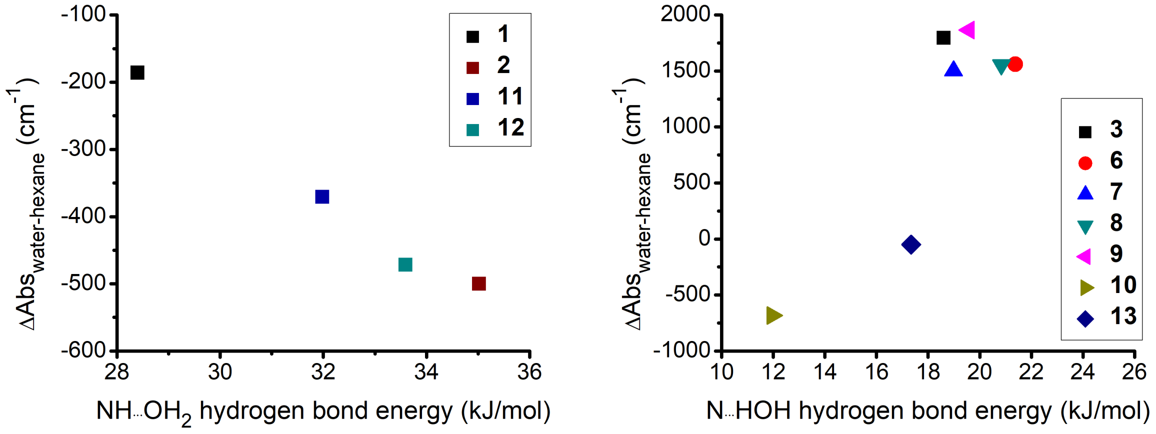

Figure S27. Correlations of hydrogen bond energy (in kJ/mol) and shift of absorption in water compared to hexane (in cm^{-1}). Left: linear red-shift of absorption with increasing hydrogen bond donor strength of the primary (**1**) or secondary (**2**, **11**, **12**) amine. Right: blue-shift of absorption with increasing hydrogen bond acceptor strength of the tertiary amine.

VI. Fluorescence Cell Imaging Pictures

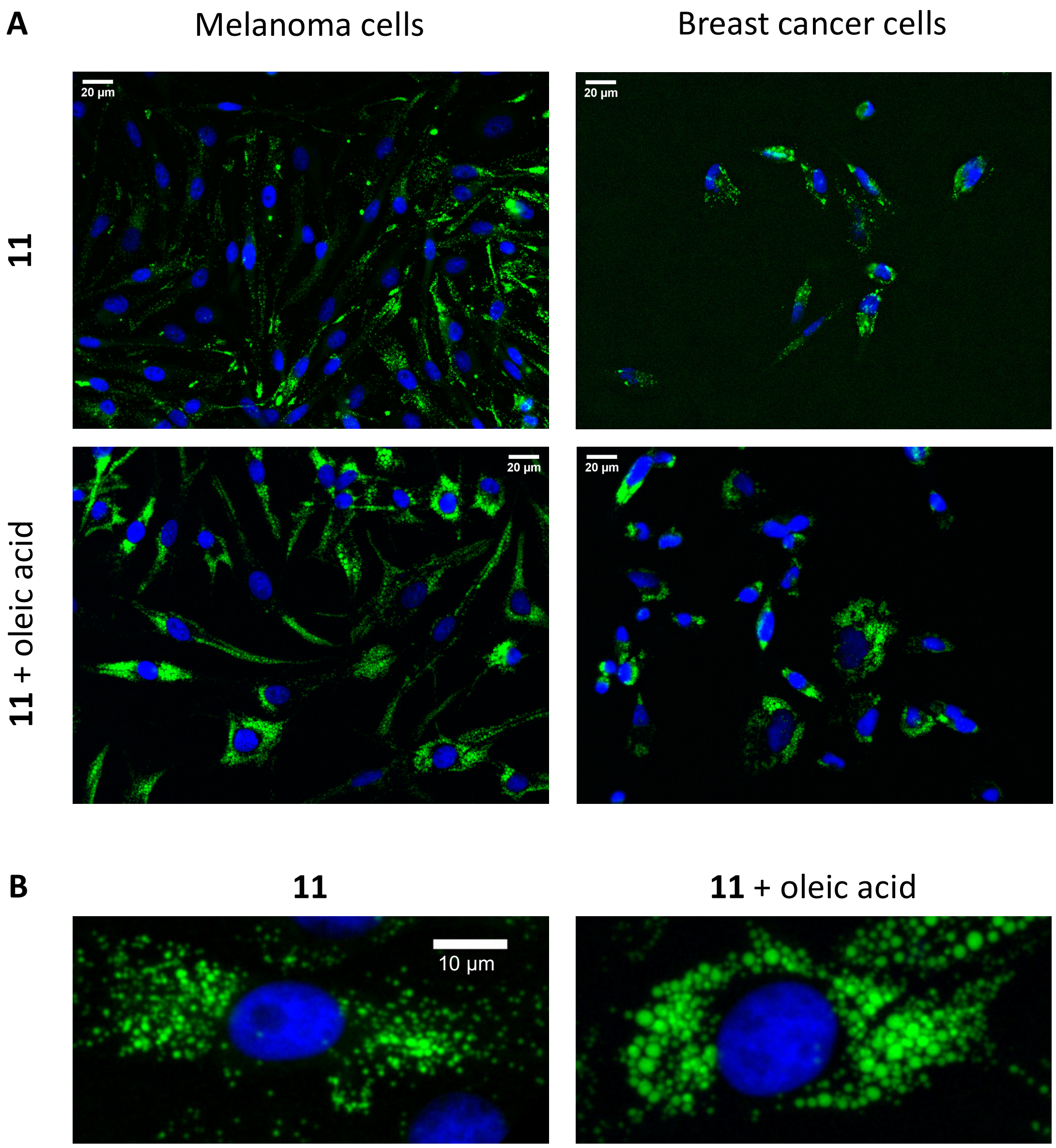

Figure S28. A) Fluorescence microscopy images of melanoma (SK-MEL-28) and breast cancer (MDA-MB-231) cells stained with **11** (seen in green; ex. 460-500 nm, em. 512-542 nm). The bottom row shows cells that have been supplemented with oleic acid (100 µM, 24 h) before fixation. B) Magnified regions of the melanoma cell images in A. Staining was performed on fixed cells (10 µM, 1 h). Cell nuclei were stained with DAPI (seen in blue; ex. 325-375 nm, em. 435-485 nm).

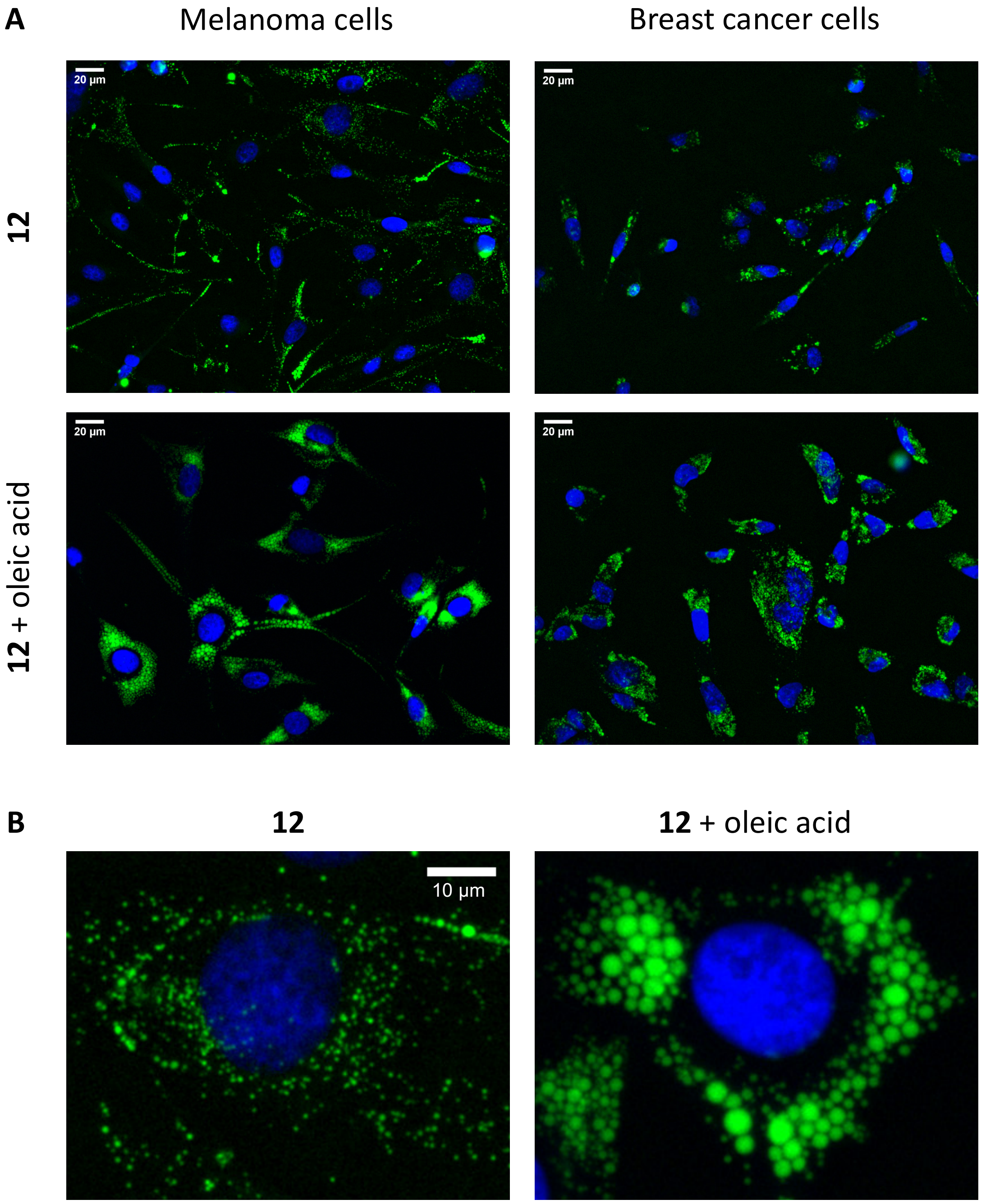

Figure S29. A) Fluorescence microscopy images of melanoma (SK-MEL-28) and breast cancer (MDA-MB-231) cells stained with **12** (seen in green; ex. 460-500 nm, em. 512-542 nm). The bottom row shows cells that have been supplemented with oleic acid (100 µM, 24 h) before fixation. B) Magnified regions of the melanoma cell images in A. Staining was performed on fixed cells (10 µM, 1 h). Cell nuclei were stained with DAPI (seen in blue; ex. 325-375 nm, em. 435-485 nm).

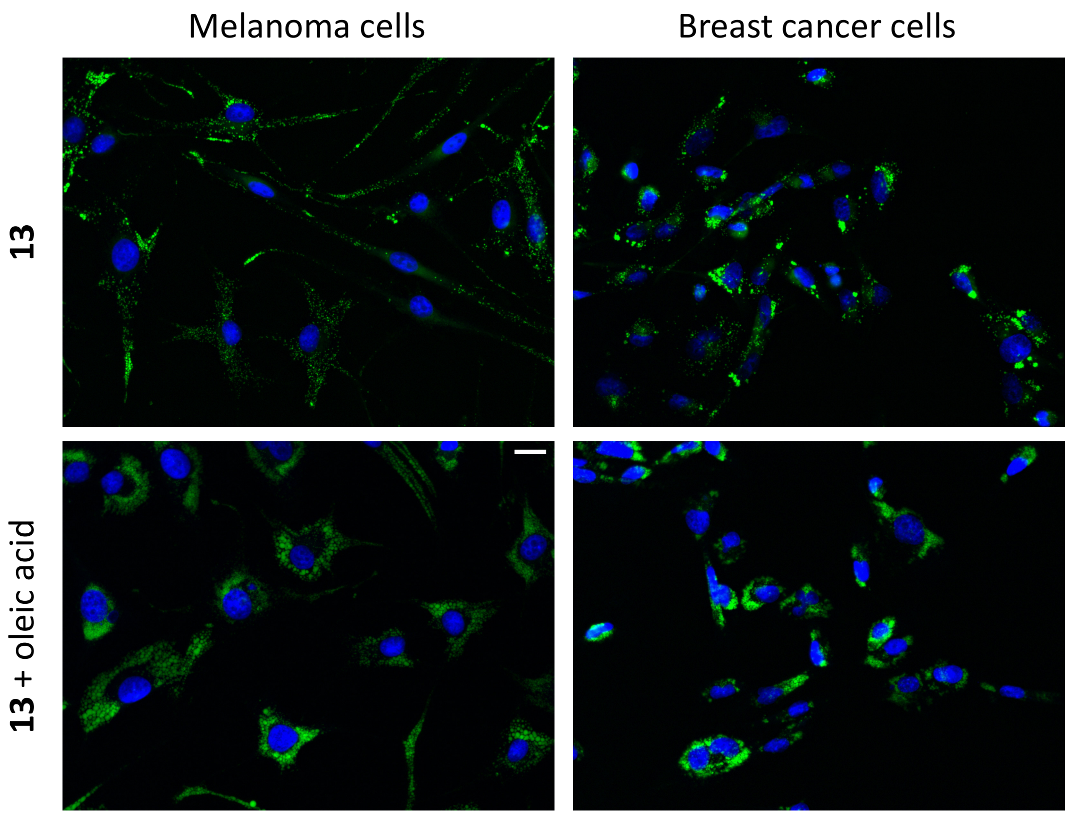

Figure S30. Fluorescence microscopy images of melanoma (SK-MEL-28) and breast cancer (MDA-MB-231) cells stained with **13** (seen in green; ex. 460-500 nm, em. 512-542 nm). The bottom row shows cells that have been supplemented with oleic acid (100 μM, 24 h) before fixation. Scale bar 20 μm. Staining was performed on fixed cells (10 μM, 1 h). Cell nuclei were stained with DAPI (seen in blue; ex. 325-375 nm, em. 435-485 nm).

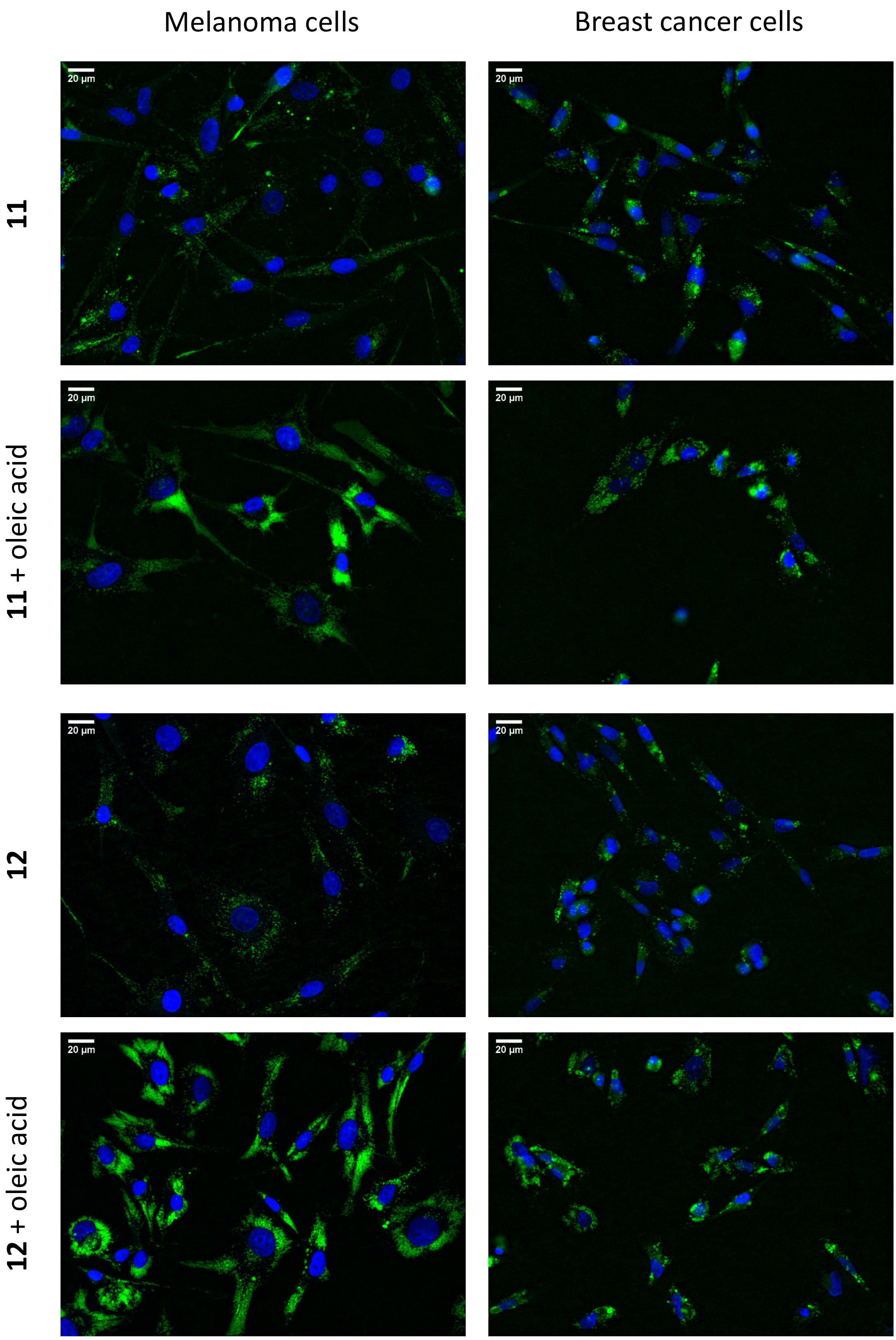

Figure S31. Fluorescence microscopy images of melanoma (SK-MEL-28) and breast cancer (MDA-MB-231) cells stained with **11** (top half) or **12** (bottom half) (seen in green; ex. 460-500 nm, em. 512-542 nm). The bottom row for each compound shows cells that have been supplemented with oleic acid (100 µM, 24 h). Staining was performed on living cells (10 µM, 24 h). Cell nuclei were stained with DAPI (seen in blue; ex. 325-375 nm, em. 435-485 nm).

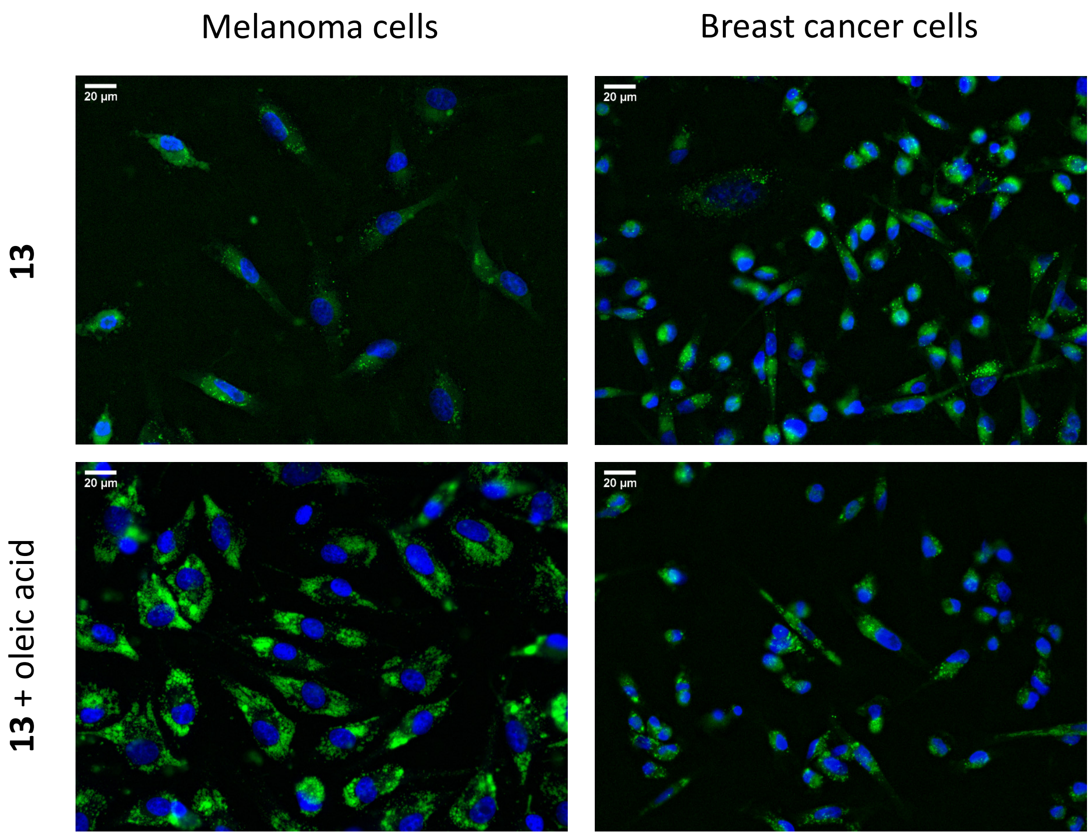

Figure S32. Fluorescence microscopy images of melanoma (SK-MEL-28) and breast cancer (MDA-MB-231) cells stained with **13** (seen in green; ex. 460-500 nm, em. 512-542 nm). The bottom row shows cells that have been supplemented with oleic acid (100 µM, 24 h). Staining was performed on living cells (10 µM, 24 h). Cell nuclei were stained with DAPI (seen in blue; ex. 325-375 nm, em. 435-485 nm).

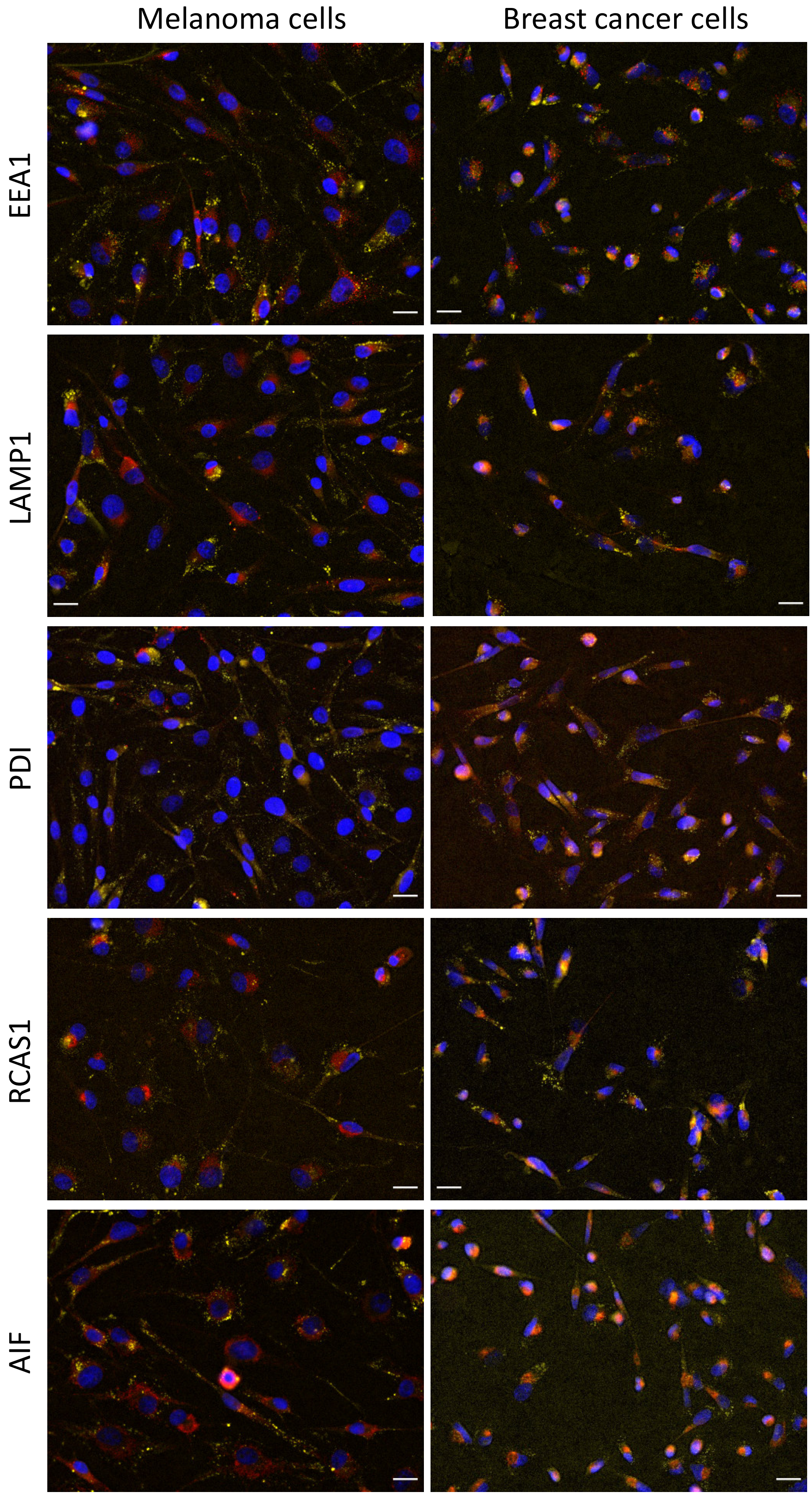

Figure S33. Colocalization experiments of **11** (30 µM, 24 h; seen in yellow; ex. 460-500 nm, em. 512-542 nm) with EEA1 (endosomes), LAMP1 (lysosomes), PDI (endoplasmic reticulum), RCAS1 (Golgi) or AIF (mitochondria) (seen in red; ex. 590-650 nm, em. 662-738 nm). Scale bar 20 µm. Cell nuclei were stained with DAPI (seen in blue; ex. 325-375 nm, em. 435-485 nm).

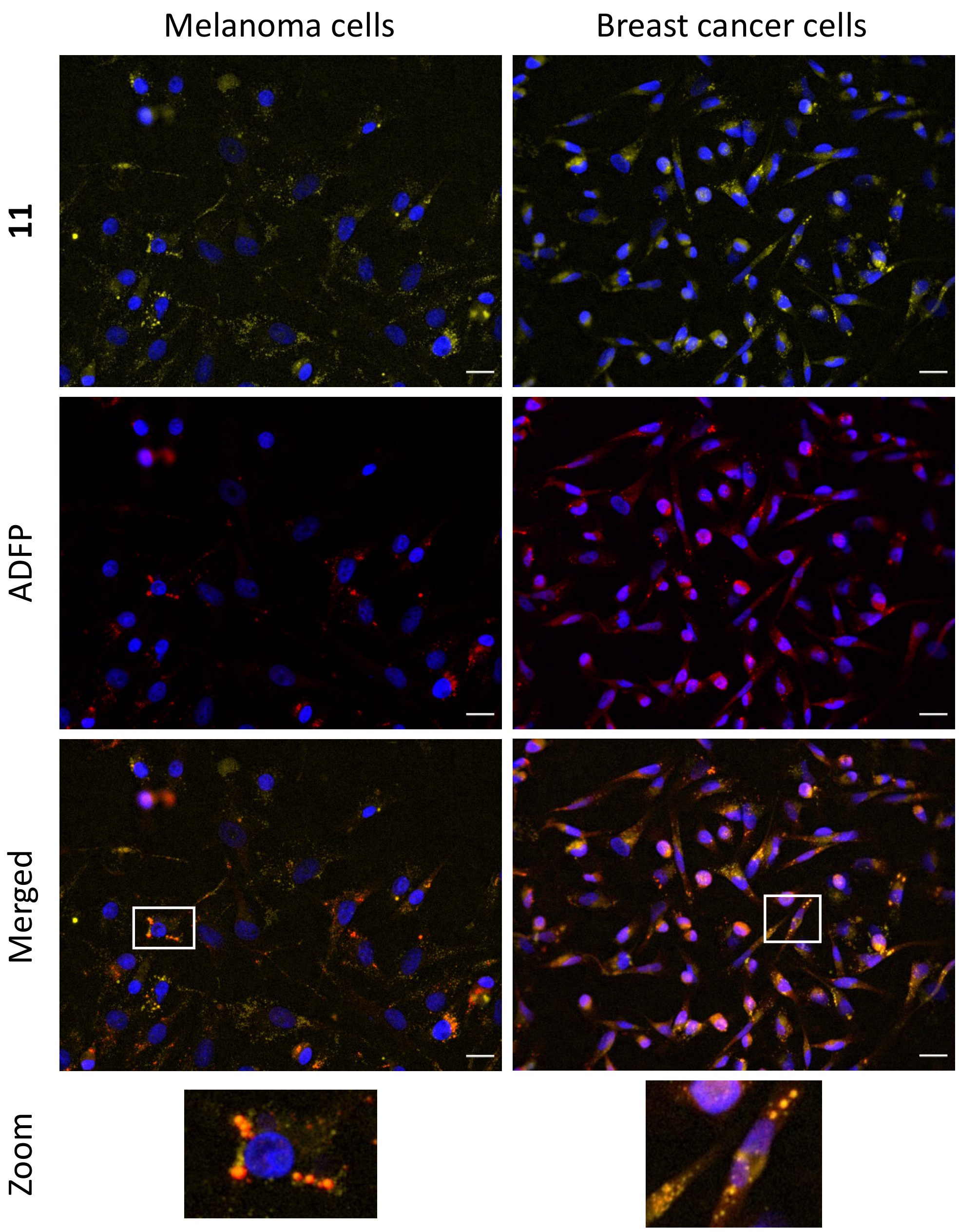

Figure S34. Fluorescence microscopy images of the colocalization of **11** (seen in yellow; ex. 460-500 nm, em. 512-542 nm) and an anti-ADFP antibody that targets the surface of lipid droplets (seen in red; ex. 590-650 nm, em. 662-738 nm) in melanoma (SK-MEL-28) and breast cancer (MDA-MB-231) cells. Scale bar 20 µm. Staining was performed on live cells, which were fixed after incubation (30 µM, 24 h) and then subjected to immunocytochemistry. Cell nuclei were stained with DAPI (seen in blue; ex. 325-375 nm, em. 435-485 nm).

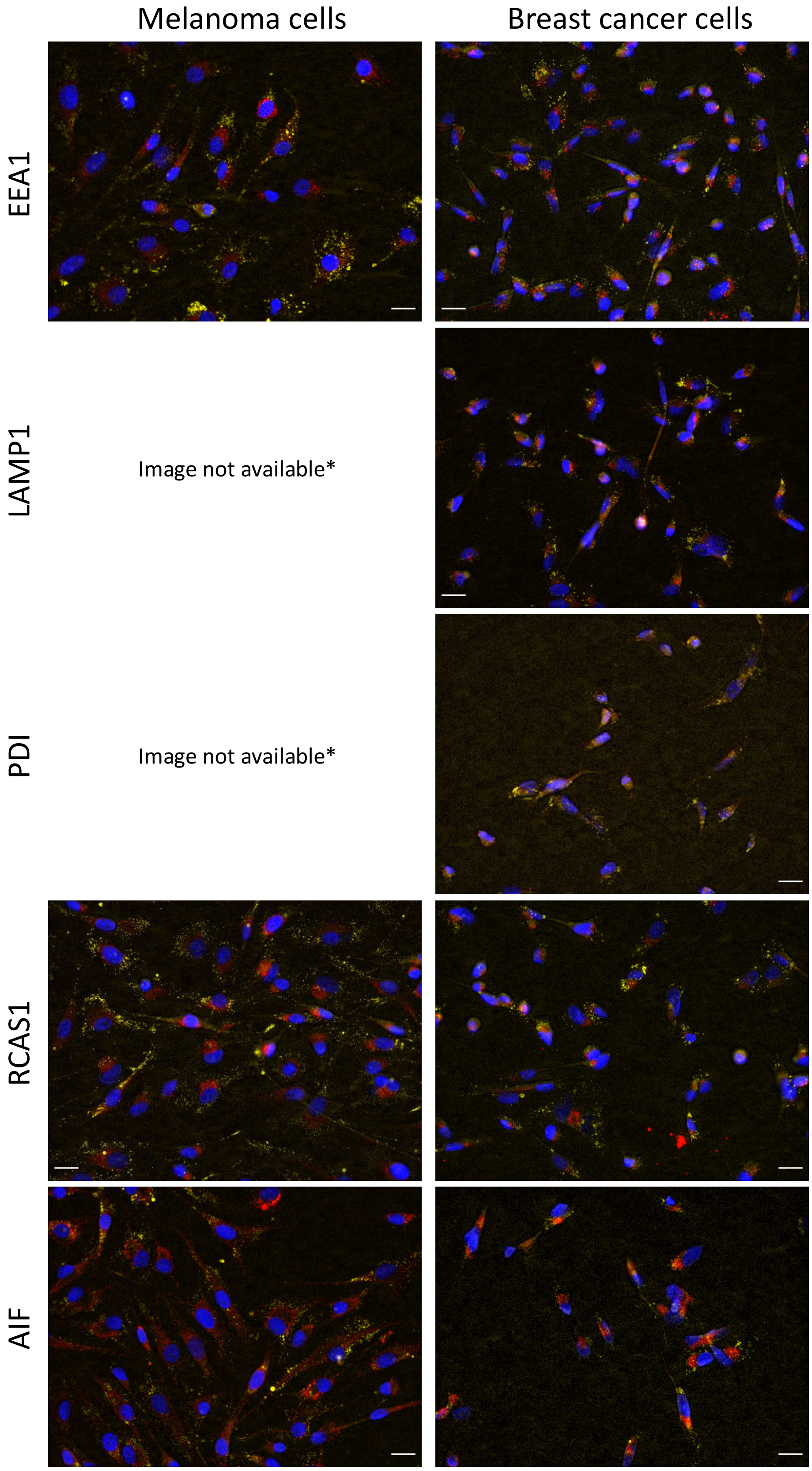

Figure S35. Colocalization experiments of **12** (30 µM, 24 h; seen in yellow; ex. 460-500 nm, em. 512-542 nm) with EEA1 (endosomes), LAMP1 (lysosomes), PDI (endoplasmic reticulum), RCAS1 (Golgi) or AIF (mitochondria) (seen in red; ex. 590-650 nm, em. 662-738 nm). Scale bar 20 µm. Cell nuclei were stained with DAPI (seen in blue; ex. 325-375 nm, em. 435-485 nm). *The experiment was inconclusive and not investigated further since results with breast cancer cells were obtained and good colocalization with lipid droplets was observed.

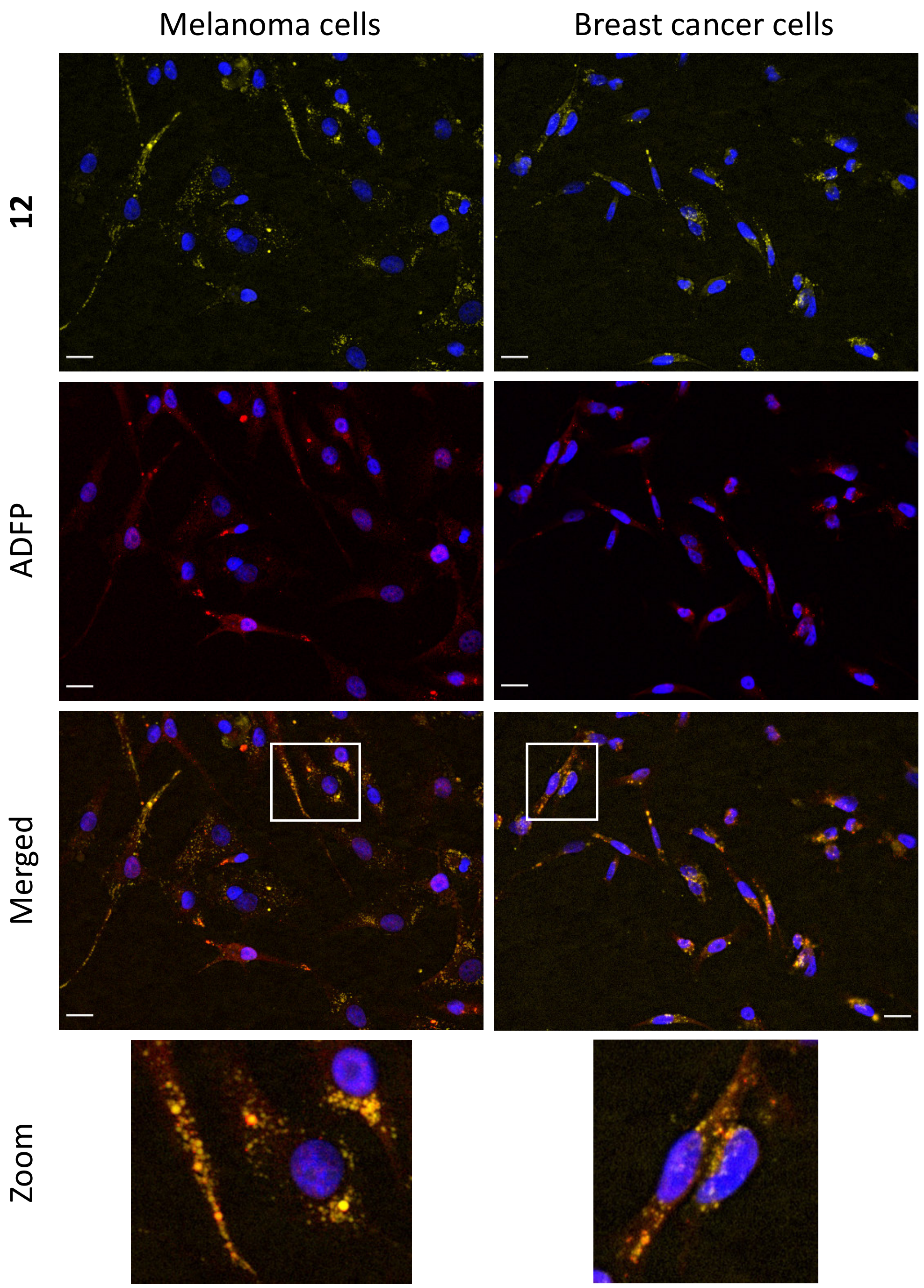

Figure S36. Fluorescence microscopy images of the colocalization of **12** (seen in yellow; ex. 460-500 nm, em. 512-542 nm) and an anti-ADFP antibody that targets the surface of lipid droplets (seen in red; ex. 590-650 nm, em. 662-738 nm) in melanoma (SK-MEL-28) and breast cancer (MDA-MB-231) cells. Scale bar 20 µm. Staining was performed on live cells, which were fixed after incubation (30 µM, 24 h) and then subjected to immunocytochemistry. Cell nuclei were stained with DAPI (seen in blue; ex. 325-375 nm, em. 435-485 nm).

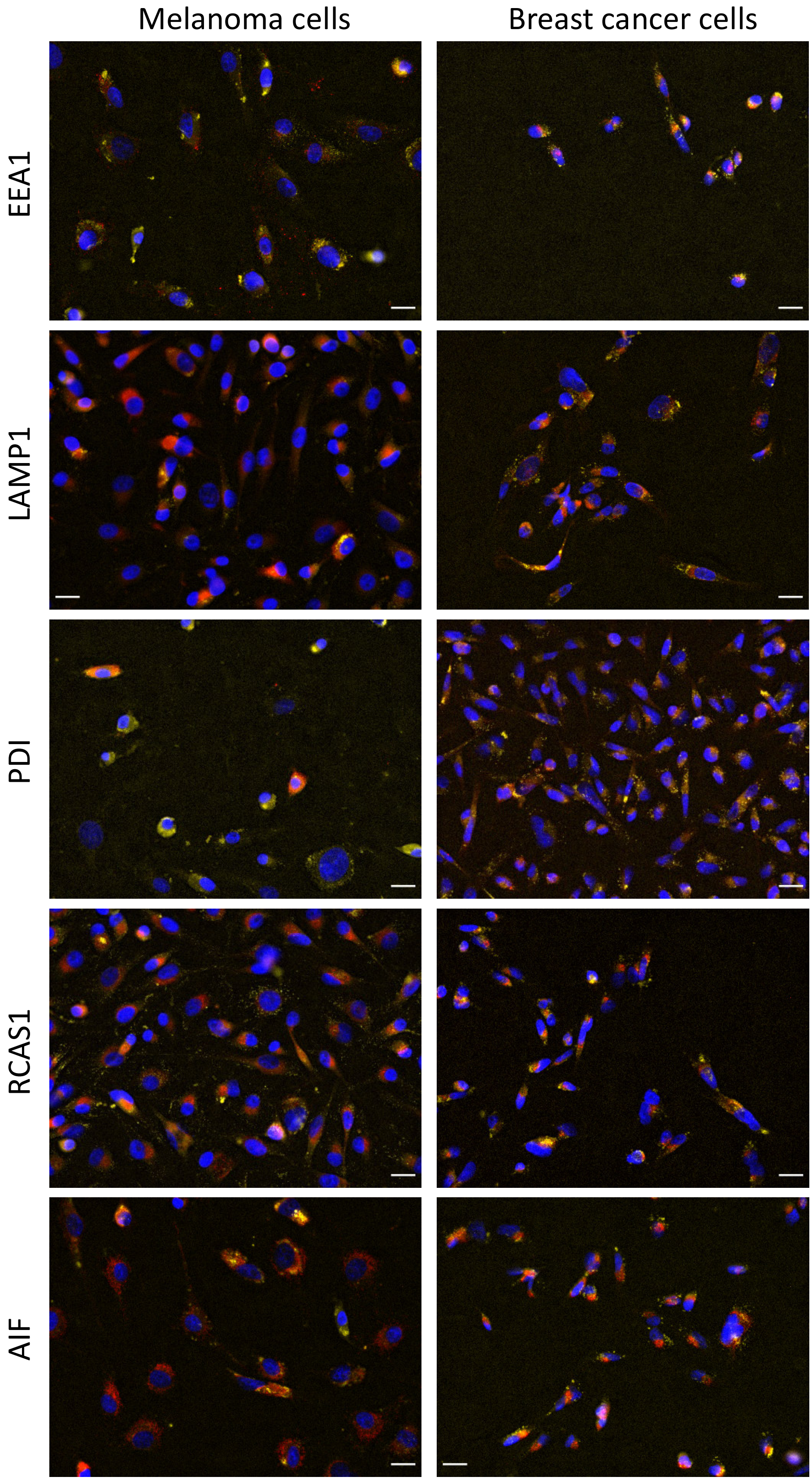

Figure S37. Colocalization experiments of **13** (10 µM, 24 h; seen in yellow; ex. 460-500 nm, em. 512-542 nm)* with EEA1 (endosomes), LAMP1 (lysosomes), PDI (endoplasmic reticulum), RCAS1 (Golgi) or AIF (mitochondria) (seen in red; ex. 590-650 nm, em. 662-738 nm). Scale bar 20 µm. *30 µM of **13** was used in costaining of melanoma cells with LAMP1 and PDI. Cell nuclei were stained with DAPI (seen in blue; ex. 325-375 nm, em. 435-485 nm).

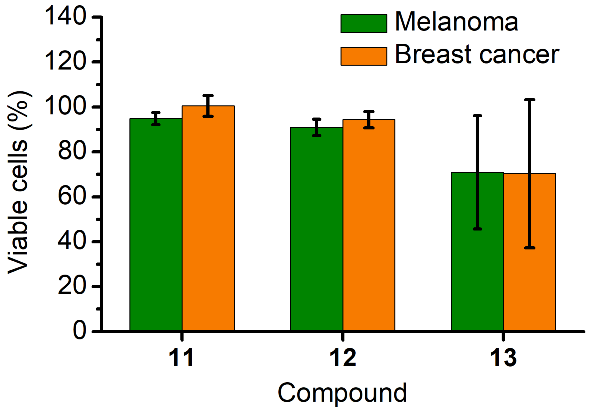

Figure S38. Cell viability of melanoma (SK-MEL-28) and breast cancer cells (MDA-MB-231) after treatment with **11**, **12** and **13** (10 µM, 24 h), respectively. The data is presented relative to DMSO control as a mean ± SD; n = 3 (**11**, **12**) or 4 (**13**).

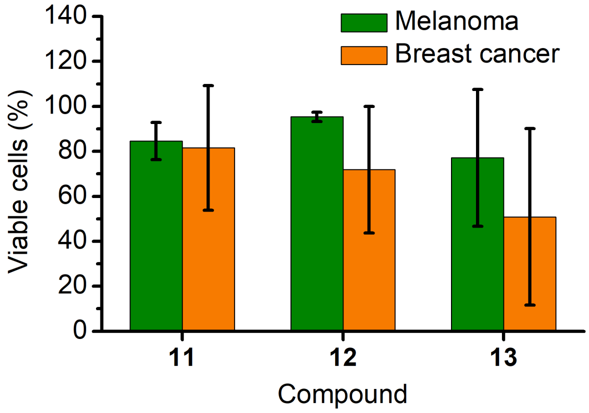

Figure S39. Cell viability of melanoma (SK-MEL-28) and breast cancer cells (MDA-MB-231) after treatment with **11**, **12** and **13** (30 µM, 24 h), respectively. The data is presented relative to DMSO control as a mean ± SD; n = 5 (**11**, **12**) or 6 (**13**).

VII. NMR Spectra

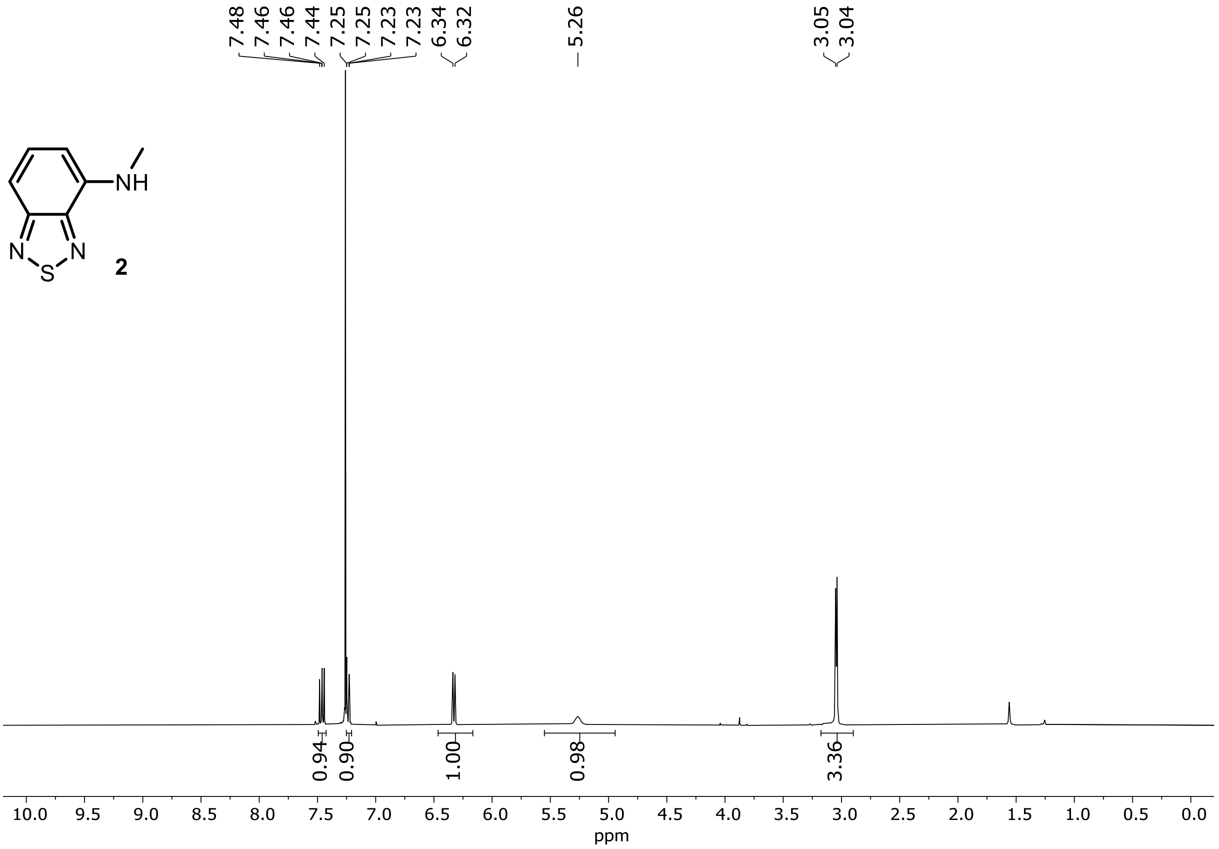

Figure S40. ^{1}H NMR spectrum (400 MHz, $CDCl_3$) of compound **2**.

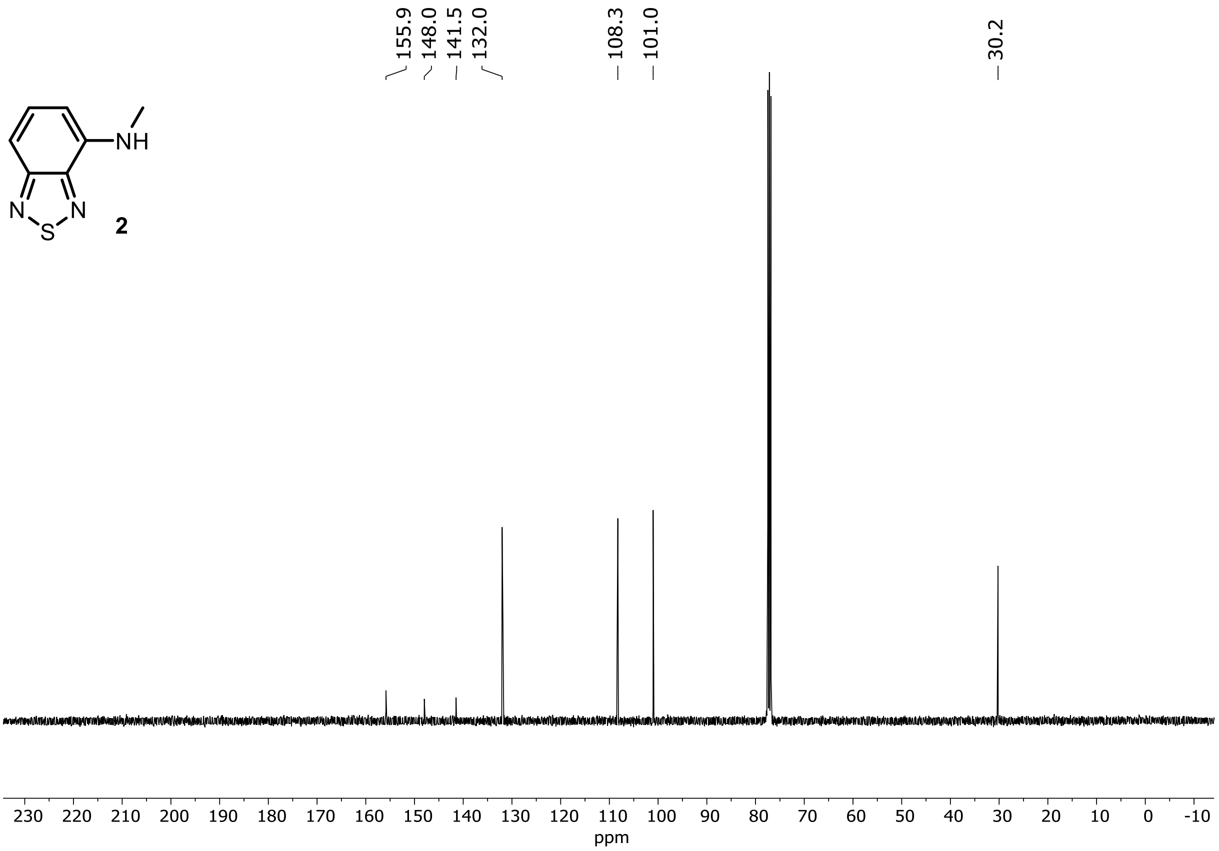

Figure S41. ^{13}C NMR spectrum (101 MHz, $CDCl_3$) of compound **2**.

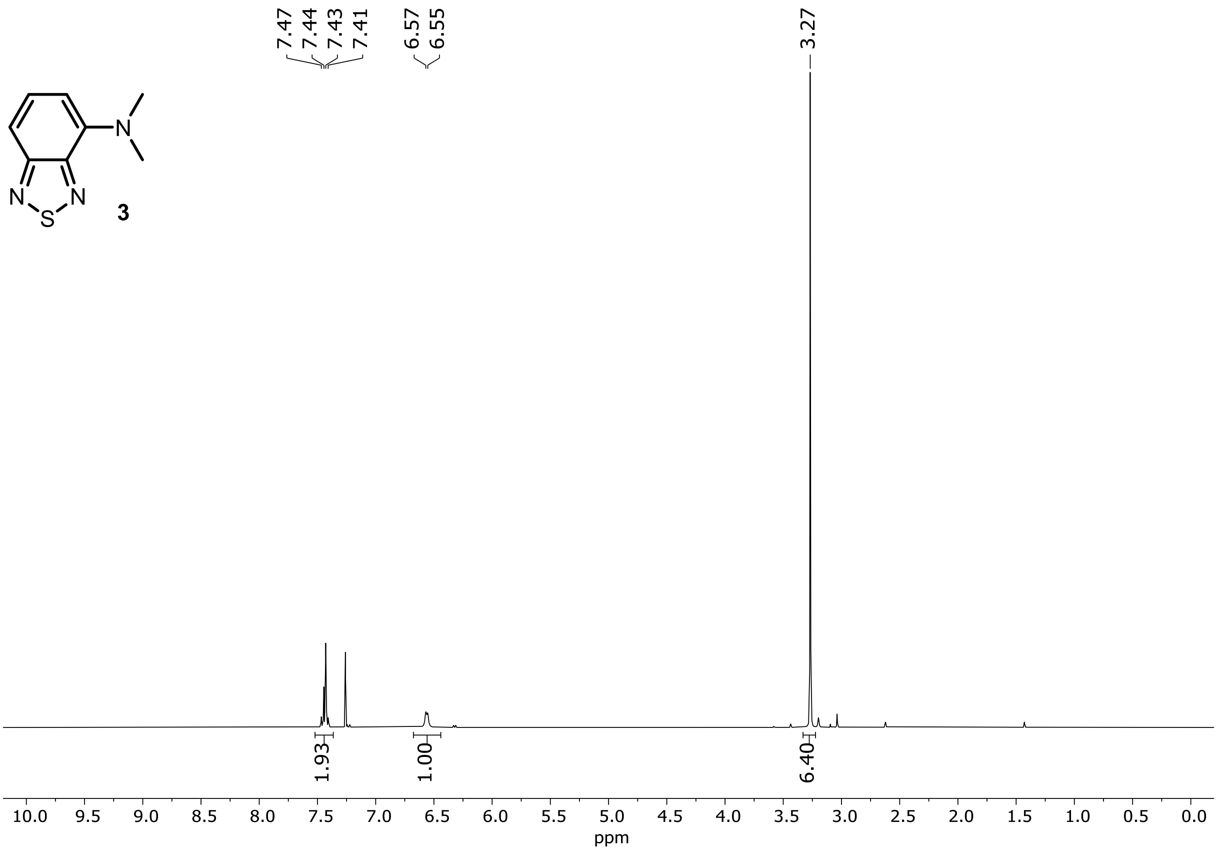

Figure S42. ^{1}H NMR spectrum (400 MHz, $CDCl_3$) of compound **3**.

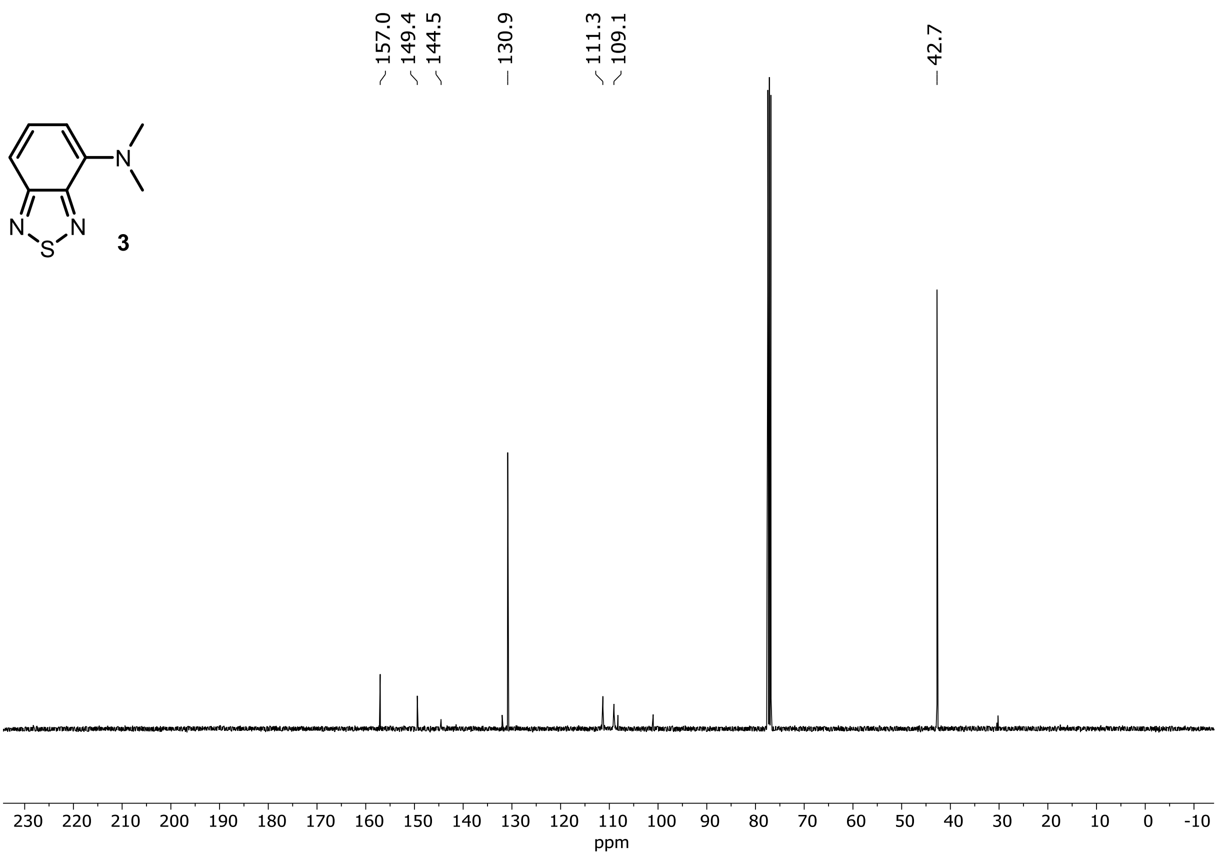

Figure S43. ^{13}C NMR spectrum (101 MHz, $CDCl_3$) of compound **3**. Additional peaks in the spectrum can be ascribed to **2** (*cf.* Figure S41), which could not be fully removed during purification (*ca.* 5% based on ^1H NMR).

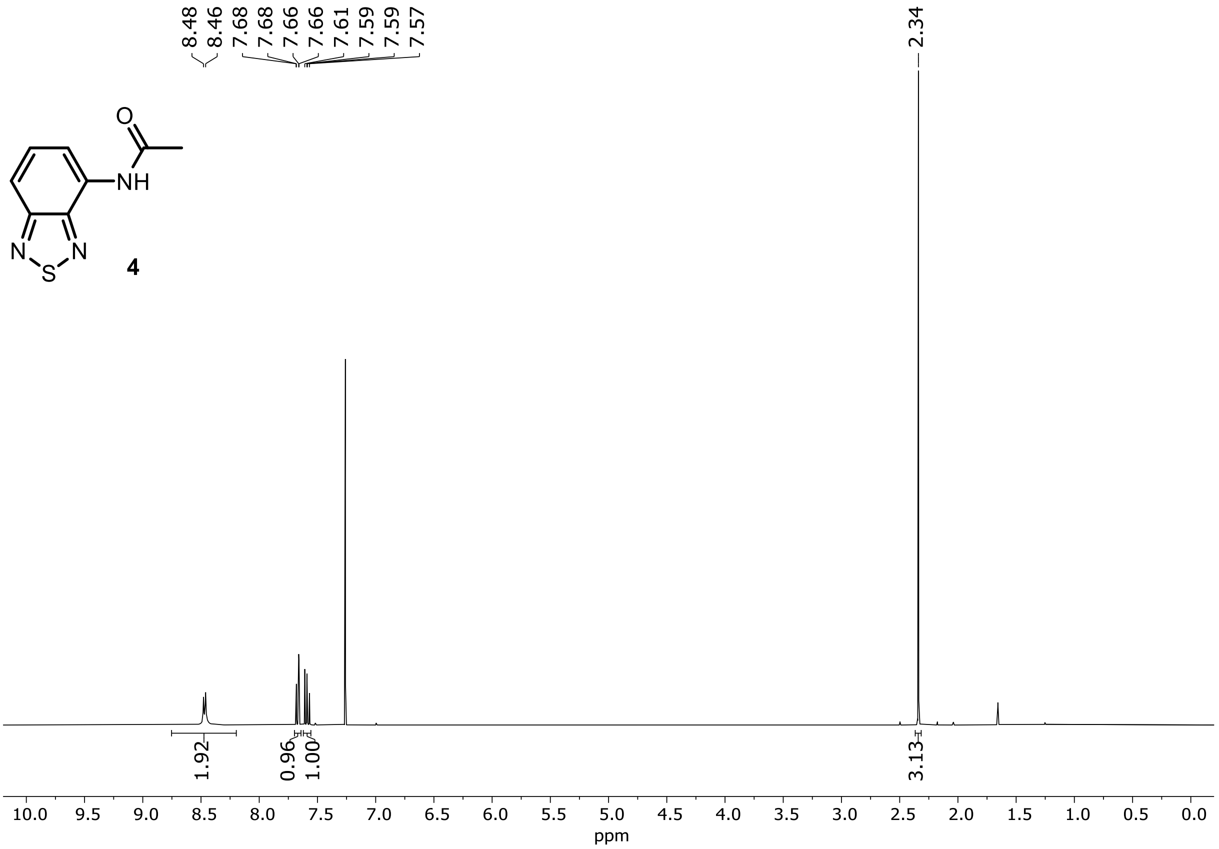

Figure S44. ^1H NMR spectrum (400 MHz, $CDCl_3$) of compound **4**.

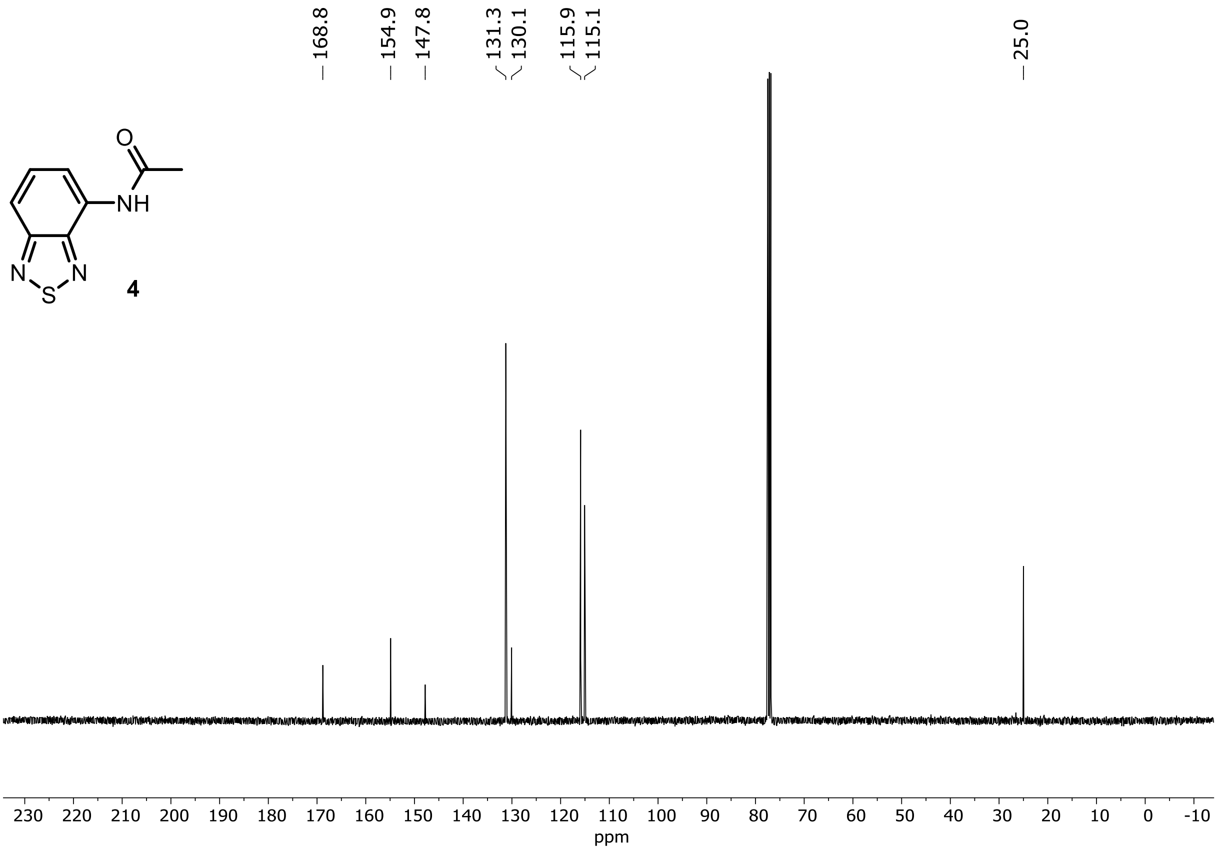

Figure S45. ^{13}C NMR spectrum (101 MHz, $CDCl_3$) of compound **4**.

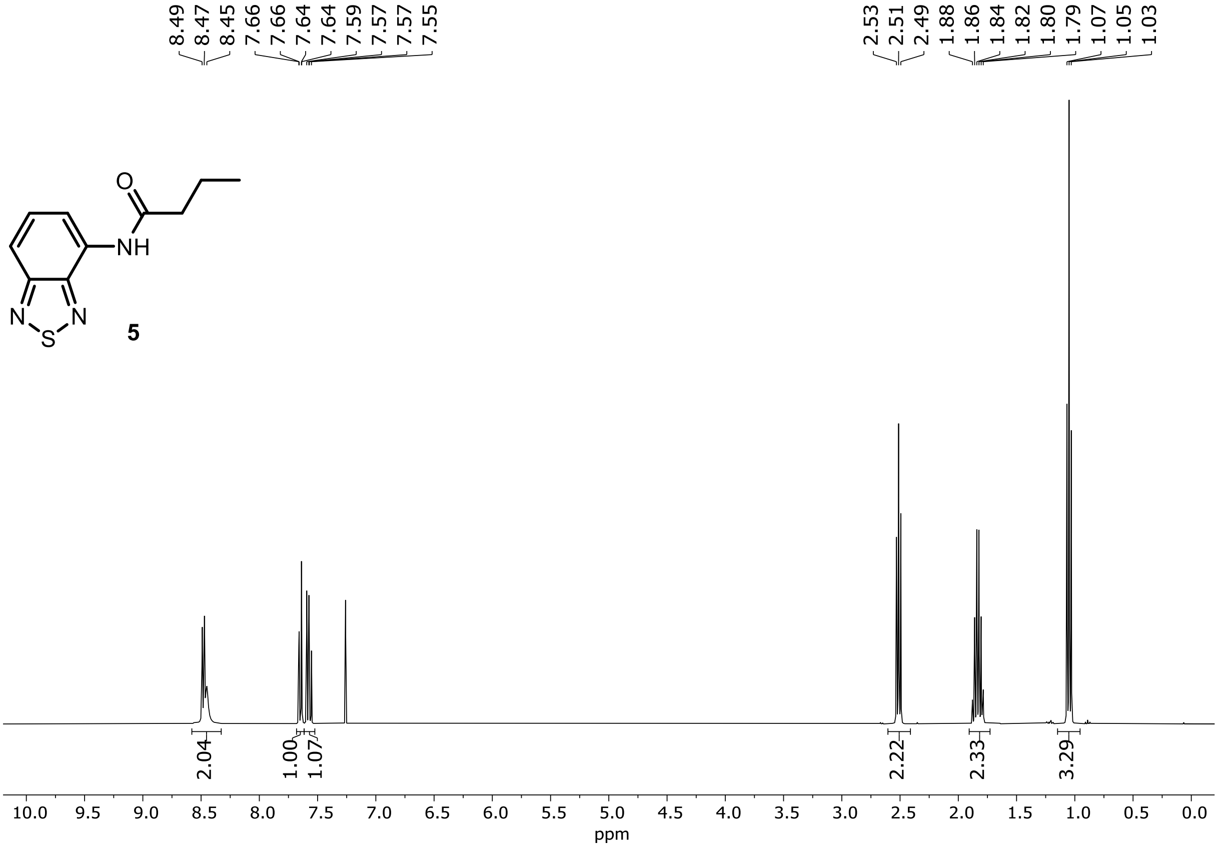

Figure S46. ^{1}H NMR spectrum (400 MHz, $CDCl_3$) of compound **5**.

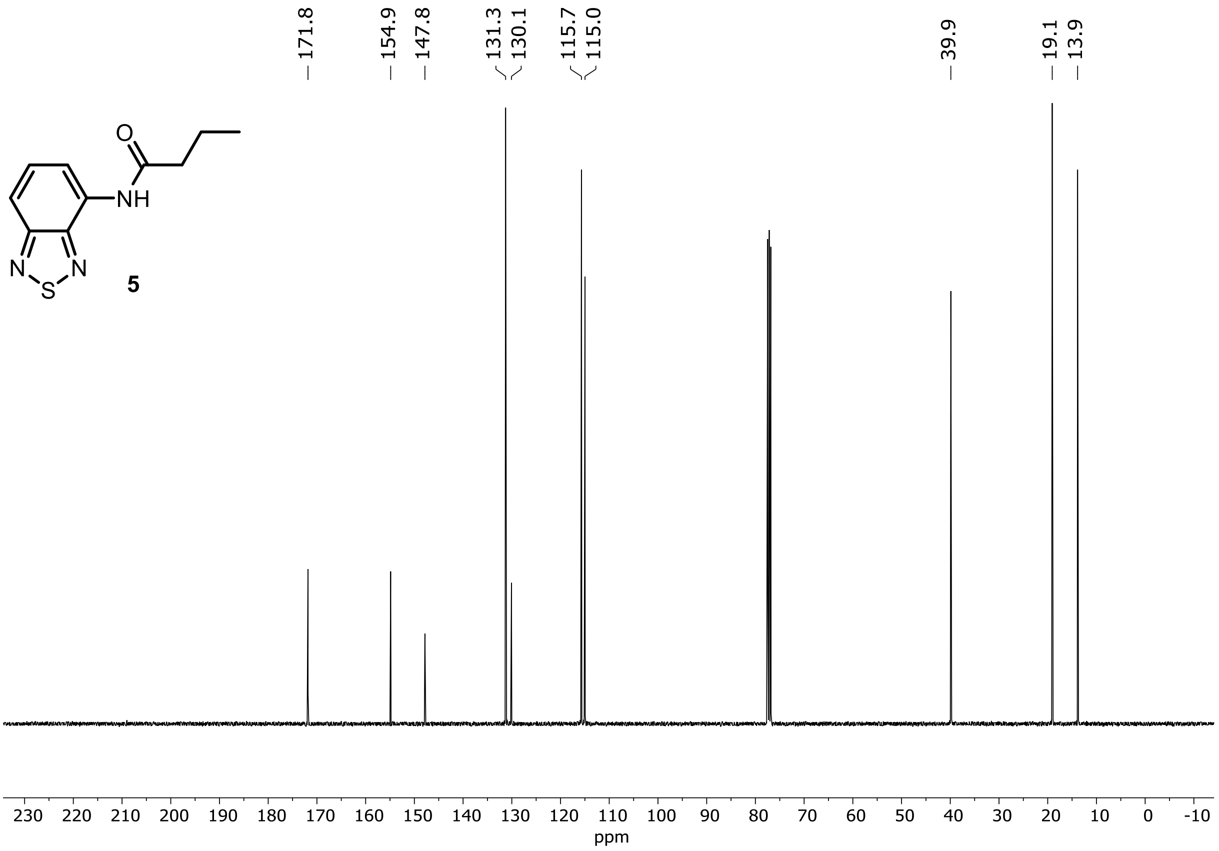

Figure S47. ^{13}C NMR spectrum (101 MHz, $CDCl_3$) of compound **5**.

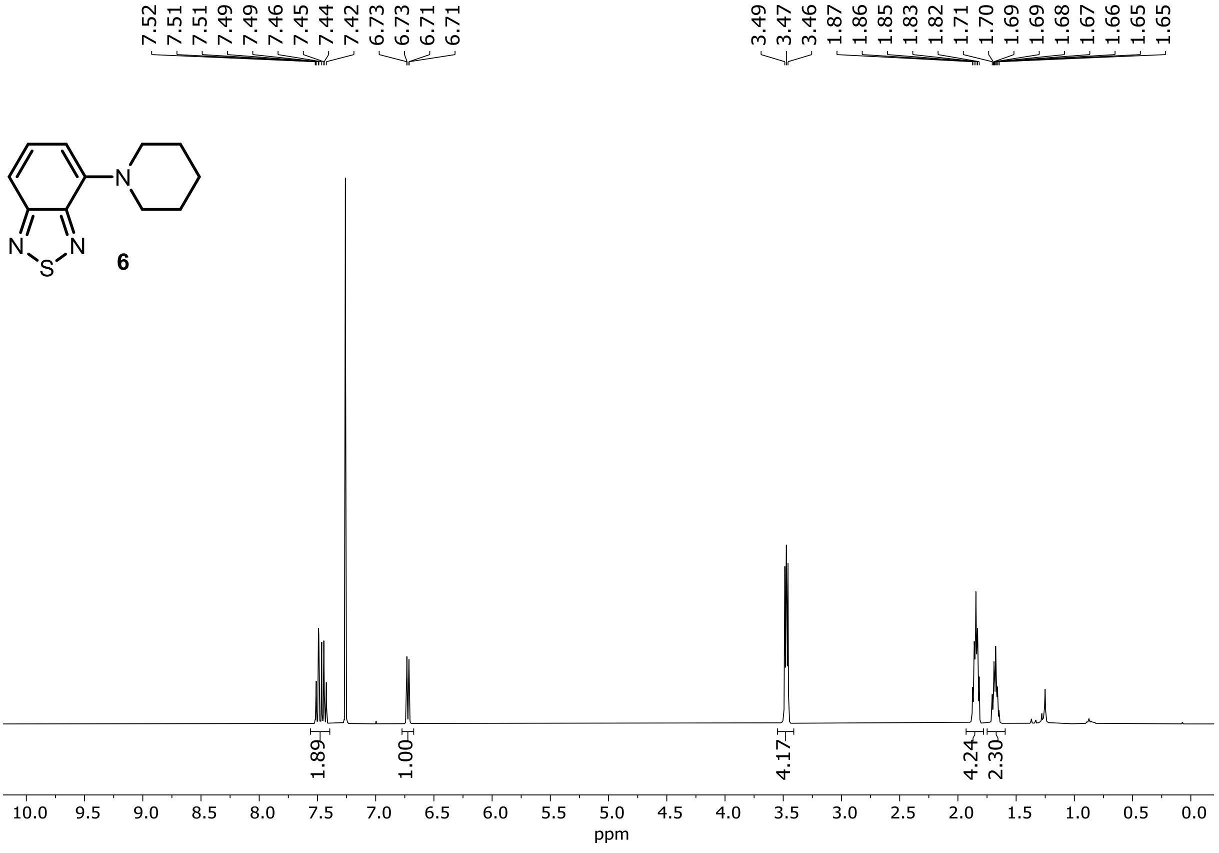

Figure S48. ^{1}H NMR spectrum (400 MHz, $CDCl_3$) of compound **6**.

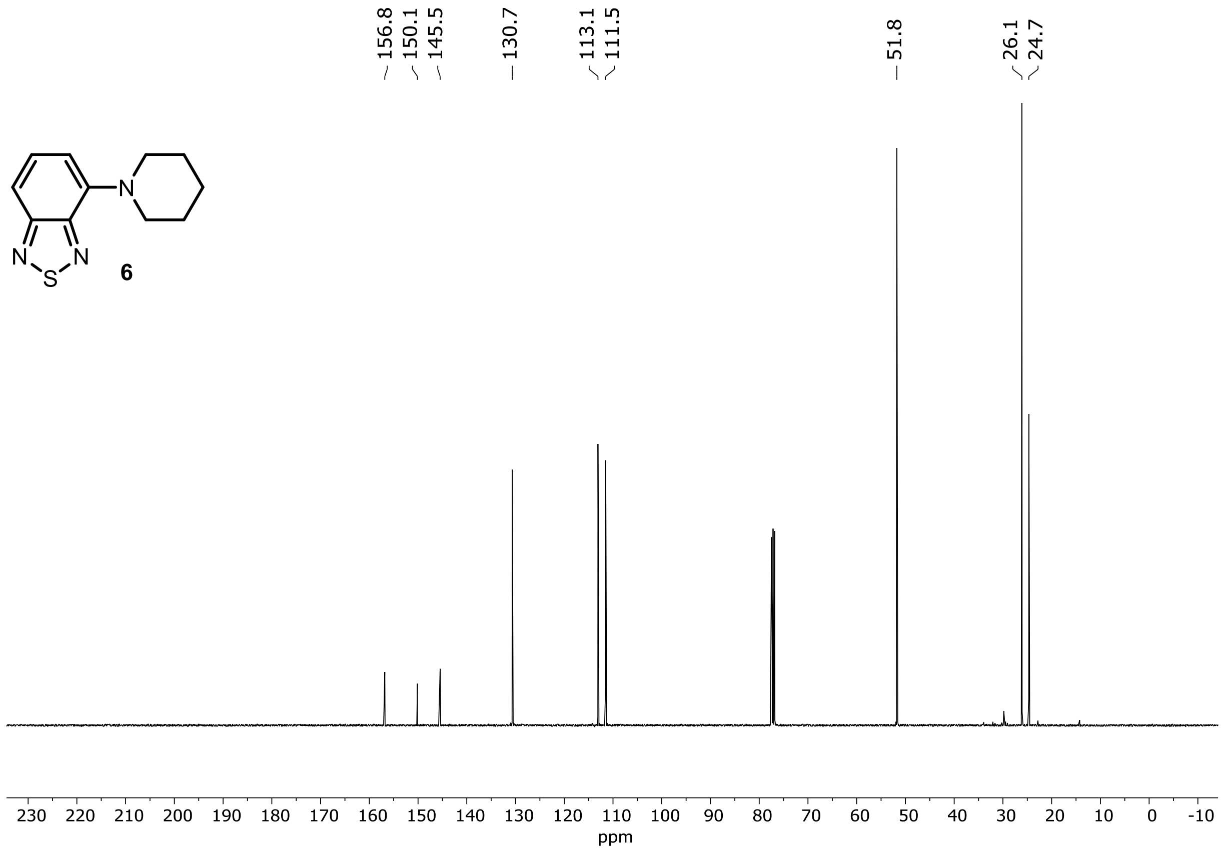

Figure S49. ^{13}C NMR spectrum (101 MHz, $CDCl_3$) of compound **6**.

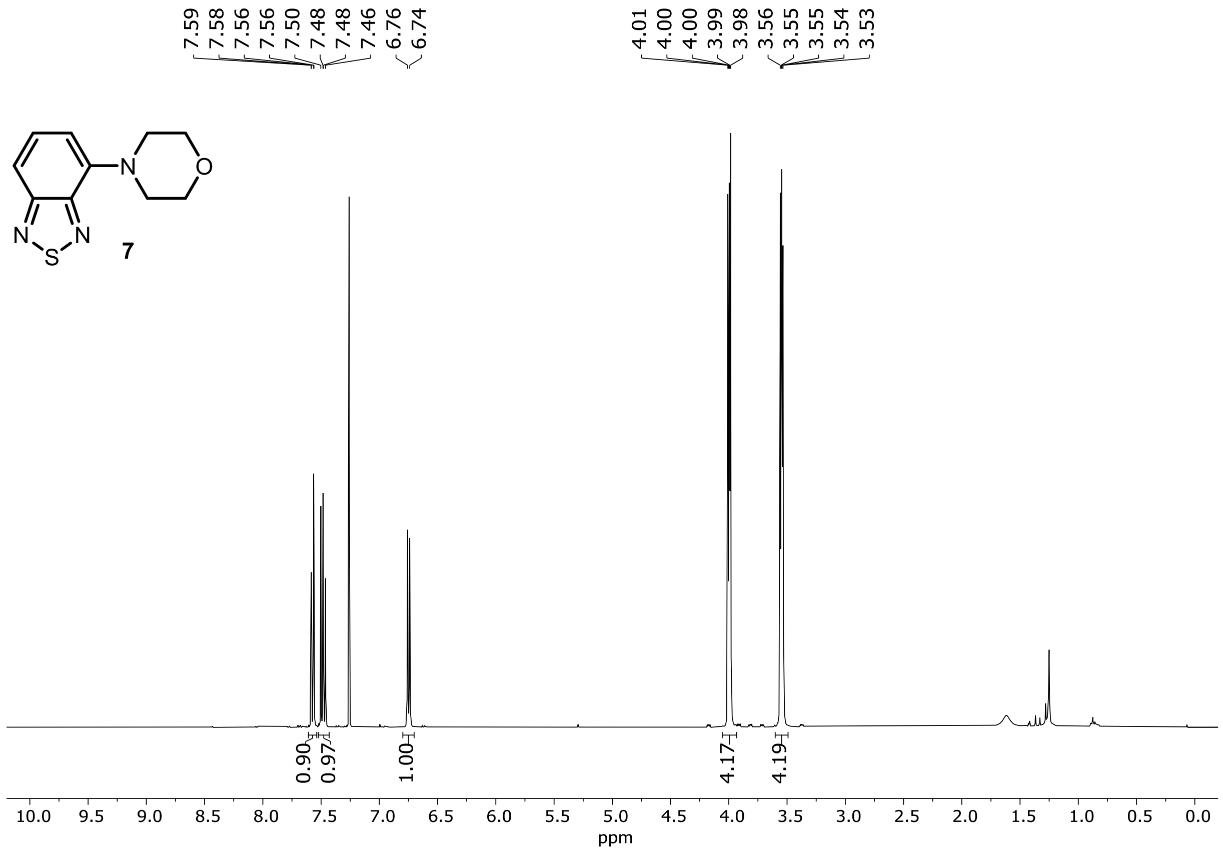

Figure S50. ^{1}H NMR spectrum (400 MHz, $CDCl_3$) of compound **7**.

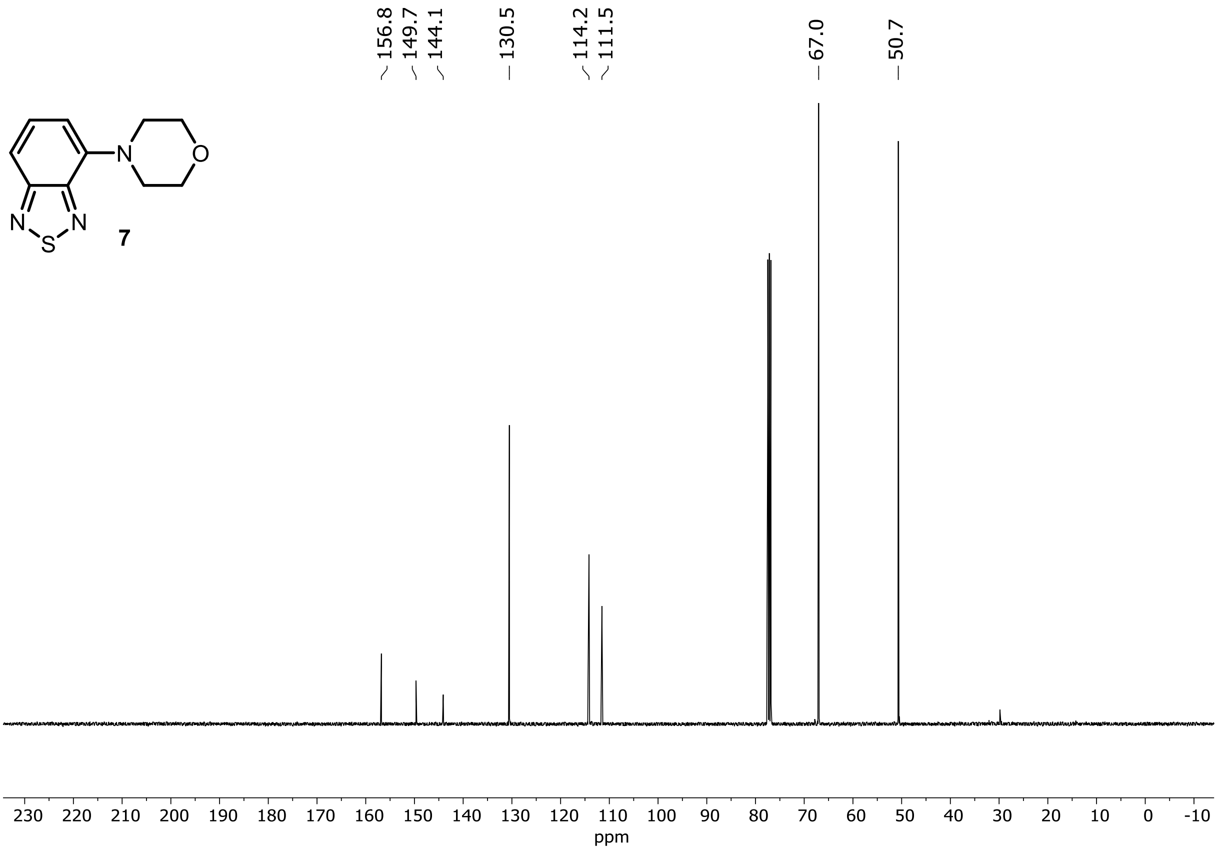

Figure S51. ^{13}C NMR spectrum (101 MHz, $CDCl_3$) of compound **7**.

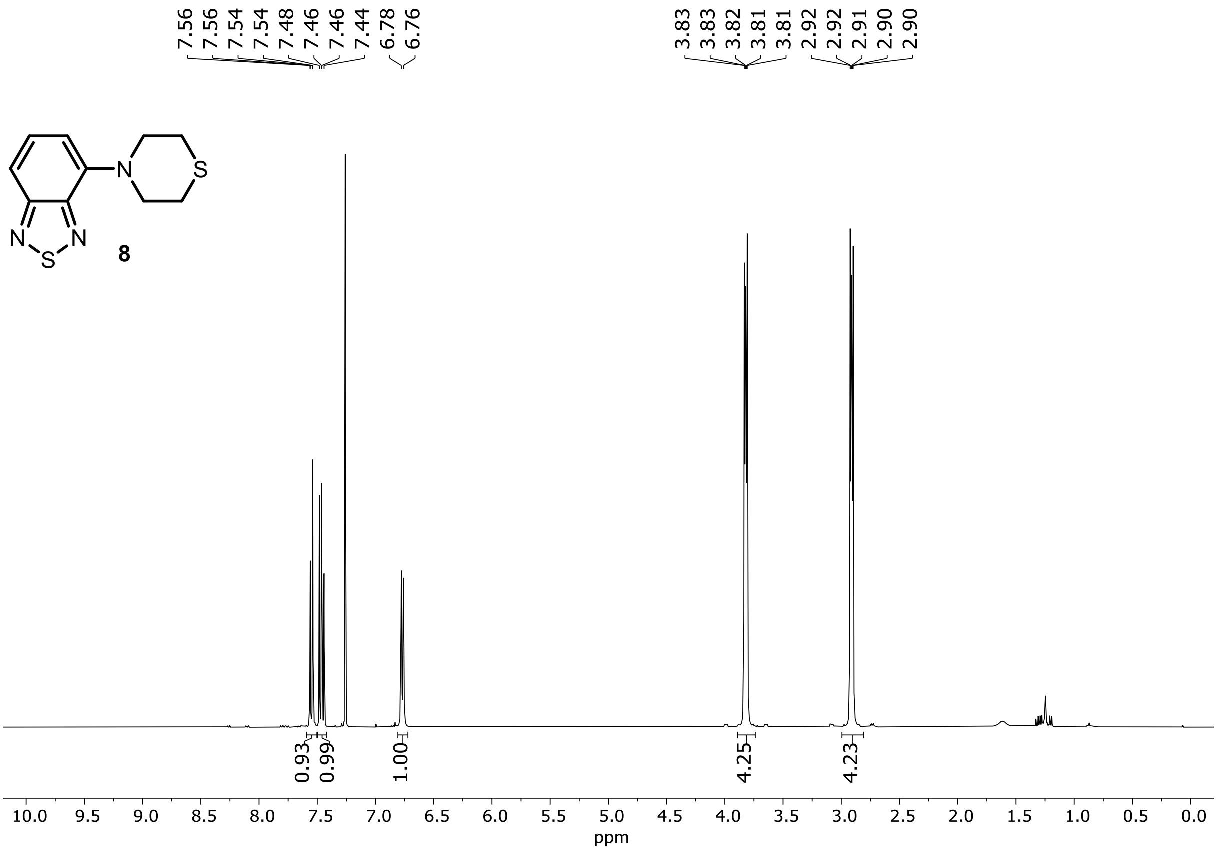

Figure S52. ^{1}H NMR spectrum (400 MHz, $CDCl_3$) of compound **8**.

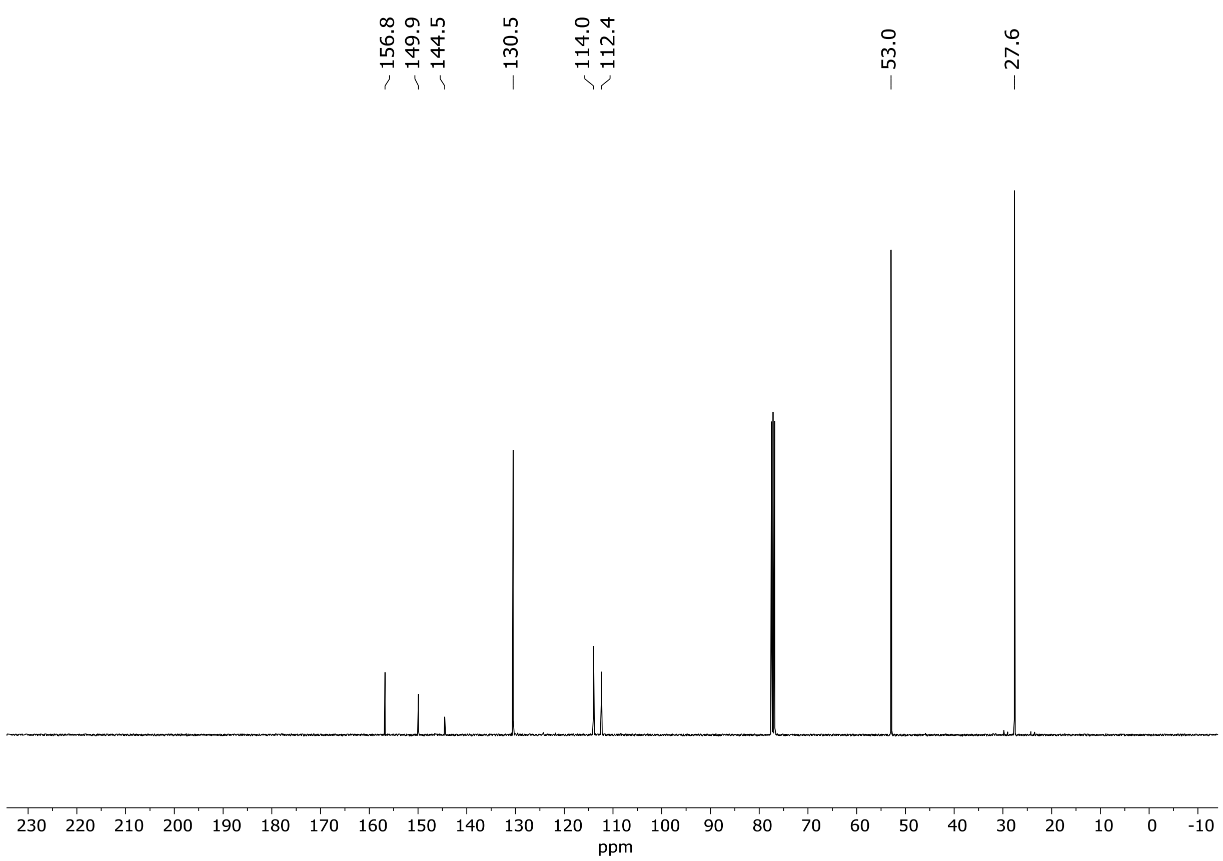

Figure S53. ^{13}C NMR spectrum (101 MHz, $CDCl_3$) of compound **8**.

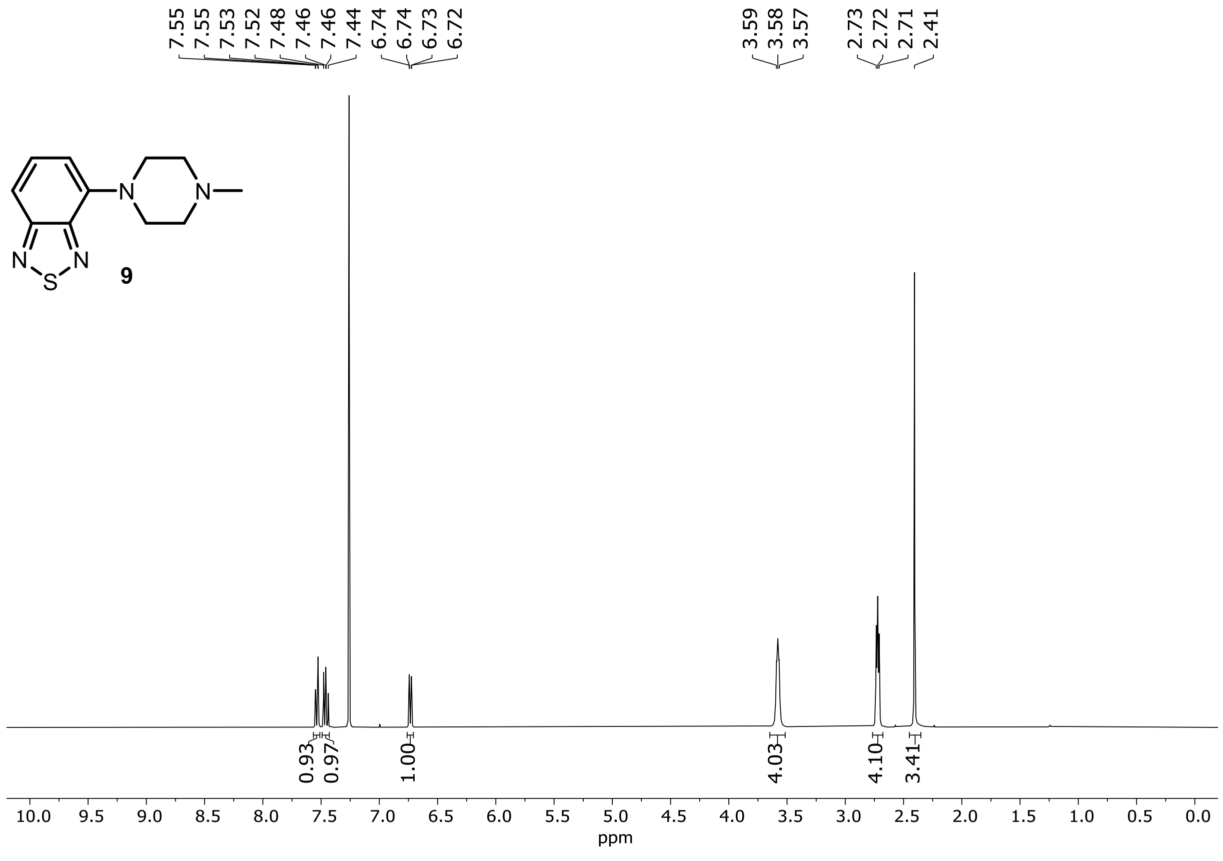

Figure S54. ^{1}H NMR spectrum (400 MHz, $CDCl_3$) of compound **9**.

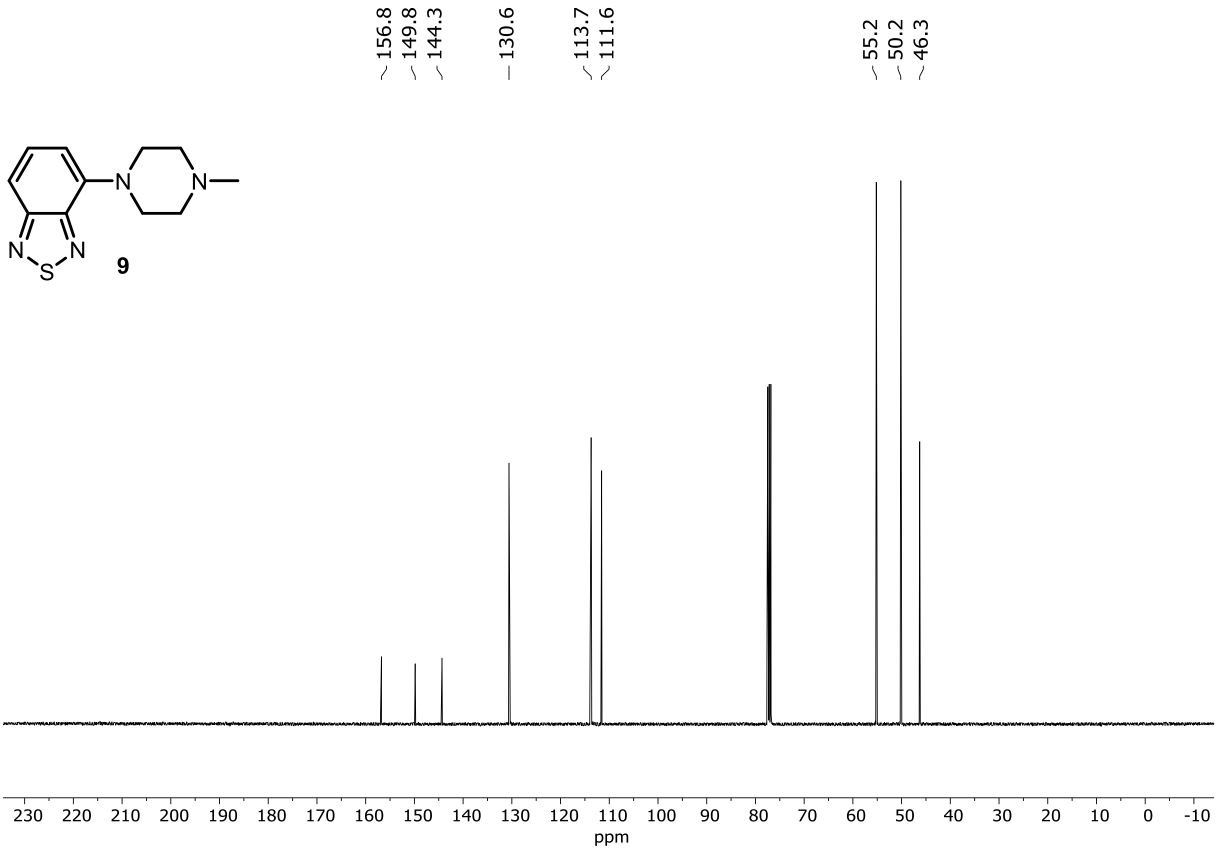

Figure S55. ^{13}C NMR spectrum (101 MHz, $CDCl_3$) of compound **9**.

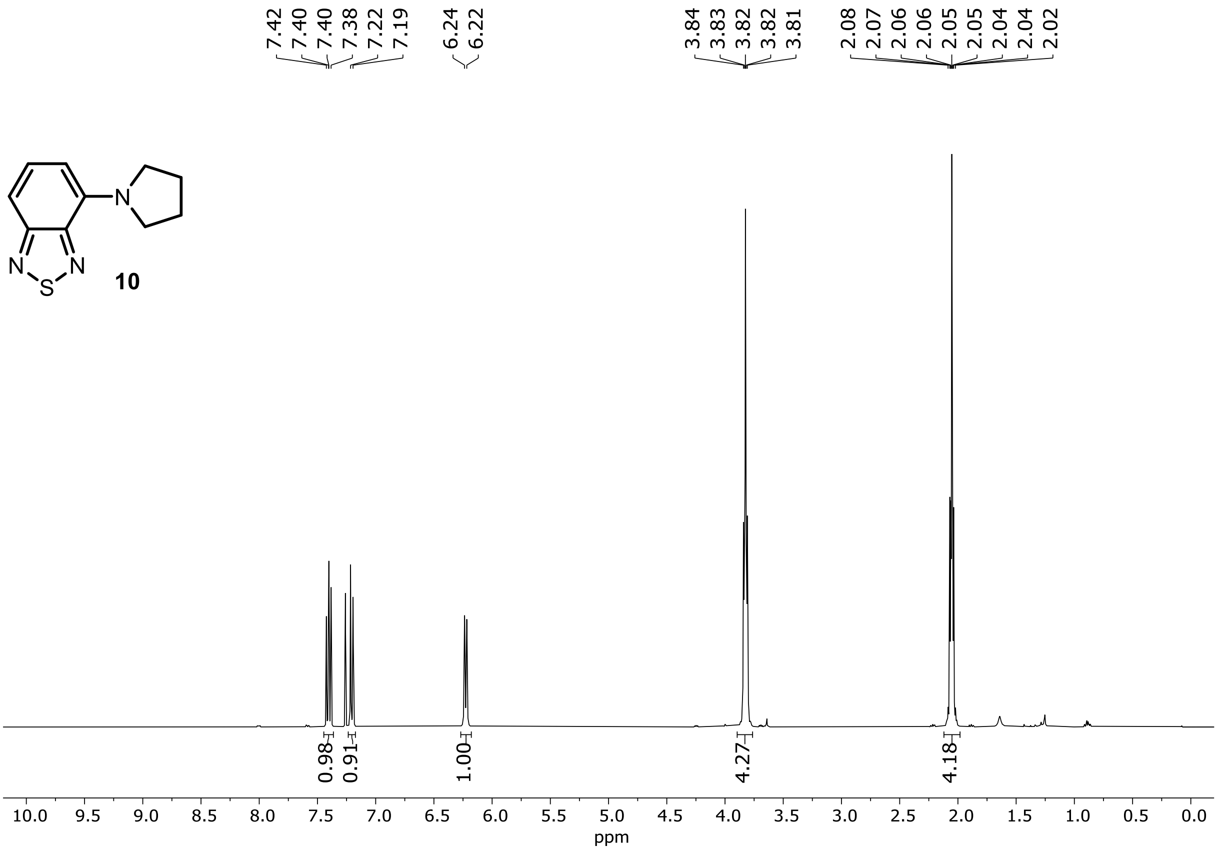

Figure S56. ^{1}H NMR spectrum (400 MHz, $CDCl_3$) of compound **10**.

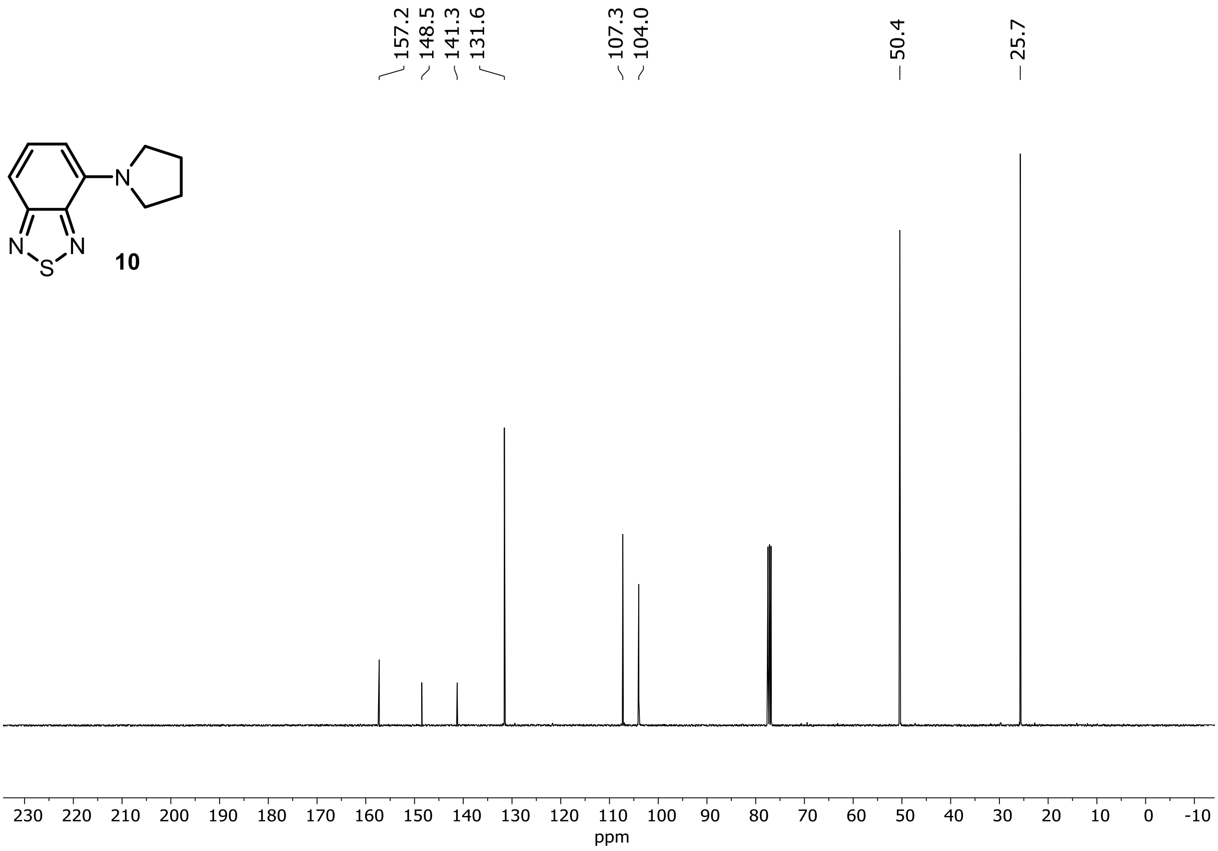

Figure S57. ^{13}C NMR spectrum (101 MHz, $CDCl_3$) of compound **10**.

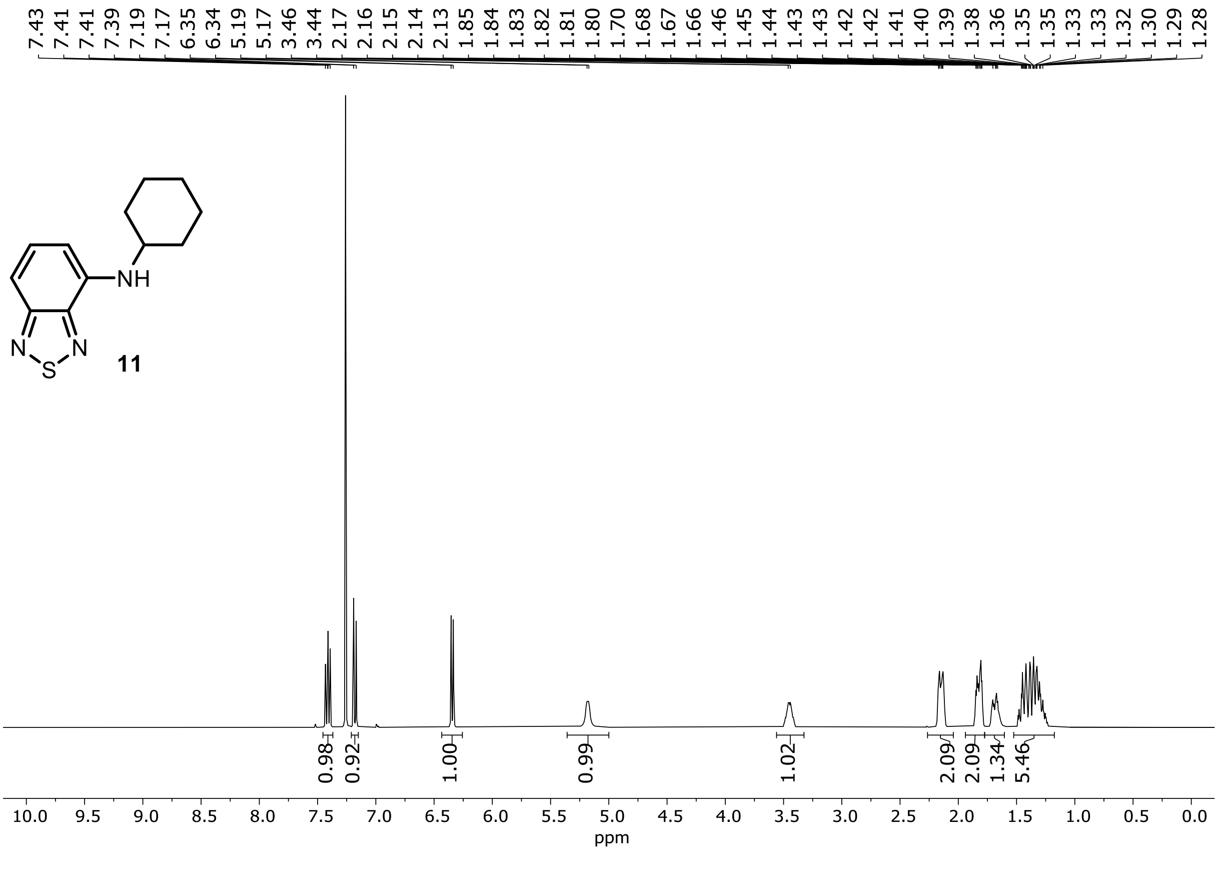

Figure S58. ^{1}H NMR spectrum (400 MHz, $CDCl_3$) of compound **11**.

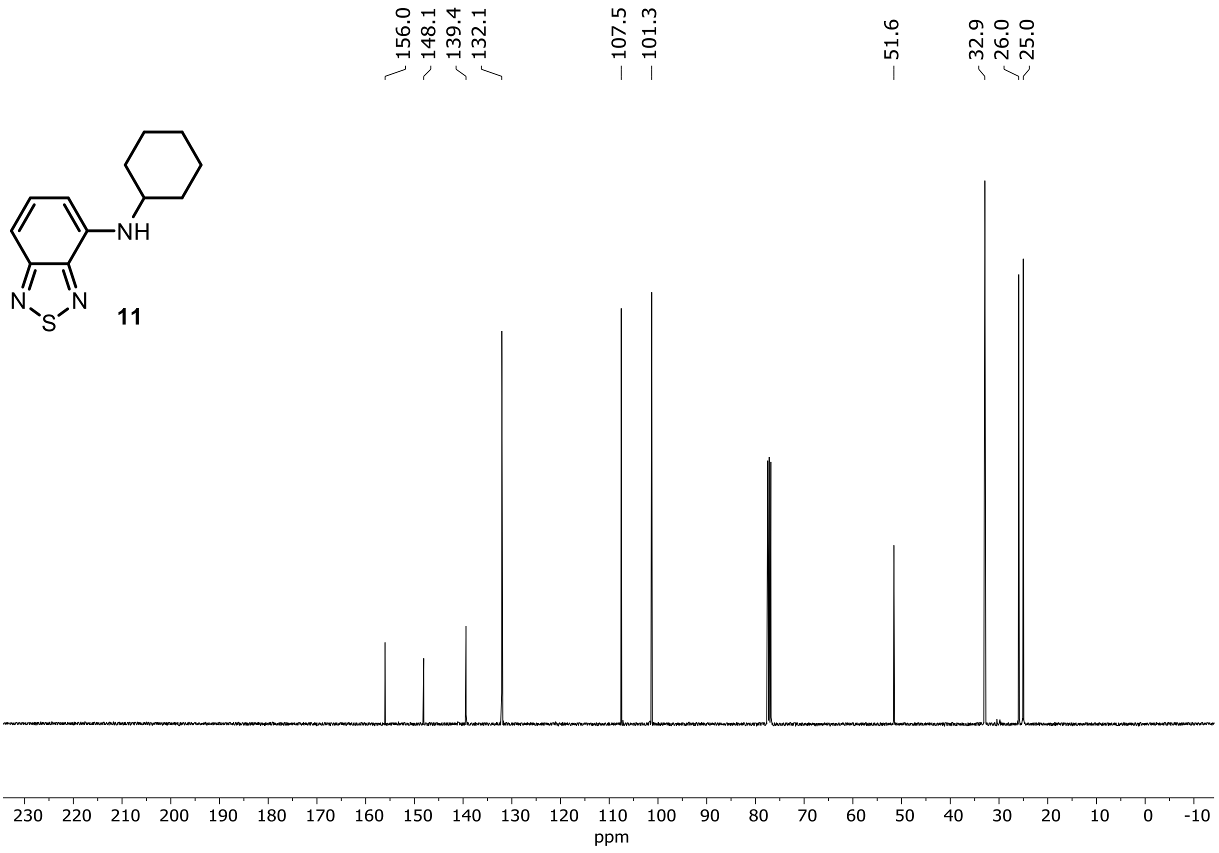

Figure S59. ^{13}C NMR spectrum (101 MHz, $CDCl_3$) of compound **11**.

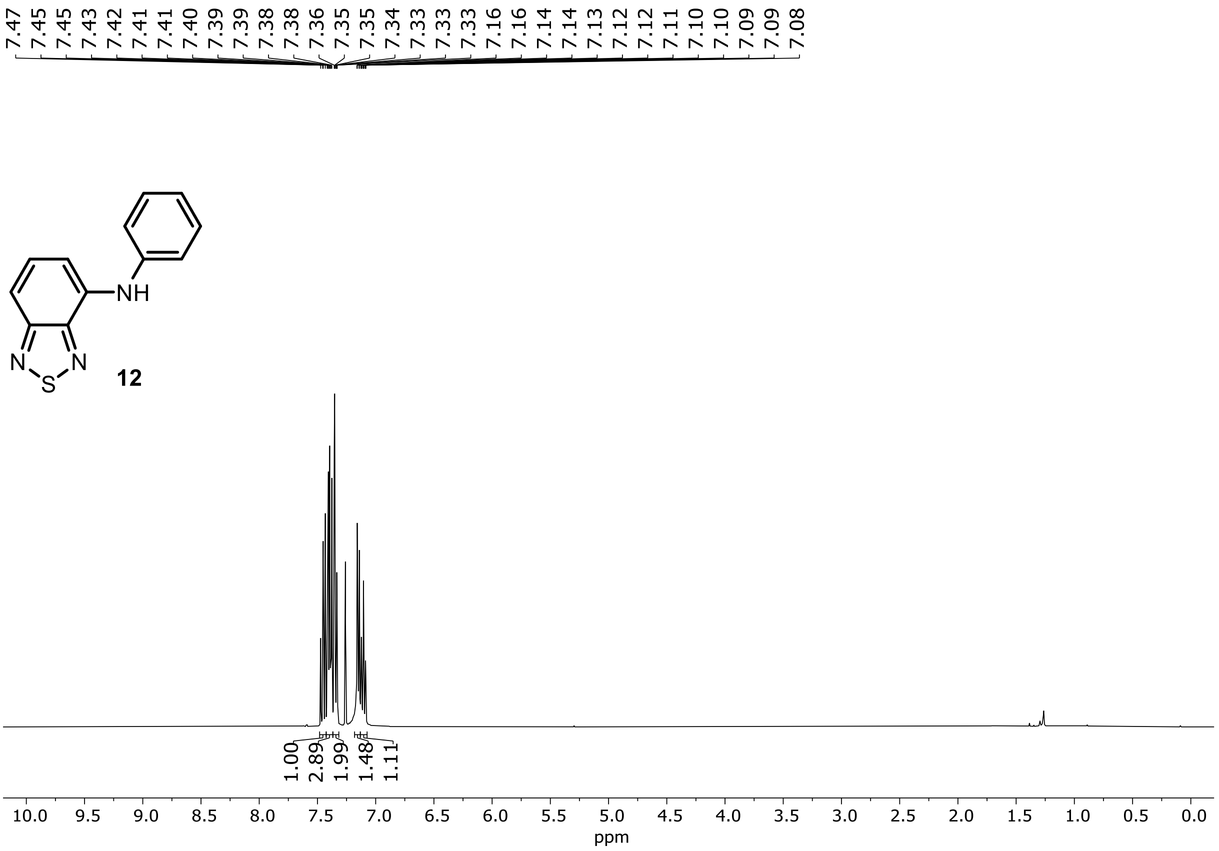

Figure S60. ^{1}H NMR spectrum (400 MHz, $CDCl_3$) of compound **12**.

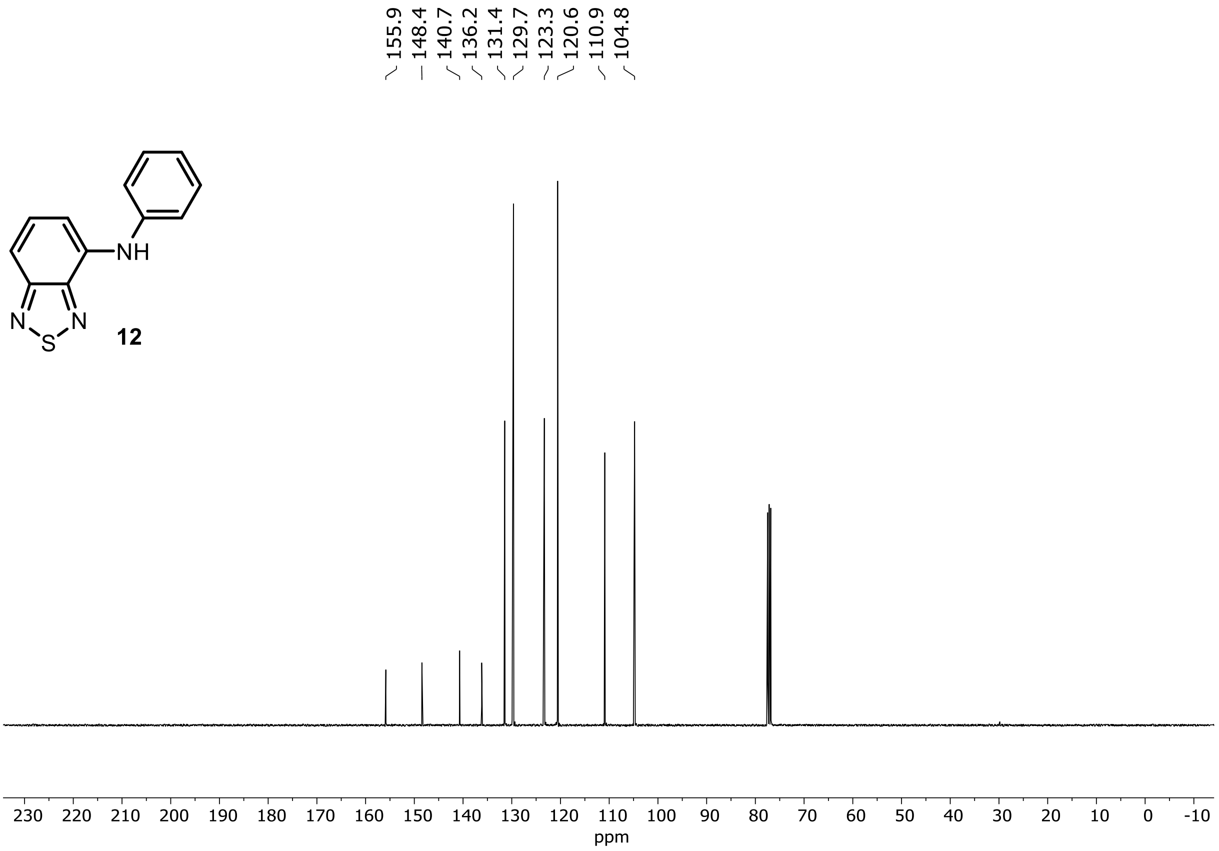

Figure S61. ^{13}C NMR spectrum (101 MHz, $CDCl_3$) of compound **12**.

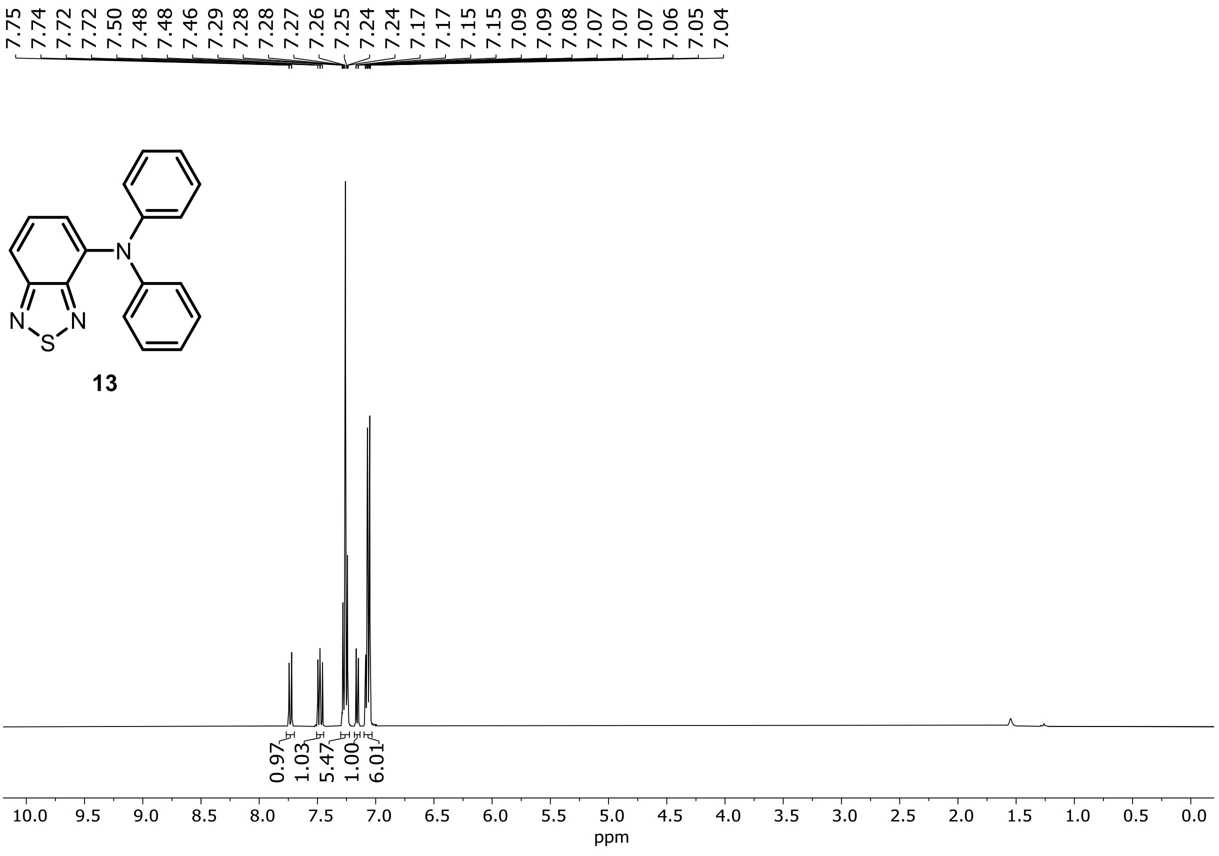

Figure S62. ^{1}H NMR spectrum (400 MHz, $CDCl_3$) of compound **13**.

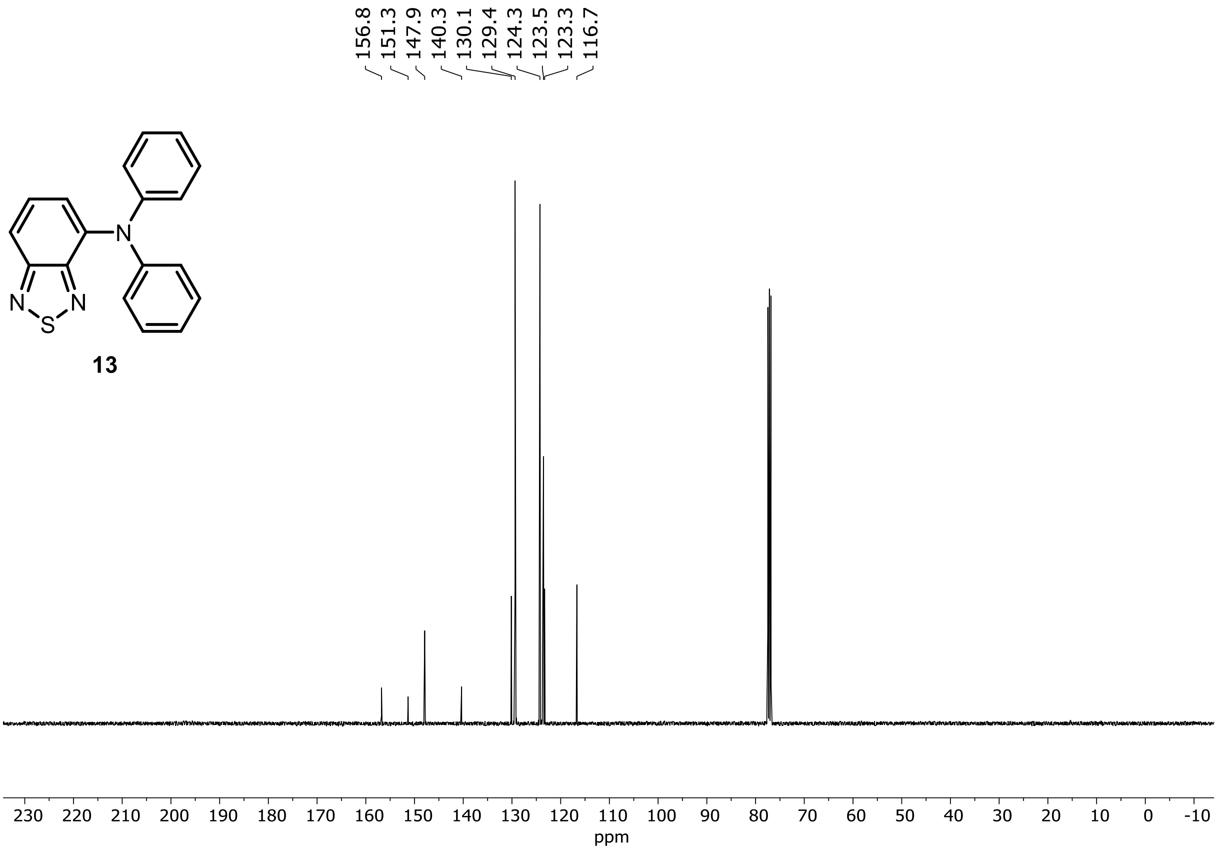

Figure S63. ^{13}C NMR spectrum (101 MHz, $CDCl_3$) of compound **13**.

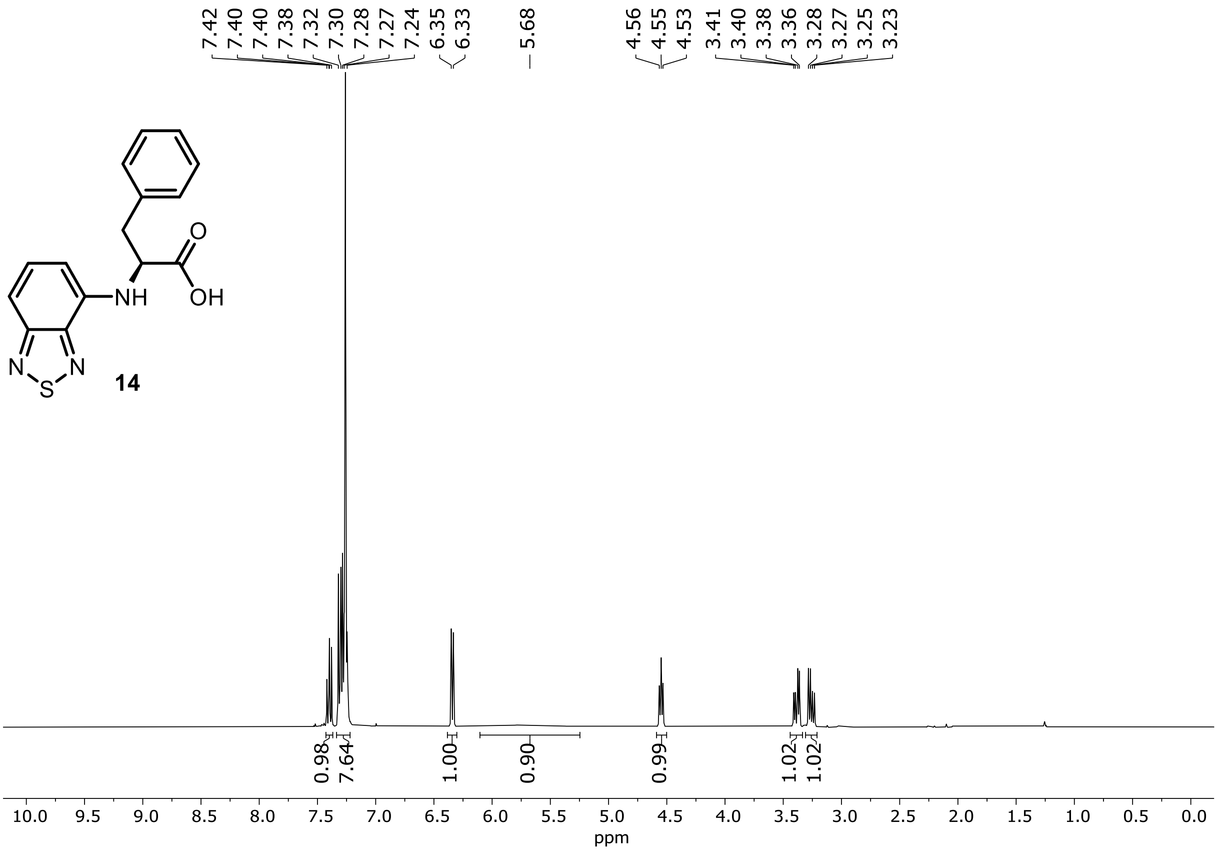

Figure S64. ^{1}H NMR spectrum (400 MHz, $CDCl_3$) of compound **14**.

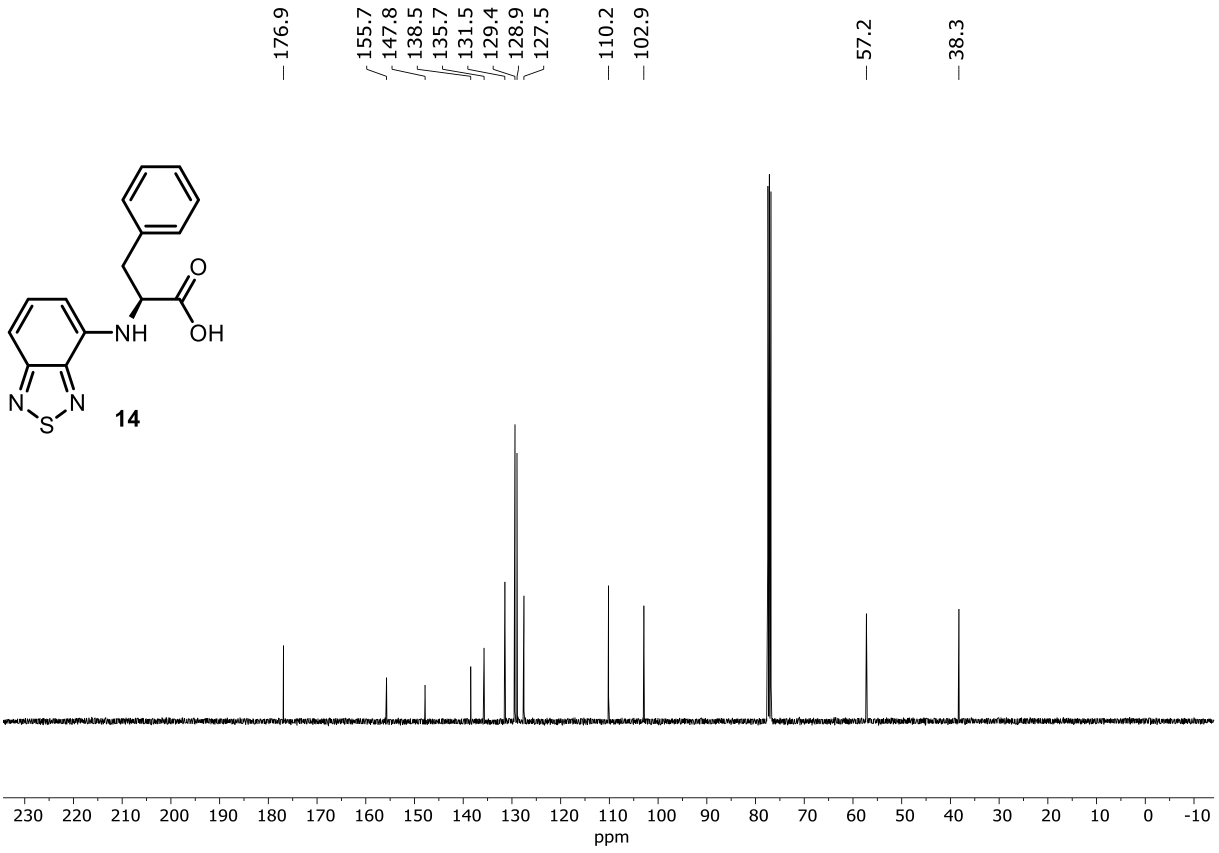

Figure S65. ^{13}C NMR spectrum (101 MHz, $CDCl_3$) of compound **14**.

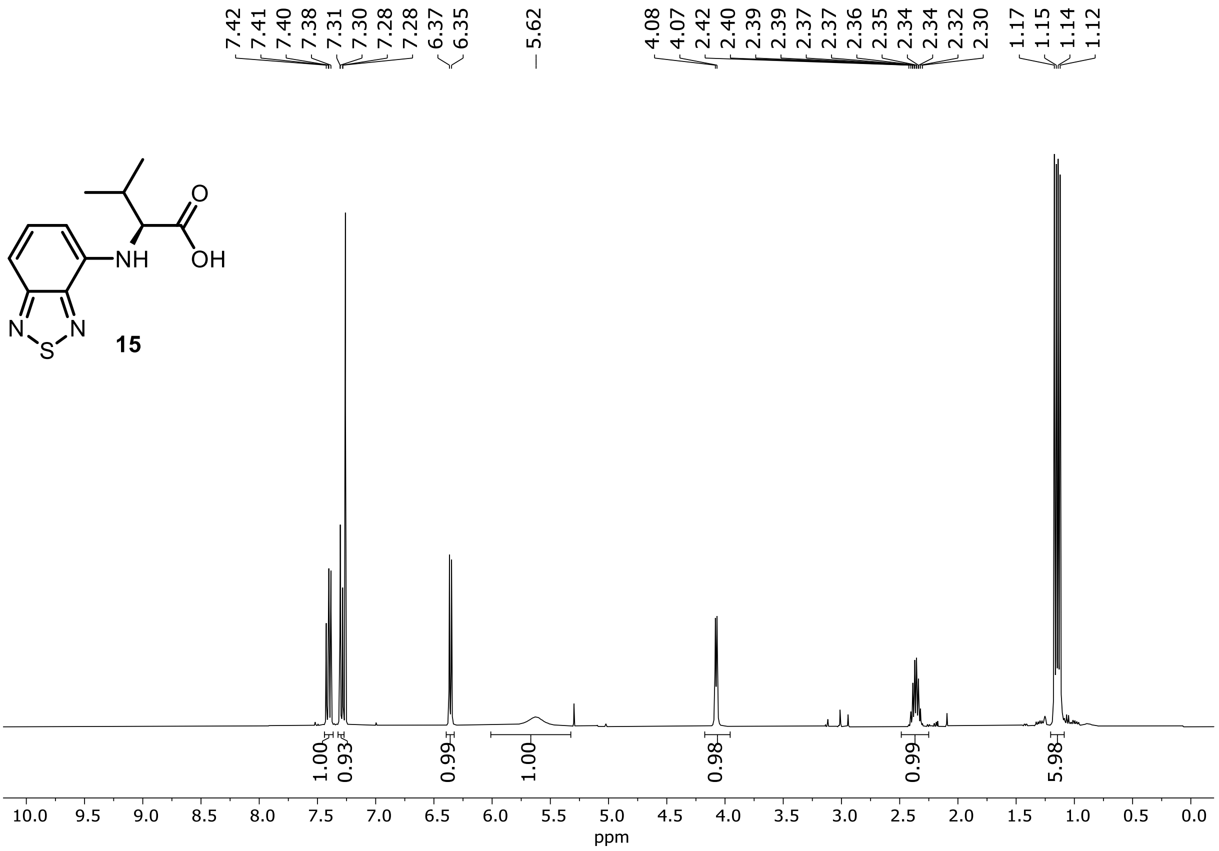

Figure S66. ^{1}H NMR spectrum (400 MHz, $CDCl_3$) of compound **15**.

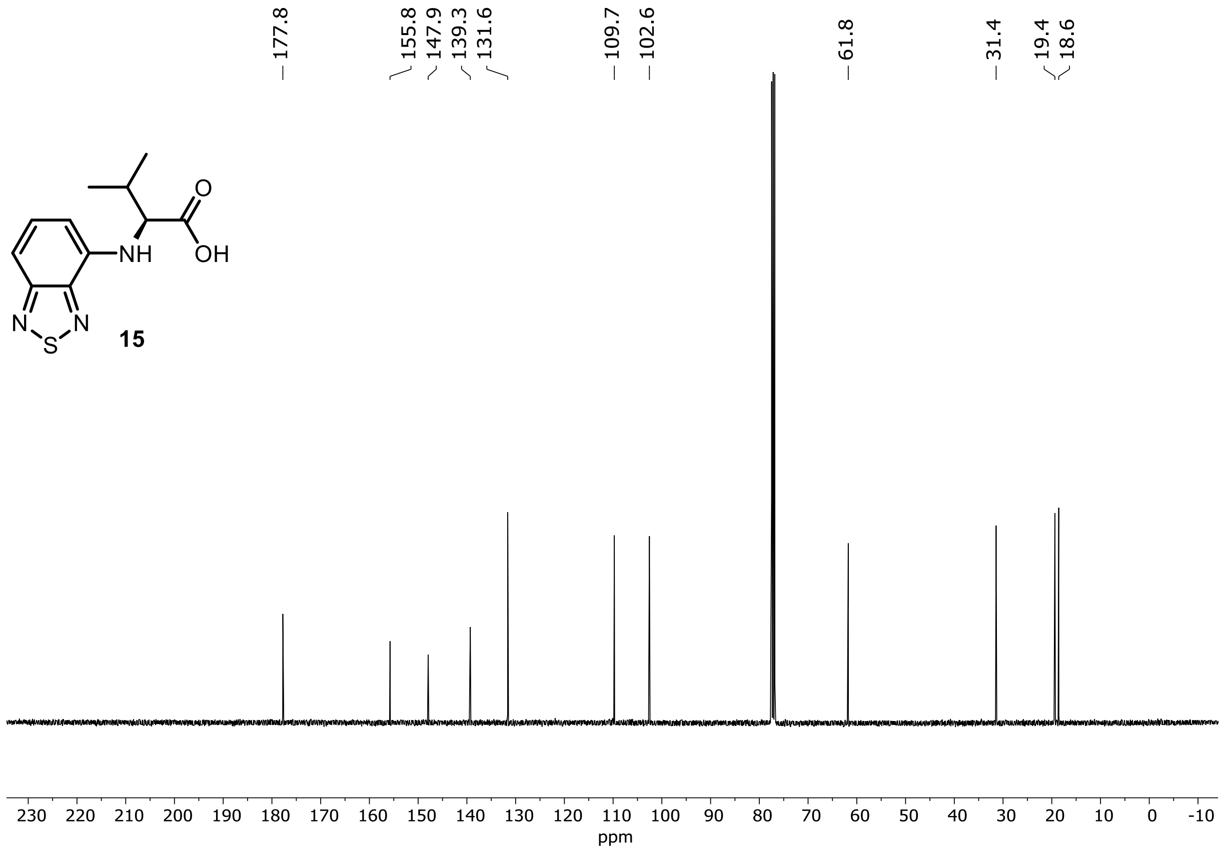

Figure S67. ^{13}C NMR spectrum (101 MHz, $CDCl_3$) of compound **15**.

VIII. Optimized structures from computations

Cartesian coordinates and energies of optimized structures of data reported in Table S2.

Compound, solvent	**1**, *n*-hexane	
State	Ground state (S0)	Excited state (S1)
Cartesian coordinates (Å)	C -0.640942 1.984040 -0.000723<br>C -1.937059 1.560792 0.006645<br>C -2.314744 0.184704 0.005423<br>C -1.380298 -0.820579 -0.008534<br>C -0.000476 -0.414383 -0.008512<br>C 0.366461 0.978458 -0.005230<br>N 1.686159 1.159404 0.000693<br>N 1.032334 -1.247323 -0.000556<br>S 2.374728 -0.319129 0.004541<br>H -3.369909 -0.069023 0.008187<br>H -0.366393 3.030906 0.000549<br>H -2.734341 2.297439 0.014511<br>N -1.661100 -2.161770 -0.063068<br>H -2.578691 -2.460038 0.227437<br>H -0.905697 -2.783590 0.182751	C -0.715658 1.914849 0.000043<br>C -2.054549 1.489177 -0.000039<br>C -2.374053 0.146398 -0.000191<br>C -1.333567 -0.832097 -0.000044<br>C 0.028049 -0.413946 -0.000391<br>C 0.343334 0.962101 0.000070<br>N 1.649433 1.244831 0.000444<br>N 1.057177 -1.270442 -0.000459<br>S 2.427970 -0.259669 -0.000009<br>H -3.406858 -0.184758 -0.000416<br>H -0.474709 2.971495 0.000096<br>H -2.847666 2.227509 -0.000202<br>N -1.586485 -2.141915 0.000364<br>H -2.524373 -2.511543 0.000873<br>H -0.796117 -2.774215 0.000657
Energy (E_h)	-793.876796673	-793.767935275<br>State-specific equilibrium solvation (External Iteration approach):<br>-793.775655783
Zero-point correction<br><u>Thermal corrections to:</u><br>Energy<br>Enthalpy<br>Gibbs Free Energy	0.108983<br><br>0.116427<br>0.117371<br>0.076799	0.106917<br><br>0.114787<br>0.115731<br>0.074313

Compound, solvent	**2**, *n*-hexane	
State	Ground state (S0)	Excited state (S1)
Cartesian coordinates (Å)	C 0.576358 2.194338 0.007584<br>C -0.781000 2.321939 0.000345<br>C -1.681806 1.214798 -0.013564<br>C 1.099735 0.869863 0.002390<br>C 0.207130 -0.260645 -0.015324<br>C -1.223896 -0.083190 -0.028024<br>N 2.380751 0.508178 0.015644<br>N 0.819085 -1.435783 -0.012848<br>S 2.423140 -1.123551 0.008271<br>H -2.746640 1.412480 -0.016077<br>H 1.245096 3.045111 0.019491<br>H -1.216379 3.316526 0.007262<br>N -2.001735 -1.198386 -0.063745<br>C -3.436863 -1.154497 0.048906<br>H -3.765062 -0.681747 0.984114<br>H -3.826529 -2.171965 0.021400<br>H -3.881254 -0.599166 -0.784744<br>H -1.524130 -2.078122 0.048988	C 0.485203 2.175427 0.000017<br>C -0.909865 2.325697 -0.000006<br>C -1.749120 1.226758 -0.000007<br>C 1.068180 0.875899 0.000014<br>C 0.221742 -0.252402 -0.000005<br>C -1.196226 -0.088906 0.000009<br>N 2.377622 0.603398 -0.000010<br>N 0.815131 -1.452646 0.000004<br>S 2.478089 -1.085239 -0.000008<br>H -2.824462 1.355960 -0.000039<br>H 1.132407 3.044764 0.000012<br>H -1.337067 3.321607 -0.000036<br>N -1.934961 -1.201748 0.000048<br>C -3.379663 -1.270187 -0.000021<br>H -3.798875 -0.789541 0.889294<br>H -3.677795 -2.317102 0.000353<br>H -3.798797 -0.790192 -0.889732<br>H -1.390880 -2.058410 -0.000028
Energy (E_h)	-833.161872025	-833.054889767<br>State-specific equilibrium solvation (External Iteration approach):<br>-833.062646485
Zero-point correction<br><u>Thermal corrections to:</u><br>Energy<br>Enthalpy<br>Gibbs Free Energy	0.137278<br><br>0.146444<br>0.147389<br>0.102418	0.135492<br><br>0.144952<br>0.145896<br>0.100479

Compound, solvent	**3**, *n*-hexane	
State	Ground state (S0)	Excited state (S1)
Cartesian coordinates (Å)	C -1.123899 2.213556 0.139597<br>C 0.187693 2.569822 0.121813<br>C 1.249229 1.619543 0.041247<br>C -1.412715 0.823043 0.055531<br>C -0.351196 -0.156342 -0.036871<br>C 1.037565 0.261100 -0.036957<br>N -2.627895 0.282524 0.023038<br>N -0.803908 -1.400173 -0.149813<br>S -2.431542 -1.327676 -0.122890<br>H 2.260640 2.002439 0.055542<br>H -1.930918 2.932089 0.202808<br>H 0.458642 3.619178 0.181848<br>N 2.063596 -0.660552 -0.193914<br>C 3.397407 -0.136811 -0.402885<br>H 3.394338 0.599243 -1.209437<br>H 4.051934 -0.960078 -0.695257<br>H 3.823845 0.333225 0.497459<br>C 2.055184 -1.891233 0.589423<br>H 1.053389 -2.307250 0.635391<br>H 2.426433 -1.722917 1.611556<br>H 2.708198 -2.621780 0.105778	C -1.017491 2.225058 0.049682<br>C 0.326679 2.596548 0.049420<br>C 1.320016 1.638909 0.021183<br>C -1.367318 0.849341 0.021172<br>C -0.360755 -0.158542 -0.001482<br>C 1.026030 0.233966 -0.003344<br>N -2.628980 0.411014 -0.001034<br>N -0.832479 -1.416969 -0.049972<br>S -2.519698 -1.268665 -0.052591<br>H 2.350668 1.961410 0.048857<br>H -1.807435 2.966530 0.069743<br>H 0.599867 3.644806 0.079587<br>N 2.058030 -0.640219 -0.028199<br>C 3.434832 -0.180432 -0.173413<br>H 3.529162 0.534396 -0.991698<br>H 4.057339 -1.043840 -0.402371<br>H 3.808179 0.276221 0.749837<br>C 1.940871 -2.074356 0.209977<br>H 0.897439 -2.355944 0.308787<br>H 2.495097 -2.328326 1.121101<br>H 2.391667 -2.616343 -0.627118
Energy (E_h)	-872.437903341	-872.331402258<br>State-specific equilibrium solvation (External Iteration approach):<br>-872.338465059
Zero-point correction<br><u>Thermal corrections to:</u><br>Energy<br>Enthalpy<br>Gibbs Free Energy	0.166220<br><br>0.176349<br>0.177293<br>0.130336	0.163897<br><br>0.174652<br>0.175596<br>0.127172

Compound, solvent	**4**, *n*-hexane	
State	Ground state (S0)	Excited state (S1)
Cartesian coordinates (Å)	C 0.373641 2.555765 -0.065973<br>C 1.659821 2.119188 0.007455<br>C 1.864207 0.710343 0.020171<br>C 0.737572 -0.180454 -0.030941<br>C -0.611360 0.317998 -0.098168<br>C -0.756832 1.679312 -0.127078<br>N 3.027688 0.065813 0.074713<br>N 1.066361 -1.465687 -0.015843<br>S 2.694108 -1.528435 0.058256<br>H -1.733028 2.132174 -0.208080<br>H 0.173879 3.621976 -0.086207<br>H 2.505360 2.793647 0.050252<br>N -1.590740 -0.667846 -0.150250<br>C -2.964920 -0.667283 -0.000578<br>H -1.225129 -1.608676 -0.251568<br>C -3.681032 0.627727 0.277213<br>O -3.558260 -1.726764 -0.088949<br>H -3.221336 1.175621 1.102469<br>H -4.711524 0.382461 0.527089<br>H -3.677612 1.270330 -0.607395	C 0.255169 2.588878 0.000014<br>C 1.585173 2.118054 -0.000062<br>C 1.844546 0.728786 -0.000019<br>C 0.747733 -0.172649 0.000007<br>C -0.595802 0.314764 -0.000007<br>C -0.819698 1.727698 0.000086<br>N 3.047691 0.146256 -0.000145<br>N 1.052021 -1.468968 0.000106<br>S 2.750478 -1.511693 0.000042<br>H -1.820829 2.127530 0.000238<br>H 0.075151 3.657372 0.000044<br>H 2.414721 2.815699 -0.000189<br>N -1.538322 -0.661961 0.000054<br>C -2.942373 -0.708912 -0.000052<br>H -1.129790 -1.598557 -0.000022<br>C -3.743044 0.561189 0.000030<br>O -3.451238 -1.809110 -0.000120<br>H -3.531373 1.161446 0.888066<br>H -4.794390 0.280709 -0.000101<br>H -3.531197 1.161640 -0.887836
Energy (E_h)	-946.467777082	-946.349404422<br>State-specific equilibrium solvation (External Iteration approach):<br>-946.354252918
Zero-point correction<br><u>Thermal corrections to:</u><br>Energy<br>Enthalpy<br>Gibbs Free Energy	0.148001<br><br>0.158554<br>0.159499<br>0.109468	0.145675<br><br>0.156630<br>0.157574<br>0.108051

Compound, solvent	**1**, THF (implicit solvation and one explicit THF molecule)	
State	Ground state (S0)	Excited state (S1)
Cartesian coordinates (Å)	C 2.670632 2.093533 -0.021778<br>C 1.345029 2.416095 -0.089598<br>C 0.298796 1.450916 -0.130885<br>C 0.559739 0.098800 -0.110954<br>C 1.946985 -0.282529 -0.032344<br>C 2.993832 0.708660 0.011875<br>N 4.208657 0.165428 0.087733<br>N 2.385652 -1.533826 0.012782<br>S 4.013678 -1.455756 0.103006<br>H -0.733206 1.781147 -0.177072<br>H 3.453789 2.840073 0.009682<br>H 1.058674 3.463336 -0.111658<br>N -0.391061 -0.874862 -0.194739<br>H -1.357058 -0.603288 -0.022763<br>H -0.111715 -1.806535 0.070173<br>C -4.996170 -0.830637 -0.660797<br>C -3.802215 -0.012829 -1.131213<br>O -3.046248 0.276001 0.053743<br>C -3.870343 0.097238 1.218739<br>C -5.277330 -0.199412 0.704017<br>H -4.716046 -1.882377 -0.547949<br>H -5.841608 -0.772677 -1.348855<br>H -4.122904 0.927756 -1.596511<br>H -3.147951 -0.540484 -1.829487<br>H -3.818181 1.001886 1.830682<br>H -3.469392 -0.738651 1.803146<br>H -5.843503 0.728556 0.581478<br>H -5.836216 -0.850853 1.378284	C 2.535864 2.086840 -0.071998<br>C 1.173987 2.427556 -0.063912<br>C 0.196200 1.454906 -0.014152<br>C 0.560606 0.071419 0.030653<br>C 1.942211 -0.288787 0.022359<br>C 2.934298 0.720097 -0.028776<br>N 4.196067 0.276590 -0.031803<br>N 2.373926 -1.555597 0.061045<br>S 4.072931 -1.415613 0.030335<br>H -0.859015 1.703079 -0.008231<br>H 3.295745 2.858550 -0.111369<br>H 0.887298 3.472014 -0.097486<br>N -0.364879 -0.880633 0.080017<br>H -1.364593 -0.642774 0.086026<br>H -0.053491 -1.842401 0.110274<br>C -4.970942 -0.794899 -0.730506<br>C -3.720223 -0.052978 -1.174837<br>O -2.963011 0.166511 0.028469<br>C -3.819925 0.035931 1.181546<br>C -5.232552 -0.160838 0.637010<br>H -4.762406 -1.863846 -0.626372<br>H -5.799539 -0.673033 -1.430317<br>H -3.968295 0.915603 -1.624820<br>H -3.092789 -0.612092 -1.872683<br>H -3.715628 0.931639 1.798803<br>H -3.484806 -0.829253 1.763547<br>H -5.733768 0.803823 0.516772<br>H -5.844468 -0.781603 1.293610
Energy (E_h)	-1026.23190156	-1026.13129932<br>State-specific equilibrium solvation (External Iteration approach):<br>-1026.14822248
Zero-point correction<br><u>Thermal corrections to:</u><br>Energy<br>Enthalpy<br>Gibbs Free Energy	0.228551<br><br>0.242786<br>0.243730<br>0.182496	0.226939<br><br>0.241359<br>0.242304<br>0.181591

Compound, solvent	**2**, THF (implicit solvation and one explicit THF molecule)	
State	Ground state (S0)	Excited state (S1)
Cartesian coordinates (Å)	C 3.749905 -0.303143 -0.081769<br>C 3.715984 1.061685 -0.089257<br>C 2.515453 1.825793 -0.037601<br>C 2.497462 -0.975299 -0.018231<br>C 1.267175 -0.221904 0.033983<br>C 1.273061 1.224073 0.023625<br>N 2.292515 -2.291242 0.001340<br>N 0.178285 -0.974509 0.090829<br>S 0.678029 -2.530285 0.079677<br>H 2.588892 2.906105 -0.047511<br>H 4.673056 -0.866751 -0.121062<br>H 4.652079 1.610200 -0.136504<br>N 0.090582 1.877982 0.069993<br>C -0.004359 3.314989 0.091324<br>H 0.506975 3.748430 0.960443<br>H -1.058986 3.585359 0.140910<br>H 0.427225 3.766389 -0.810656<br>H -0.778213 1.352541 0.145741<br>C -3.419121 -0.927350 -0.912468<br>C -3.004290 0.519773 -1.144202<br>O -2.646694 1.038329 0.145421<br>C -3.090570 0.142088 1.177316<br>C -4.018011 -0.856177 0.493109<br>H -2.537521 -1.573952 -0.917221<br>H -4.120404 -1.287658 -1.667623<br>H -3.832133 1.114461 -1.550435<br>H -2.142223 0.625024 -1.808230<br>H -3.582564 0.728502 1.958138<br>H -2.215680 -0.357756 1.607143<br>H -5.039523 -0.465808 0.453105<br>H -4.041703 -1.819954 1.005457	C 3.685113 -0.295384 0.028304<br>C 3.711597 1.105766 0.016823<br>C 2.542452 1.841918 0.001082<br>C 2.440824 -0.985432 0.023795<br>C 1.232859 -0.244364 0.007802<br>C 1.270827 1.187978 -0.003586<br>N 2.294532 -2.315777 0.033129<br>N 0.098552 -0.956365 0.003742<br>S 0.620903 -2.579129 0.022046<br>H 2.580646 2.923828 -0.007400<br>H 4.607061 -0.864960 0.040463<br>H 4.664776 1.621065 0.020396<br>N 0.119931 1.856299 -0.018346<br>C -0.018288 3.297379 -0.032734<br>H 0.424202 3.752219 0.858874<br>H -1.083075 3.523510 -0.050464<br>H 0.450051 3.736498 -0.918850<br>H -0.762443 1.323377 -0.017001<br>C -3.451505 -1.038346 -0.672746<br>C -2.900011 0.280220 -1.203157<br>O -2.545937 1.060585 -0.045789<br>C -2.985920 0.393269 1.149954<br>C -4.015407 -0.628219 0.688615<br>H -2.635844 -1.753431 -0.542267<br>H -4.197719 -1.474970 -1.339369<br>H -3.650816 0.836553 -1.776225<br>H -2.006964 0.148830 -1.818647<br>H -3.387026 1.147359 1.831837<br>H -2.124820 -0.096150 1.617966<br>H -4.996761 -0.157595 0.573990<br>H -4.114580 -1.462558 1.385684
Energy (E_h)	-1065.51680588	-1065.41679682<br>State-specific equilibrium solvation (External Iteration approach):<br>-1065.43065146
Zero-point correction<br>Thermal corrections to:<br>Energy<br>Enthalpy<br>Gibbs Free Energy	0.257068<br><br>0.272726<br>0.273670<br>0.210458	0.255258<br><br>0.271201<br>0.272146<br>0.208796

Compound, solvent	**3**, THF	
State	Ground state (S0)	Excited state (S1)
Cartesian coordinates (Å)	C -1.121088 2.213550 0.147858<br>C 0.191460 2.568644 0.123648<br>C 1.251769 1.617478 0.036487<br>C -1.412006 0.823664 0.060453<br>C -0.351788 -0.155765 -0.039555<br>C 1.037345 0.259443 -0.040764<br>N -2.627866 0.282776 0.028354<br>N -0.805804 -1.398746 -0.160451<br>S -2.433129 -1.327414 -0.128300<br>H 2.263776 1.998992 0.045985<br>H -1.925465 2.934738 0.217124<br>H 0.463632 3.617544 0.184447<br>N 2.059149 -0.667481 -0.199771<br>C 3.394176 -0.146308 -0.419469<br>H 3.388181 0.583979 -1.231006<br>H 4.045941 -0.972908 -0.708024<br>H 3.823783 0.329272 0.475764<br>C 2.058268 -1.877049 0.618454<br>H 1.056529 -2.290031 0.689413<br>H 2.441119 -1.679127 1.630507<br>H 2.705397 -2.621636 0.148996	C -1.020518 2.225201 0.054512<br>C 0.326401 2.594707 0.052635<br>C 1.318790 1.638738 0.021709<br>C -1.370432 0.852076 0.024083<br>C -0.360950 -0.160101 -0.001837<br>C 1.027703 0.232291 -0.002588<br>N -2.632060 0.410533 0.000308<br>N -0.832742 -1.415510 -0.056898<br>S -2.522921 -1.272034 -0.057151<br>H 2.349093 1.961576 0.048429<br>H -1.807085 2.970213 0.076275<br>H 0.599895 3.642628 0.084117<br>N 2.059159 -0.639020 -0.026703<br>C 3.435940 -0.178478 -0.189227<br>H 3.518075 0.537627 -1.006878<br>H 4.054123 -1.042052 -0.427637<br>H 3.817789 0.274672 0.731508<br>C 1.951050 -2.070469 0.227869<br>H 0.914233 -2.353906 0.368812<br>H 2.540215 -2.313357 1.119181<br>H 2.371979 -2.620666 -0.619280
Energy (E_h)	-872.440426992	-872.337590324<br>State-specific equilibrium solvation (External Iteration approach):<br>-872.353809143
Zero-point correction<br>Thermal corrections to:<br>Energy<br>Enthalpy<br>Gibbs Free Energy	0.166169<br><br>0.176272<br>0.177216<br>0.130372	0.163924<br><br>0.174662<br>0.175606<br>0.127318

Compound, solvent	**4**, THF (implicit solvation and one explicit THF molecule)	
State	Ground state (S0)	Excited state (S1)
Cartesian coordinates (Å)	C 3.878149 0.447362 0.567368<br>C 3.339309 1.678780 0.778678<br>C 1.944484 1.950053 0.614720<br>C 2.995071 -0.591452 0.156685<br>C 1.593099 -0.322551 -0.036014<br>C 1.062012 0.992391 0.196850<br>N 3.320981 -1.856981 -0.100355<br>N 0.898266 -1.381190 -0.437374<br>S 1.952116 -2.618526 -0.547845<br>H 1.585004 2.943740 0.849542<br>H 4.932763 0.242076 0.702094<br>H 3.976461 2.495312 1.100881<br>N -0.311331 1.183314 0.017495<br>C -0.974808 2.255553 -0.518087<br>H -0.929701 0.404473 0.288039<br>C -0.187704 3.388106 -1.132930<br>H -0.812010 3.831488 -1.907943<br>H 0.764683 3.068305 -1.555560<br>H 0.011144 4.158916 -0.383490<br>O -2.200846 2.273968 -0.517406<br>C -4.368657 -0.301948 0.178905<br>C -3.353360 -0.146071 1.299425<br>O -2.174924 -0.828290 0.842396<br>C -2.514720 -1.735137 -0.221776<br>C -4.036408 -1.700206 -0.343679<br>H -4.173157 0.454259 -0.585904<br>H -5.399472 -0.199127 0.524148<br>H -3.701436 -0.612803 2.230102<br>H -3.086724 0.894293 1.491604<br>H -2.122891 -2.726503 0.022314<br>H -2.023371 -1.391528 -1.137810<br>H -4.490623 -2.462948 0.296190<br>H -4.372600 -1.874254 -1.367829	C -3.867725 0.427856 -0.383912<br>C -3.378353 1.743850 -0.523210<br>C -2.040700 2.027250 -0.386251<br>C -2.975059 -0.632522 -0.122093<br>C -1.576519 -0.359849 0.018253<br>C -1.099617 0.984339 -0.096914<br>N -3.311660 -1.916189 0.036611<br>N -0.810496 -1.409253 0.295742<br>S -1.870114 -2.734963 0.342152<br>H -1.686184 3.032706 -0.556414<br>H -4.926993 0.221212 -0.484924<br>H -4.069286 2.543214 -0.762604<br>N 0.239670 1.187065 0.003403<br>C 0.964227 2.339209 0.334768<br>H 0.861228 0.368495 -0.179487<br>C 0.269985 3.520740 0.957134<br>H 0.994642 4.009340 1.607593<br>H -0.615934 3.245353 1.528226<br>H -0.016337 4.240252 0.185064<br>O 2.169119 2.325643 0.160053<br>C 4.387006 -0.353108 -0.206246<br>C 3.300084 -0.246275 -1.262723<br>O 2.122757 -0.782970 -0.642396<br>C 2.492353 -1.645288 0.449927<br>C 4.020717 -1.674066 0.472879<br>H 4.295710 0.482026 0.494105<br>H 5.393106 -0.346613 -0.630442<br>H 3.543977 -0.842413 -2.151736<br>H 3.082291 0.779605 -1.560224<br>H 2.041989 -2.628101 0.291755<br>H 2.074901 -1.227085 1.371229<br>H 4.397018 -2.517134 -0.114239<br>H 4.415682 -1.766995 1.486453
Energy (E_h)	-1178.82553823	-1178.70841306<br>State-specific equilibrium solvation (External Iteration approach):<br>-1178.71890088
Zero-point correction<br><u>Thermal corrections to:</u><br>Energy<br>Enthalpy<br>Gibbs Free Energy	0.267442<br><br>0.284516<br>0.285460<br>0.219702	0.264814<br><br>0.282300<br>0.283244<br>0.216409

Compound, solvent	**1**, water (implicit solvation and two explicit H_2O molecules)
State	Ground state (S0)
Cartesian coordinates (Å)	C -0.823262 2.392815 0.445389<br>C 0.534596 2.489120 0.525611<br>C 1.412566 1.396344 0.255440<br>C 0.940560 0.157705 -0.097171<br>C -0.487139 0.010095 -0.173332<br>C -1.361660 1.125039 0.087120<br>N -2.650234 0.806148 -0.033419<br>N -1.129892 -1.115209 -0.471529<br>S -2.726031 -0.770944 -0.434391<br>H 2.485040 1.546645 0.315542<br>H -1.481193 3.229159 0.644606<br>H 0.984129 3.439097 0.796461<br>N 1.746364 -0.947871 -0.319418<br>H 2.735716 -0.720241 -0.443012<br>H 1.392707 -1.573594 -1.033044<br>H 5.092697 -0.481203 0.026713<br>O 4.478622 0.062827 -0.482479<br>H 4.837155 0.059687 -1.378993<br>H 1.131021 -2.446023 0.969905<br>O 0.479627 -3.124519 1.216845<br>H -0.284404 -2.853077 0.689359
Energy (E_h)	-946.698308665
Zero-point correction<br><u>Thermal corrections to:</u><br>Energy<br>Enthalpy<br>Gibbs Free Energy	0.157702<br><br>0.172070<br>0.173014<br>0.114822

Compound, solvent	**2**, water (implicit solvation and two explicit H_2O molecules)
State	Ground state (S0)
Cartesian coordinates (Å)	C 1.624988 -2.272905 -0.134704<br>C 0.384159 -2.773671 -0.389380<br>C -0.780809 -1.956870 -0.505844<br>C 1.731776 -0.861803 0.014761<br>C 0.565433 -0.021173 -0.096600<br>C -0.734290 -0.590317 -0.359654<br>N 2.844451 -0.170914 0.258166<br>N 0.833563 1.270771 0.063935<br>S 2.439725 1.403010 0.335321<br>H -1.727035 -2.442284 -0.708497<br>H 2.503806 -2.898912 -0.048516<br>H 0.258308 -3.844289 -0.514343<br>N -1.835931 0.236450 -0.397051<br>C -3.099608 -0.267600 -0.905897<br>H -2.998895 -0.735154 -1.893075<br>H -3.795702 0.567569 -0.982115<br>H -3.525716 -0.998642 -0.213420<br>H -1.651892 1.219446 -0.606415<br>H -0.382578 2.747255 -0.414643<br>O -1.222829 3.081318 -0.780099<br>H -1.034717 3.217374 -1.717417<br>H -2.446341 -0.848590 2.581393<br>O -2.882930 -0.025145 2.334096<br>H -2.433243 0.230554 1.508507
Energy (E_h)	-985.984289092
Zero-point correction<br>Thermal corrections to:<br>Energy<br>Enthalpy<br>Gibbs Free Energy	0.187155<br><br>0.202367<br>0.203312<br>0.143612

Compound, solvent	**3**, water (implicit solvation and one explicit H_2O molecule)
State	Ground state (S0)
Cartesian coordinates (Å)	C 1.746712 2.141255 0.204461<br>C 0.545497 2.773908 0.268748<br>C -0.699138 2.081105 0.141402<br>C 1.726767 0.728688 0.029103<br>C 0.478070 0.014149 -0.085482<br>C -0.778514 0.725630 -0.046648<br>N 2.791574 -0.069832 -0.011386<br>N 0.642037 -1.302310 -0.190656<br>S 2.246729 -1.594402 -0.167692<br>H -1.605171 2.670844 0.185850<br>H 2.690372 2.664447 0.294326<br>H 0.510618 3.849011 0.409133<br>N -1.987769 0.010486 -0.130919<br>C -3.193977 0.754659 0.206536<br>H -3.082503 1.228448 1.183096<br>H -4.028690 0.053366 0.255199<br>H -3.439600 1.526406 -0.537017<br>C -2.173162 -0.778867 -1.356023<br>H -1.276396 -1.352707 -1.576355<br>H -2.409612 -0.133688 -2.212639<br>H -2.997759 -1.477051 -1.201840<br>H -1.909160 -1.622231 1.042025<br>O -1.699193 -2.545631 1.270431<br>H -0.840642 -2.642934 0.835994
Energy (E_h)	-948.851056298
Zero-point correction<br><u>Thermal corrections to:</u><br>Energy<br>Enthalpy<br>Gibbs Free Energy	0.191149<br><br>0.204213<br>0.205157<br>0.151745

Compound, solvent	**4**, water (implicit solvation and one explicit H_2O molecule)	
State	Ground state (S0)	Excited state (S1)
Cartesian coordinates (Å)	C 1.108381 2.643926 -0.441776<br>C 2.272903 1.966776 -0.249760<br>C 2.184500 0.561693 -0.036123<br>C 0.901576 -0.088167 -0.009203<br>C -0.305792 0.663856 -0.206623<br>C -0.171886 2.003969 -0.439641<br>N 3.195526 -0.283756 0.157481<br>N 0.968683 -1.397750 0.203111<br>S 2.549232 -1.766114 0.351425<br>H -1.042577 2.610753 -0.647566<br>H 1.132941 3.713448 -0.619483<br>H 3.238585 2.456437 -0.261253<br>N -1.502149 -0.060980 -0.187121<br>C -2.748609 0.293033 0.239331<br>H -1.439535 -1.048677 -0.455899<br>C -2.991111 1.652971 0.833265<br>O -3.672481 -0.521307 0.150425<br>H -2.138655 2.017809 1.406491<br>H -3.869213 1.578884 1.473079<br>H -3.208495 2.375563 0.042143<br>H -2.170637 -3.286518 -0.055484<br>O -2.371989 -2.732340 -0.819886<br>H -3.068553 -2.121634 -0.500252	C 0.863066 2.740327 -0.324798<br>C 2.103117 2.073846 -0.195362<br>C 2.134814 0.674141 -0.037626<br>C 0.903851 -0.055548 0.008212<br>C -0.344975 0.638867 -0.094795<br>C -0.328974 2.060537 -0.281311<br>N 3.231659 -0.079753 0.088916<br>N 1.016567 -1.367058 0.173917<br>S 2.687904 -1.667076 0.249979<br>H -1.251437 2.597623 -0.434173<br>H 0.855220 3.812242 -0.480524<br>H 3.031522 2.632151 -0.228085<br>N -1.461476 -0.133740 -0.068258<br>C -2.808977 0.165345 0.145889<br>H -1.309020 -1.150271 -0.203515<br>C -3.236721 1.527492 0.605248<br>O -3.622288 -0.735741 -0.011471<br>H -2.534684 1.968964 1.312492<br>H -4.213715 1.416983 1.072816<br>H -3.347020 2.199434 -0.250243<br>H -1.825201 -3.392958 0.202907<br>O -1.978427 -2.874142 -0.596842<br>H -2.824862 -2.418082 -0.439594
Energy (E_h)	-1022.88925903	-1022.77190569<br>State-specific equilibrium solvation (External Iteration approach):<br>-1022.78670651
Zero-point correction<br>Thermal corrections to:<br>Energy<br>Enthalpy<br>Gibbs Free Energy	0.173141<br><br>0.186425<br>0.187369<br>0.132414	0.170433<br><br>0.184258<br>0.185202<br>0.129422

Cartesian coordinates and energies of optimized structures of data reported in Figure 2.

Solvent, state	*n*-hexane, ground state (S0)	
Compound	**6**	**10**
Cartesian coordinates	C -2.699333 1.815042 0.140814	C -2.294979 1.931446 -0.085755
(Å)	C -1.602033 2.615438 0.195529	C -1.137536 2.650455 -0.088047
	C -0.267828 2.106244 0.159227	C 0.153386 2.056826 -0.047962
	C -2.471267 0.414864 0.024664	C -2.168398 0.517590 -0.031434
	C -1.127784 -0.116645 -0.025081	C -0.864894 -0.114592 0.012355
	C 0.018262 0.765774 0.052958	C 0.343950 0.686218 -0.002290
	N -3.409602 -0.522840 -0.077564	N -3.170489 -0.358147 -0.001110
	N -1.095371 -1.435926 -0.177478	N -0.929441 -1.439097 0.083011
	S -2.640630 -1.950678 -0.233643	S -2.507647 -1.845281 0.083518
	H 0.541122 2.820988 0.234332	H 1.011931 2.714474 -0.064964
	H -3.711208 2.198552 0.171277	H -3.275060 2.389093 -0.116688
	H -1.726873 3.690075 0.283039	H -1.185836 3.734516 -0.126585
	N 1.306549 0.248262 -0.056999	N 1.583960 0.121582 0.053415
	C 2.388060 1.194071 -0.302586	C 2.781398 0.947413 0.154045
	C 1.689497 -0.822540 0.874216	C 1.907212 -1.297610 -0.132904
	C 3.625046 0.477895 -0.839461	C 3.907393 -0.065092 0.349226
	H 2.649865 1.745678 0.618296	H 2.697049 1.651882 0.988646
	H 2.045382 1.927840 -1.035525	H 2.928589 1.538859 -0.762008
	C 2.899494 -1.590239 0.357435	C 3.407156 -1.282641 -0.427861
	H 0.845719 -1.496101 1.000818	H 1.321603 -1.732117 -0.943587
	H 1.926334 -0.383006 1.858849	H 1.679900 -1.872173 0.772030
	C 4.069619 -0.647232 0.091127	H 4.008480 -0.309776 1.411129
	H 2.618499 -2.105347 -0.567747	H 4.869710 0.311285 -0.002865
	H 3.174033 -2.357113 1.088660	H 3.575147 -1.137271 -1.499769
	H 4.425937 1.211032 -0.978688	H 3.894488 -2.214719 -0.135078
	H 3.387039 0.064660 -1.825898	
	H 4.917235 -1.189243 -0.339237	
	H 4.415574 -0.219670 1.041338	
Energy (E_h)	-989.118222538	-949.825174841
Zero-point correction	0.233254	0.203367
Thermal corrections to:		
Energy	0.244961	0.214370
Enthalpy	0.245905	0.215314
Gibbs Free Energy	0.194923	0.165624

Cartesian coordinates and energies of optimized structures of data reported in Figure S27.

State	ground state (S0)
Compound	H_2O (gas phase)
Cartesian coordinates (Å)	H 0.759951 -0.473674 0.000000<br>O 0.000000 0.118418 0.000000<br>H -0.759951 -0.473674 0.000000
Energy (E_h)	-76.3910447721
Zero-point correction<br>Thermal corrections to:<br>Energy<br>Enthalpy<br>Gibbs Free Energy	0.021558<br><br>0.024393<br>0.025337<br>0.003253

State	ground state (S0)	
Compound	**1** (gas phase)	**1** + 2 H_2O
Cartesian coordinates (Å)	C -0.642984 1.983358 -0.001027<br>C -1.938301 1.559500 0.007528<br>C -2.314784 0.182708 0.005836<br>C -1.379350 -0.820636 -0.009106<br>C -0.000213 -0.414077 -0.009150<br>C 0.365838 0.978787 -0.006215<br>N 1.684598 1.160796 -0.000717<br>N 1.031570 -1.247414 0.000245<br>S 2.373399 -0.317114 0.005424<br>H -3.369763 -0.072346 0.007574<br>H -0.368169 3.030044 0.000405<br>H -2.736107 2.295581 0.016012<br>N -1.655958 -2.163091 -0.061526<br>H -2.570304 -2.467387 0.230413<br>H -0.892740 -2.781939 0.165613	C -0.862905 -2.509433 -0.003569<br>C 0.494520 -2.639270 -0.069309<br>C 1.388995 -1.537035 -0.120704<br>C 0.949153 -0.227801 -0.112301<br>C -0.483340 -0.048043 -0.037348<br>C -1.375701 -1.185240 0.015398<br>N -2.658146 -0.831735 0.082889<br>N -1.118413 1.119038 -0.007225<br>S -2.720987 0.793160 0.082210<br>H 2.457179 -1.719538 -0.169959<br>H -1.537280 -3.354439 0.035529<br>H 0.928421 -3.634786 -0.083088<br>N 1.773782 0.845130 -0.188403<br>H 2.770829 0.678078 -0.118815<br>H 1.423181 1.788428 -0.049007<br>H 5.316400 0.301807 -0.322573<br>O 4.580248 -0.055294 0.186845<br>H 4.827926 0.060960 1.111205<br>H 0.479497 3.769333 -0.883355<br>O 0.480527 3.494155 0.040873<br>H -0.261454 2.862608 0.099138
Energy (E_h)	-793.874208097	-946.687849569<br>Counterpoise corrected energy:<br>-946.677933025144
Zero-point correction<br>Thermal corrections to:<br>Energy<br>Enthalpy<br>Gibbs Free Energy	0.109003<br><br>0.116462<br>0.117407<br>0.076804	0.158233<br><br>0.172592<br>0.173536<br>0.115338

State	ground state (S0)	
Compound	**2** (gas phase)	**2** + 1 H_2O
Cartesian coordinates (Å)	C 0.578399 2.194132 0.006917<br>C -0.778087 2.323809 0.000627<br>C -1.680556 1.217205 -0.012494<br>C 1.100678 0.868926 0.001835<br>C 0.206511 -0.259794 -0.014685<br>C -1.223518 -0.080046 -0.026581<br>N 2.380718 0.505888 0.014459<br>N 0.815091 -1.436448 -0.011772<br>S 2.419869 -1.125229 0.008039<br>H -2.745284 1.415945 -0.014752<br>H 1.249117 3.043212 0.018044<br>H -1.212182 3.318953 0.007372<br>N -2.000564 -1.197143 -0.059368<br>C -3.434774 -1.155493 0.046054<br>H -3.768690 -0.679382 0.977844<br>H -3.824032 -2.173477 0.021503<br>H -3.877577 -0.604528 -0.791872<br>H -1.517887 -2.075575 0.039972	C 1.441051 2.228229 0.017231<br>C 0.197130 2.784759 0.020591<br>C -1.006546 2.024354 0.014391<br>C 1.505846 0.808094 0.005615<br>C 0.298262 0.014193 0.000307<br>C -1.005408 0.643447 0.006714<br>N 2.611013 0.066826 -0.003159<br>N 0.530688 -1.293309 -0.011970<br>S 2.157006 -1.494276 -0.018974<br>H -1.949610 2.555914 0.016906<br>H 2.351077 2.813182 0.021133<br>H 0.102448 3.866363 0.027646<br>N -2.121761 -0.125973 0.008199<br>C -3.442505 0.442853 -0.043812<br>H -3.651316 1.070387 0.832088<br>H -4.170834 -0.368050 -0.063989<br>H -3.589887 1.058961 -0.940634<br>H -2.038528 -1.139215 -0.040636<br>H -0.827279 -2.697602 -0.111791<br>O -1.746296 -3.019097 -0.047884<br>H -1.834360 -3.307139 0.868216
Energy (E_h)	-833.159737850	-909.570497793<br>Counterpoise corrected energy:<br>-909.564121585136
Zero-point correction<br>Thermal corrections to:<br>Energy<br>Enthalpy<br>Gibbs Free Energy	0.137365<br><br>0.146526<br>0.147470<br>0.102456	0.163037<br><br>0.174855<br>0.175799<br>0.124898

State	ground state (S0)	
Compound	**3** (gas phase)	**3** + 1 H_2O
Cartesian coordinates (Å)	C -1.125746 2.213216 0.134683<br>C 0.185089 2.570599 0.121247<br>C 1.247462 1.620879 0.044741<br>C -1.413186 0.822284 0.052450<br>C -0.350763 -0.156765 -0.035351<br>C 1.037417 0.262388 -0.034439<br>N -2.627807 0.281837 0.019775<br>N -0.802153 -1.401194 -0.143971<br>S -2.430009 -1.327998 -0.120021<br>H 2.258576 2.004454 0.062541<br>H -1.934621 2.929864 0.193959<br>H 0.455123 3.620251 0.181062<br>N 2.065902 -0.656746 -0.190180<br>C 3.398310 -0.131940 -0.395841<br>H 3.395158 0.606946 -1.200011<br>H 4.053974 -0.953475 -0.691133<br>H 3.825316 0.335879 0.505904<br>C 2.053753 -1.897688 0.574917<br>H 1.052061 -2.315379 0.607275<br>H 2.418264 -1.745091 1.602264<br>H 2.710687 -2.620605 0.084679	C -1.881067 2.054135 -0.203328<br>C -0.718913 2.755510 -0.255326<br>C 0.563127 2.136252 -0.123699<br>C -1.780459 0.644002 -0.035032<br>C -0.492899 0.002279 0.085973<br>C 0.721208 0.786048 0.057014<br>N -2.797611 -0.212665 -0.007415<br>N -0.578039 -1.321493 0.183210<br>S -2.164834 -1.702128 0.144867<br>H 1.435015 2.776616 -0.158899<br>H -2.854650 2.517647 -0.298396<br>H -0.746615 3.831858 -0.390127<br>N 1.964668 0.142826 0.130190<br>C 3.129516 0.939616 -0.211712<br>H 2.982910 1.426476 -1.177676<br>H 3.991549 0.274073 -0.288821<br>H 3.359190 1.709058 0.541701<br>C 2.208487 -0.695587 1.308973<br>H 1.315407 -1.261203 1.560594<br>H 2.506212 -0.085137 2.173221<br>H 2.998861 -1.410051 1.074817<br>H 2.012711 -1.565057 -1.096336<br>O 1.986101 -2.531082 -1.171117<br>H 1.130833 -2.725771 -0.768095
Energy (E_h)	-872.436069595	-948.840141554<br>Counterpoise corrected energy:<br>-948.834199676822
Zero-point correction<br>Thermal corrections to:<br>Energy<br>Enthalpy<br>Gibbs Free Energy	0.166332<br><br>0.176439<br>0.177383<br>0.130498	0.191336<br><br>0.204538<br>0.205482<br>0.151633

State	ground state (S0)	
Compound	**6** (gas phase)	**6** + 1 H_2O
Cartesian coordinates (Å)	C -2.701259 1.813464 0.131795	C 2.859058 1.868180 0.042018
	C -1.605803 2.615274 0.191655	C 1.790409 2.703174 0.118487
	C -0.270817 2.107590 0.162816	C 0.439874 2.232974 0.082203
	C -2.470838 0.413292 0.019808	C 2.588362 0.473607 -0.054507
	C -1.126447 -0.116826 -0.021951	C 1.230689 -0.015640 -0.078648
	C 0.017713 0.767682 0.058302	C 0.118920 0.904849 -0.021770
	N -3.407797 -0.524825 -0.083884	N 3.496671 -0.497156 -0.098121
	N -1.091470 -1.436413 -0.168839	N 1.154619 -1.342571 -0.123908
	S -2.636581 -1.951909 -0.230627	S 2.686260 -1.905717 -0.152634
	H 0.537034 2.823198 0.242168	H -0.349489 2.971795 0.129967
	H -3.714259 2.194121 0.155825	H 3.883205 2.218190 0.064951
	H -1.732415 3.689920 0.277111	H 1.949415 3.773498 0.199451
	N 1.307468 0.253399 -0.047481	N -1.193794 0.399638 -0.010384
	C 2.388613 1.198282 -0.290872	C -2.241085 1.332948 0.408097
	C 1.689690 -0.826425 0.871983	C -1.581354 -0.355566 -1.221868
	C 3.622821 0.483942 -0.836994	C -3.545790 0.591277 0.683586
	H 2.655095 1.745253 0.631876	H -2.410510 2.103315 -0.364676
	H 2.044239 1.936478 -1.018610	H -1.901005 1.843333 1.312628
	C 2.896195 -1.592485 0.344530	C -2.871314 -1.133850 -0.992235
	H 0.844558 -1.499180 0.992920	H -0.770850 -1.036704 -1.475609
	H 1.930281 -0.397577 1.860761	H -1.707117 0.350945 -2.059511
	C 4.067702 -0.649870 0.082793	C -3.995927 -0.205836 -0.538710
	H 2.609993 -2.098530 -0.583994	H -2.683421 -1.888728 -0.222743
	H 3.171586 -2.366547 1.067921	H -3.140325 -1.651795 -1.918325
	H 4.425176 1.216261 -0.973081	H -4.308111 1.318963 0.979382
	H 3.380344 0.079157 -1.825777	H -3.396282 -0.088172 1.529394
	H 4.913312 -1.189664 -0.354402	H -4.899312 -0.778630 -0.309458
	H 4.417523 -0.230997 1.035552	H -4.255053 0.486062 -1.351286
		H -1.207738 -1.109476 1.360901
		O -1.272455 -1.981780 1.785285
		H -0.557451 -2.462966 1.351763
Energy (E_h)	-989.116430733	-1065.52311174<br>Counterpoise corrected energy:<br>-1065.515618082224
Zero-point correction	0.233357	0.258575
Thermal corrections to:		
Energy	0.245060	0.273314
Enthalpy	0.246004	0.274258
Gibbs Free Energy	0.195019	0.216616

State	ground state (S0)	
Compound	**7** (gas phase)	**7** + 1 H_2O
Cartesian coordinates (Å)	C -2.673249 1.834499 0.128237<br>C -1.568211 2.623679 0.176186<br>C -0.239747 2.099577 0.139913<br>C -2.460351 0.431197 0.022956<br>C -1.123170 -0.115810 -0.025581<br>C 0.030504 0.756030 0.040025<br>N -3.409225 -0.496425 -0.066640<br>N -1.105888 -1.437211 -0.161874<br>S -2.657793 -1.933543 -0.208210<br>H 0.577037 2.806137 0.207679<br>H -3.681475 2.227217 0.157543<br>H -1.681177 3.700133 0.256509<br>N 1.315109 0.229436 -0.073976<br>C 2.412573 1.165666 -0.263383<br>C 1.695482 -0.875743 0.813224<br>C 3.649278 0.416087 -0.739082<br>H 2.649646 1.707080 0.667885<br>H 2.130657 1.900845 -1.021635<br>C 2.954783 -1.542639 0.289822<br>H 0.887247 -1.602873 0.853177<br>H 1.885308 -0.495057 1.829663<br>O 4.012248 -0.612973 0.154655<br>H 2.741004 -2.017377 -0.679647<br>H 3.295142 -2.309553 0.989249<br>H 4.500322 1.098318 -0.803188<br>H 3.455661 -0.000260 -1.739592	C 2.829934 1.892160 0.050112<br>C 1.752708 2.714469 0.140792<br>C 0.407549 2.229049 0.109762<br>C 2.574082 0.495683 -0.056161<br>C 1.222254 -0.009621 -0.075026<br>C 0.101505 0.898284 -0.003080<br>N 3.493292 -0.464048 -0.114234<br>N 1.162612 -1.337343 -0.131192<br>S 2.700890 -1.881599 -0.175264<br>H -0.389346 2.958787 0.169084<br>H 3.850296 2.253208 0.068624<br>H 1.899871 3.785782 0.229456<br>N -1.206818 0.382219 0.014242<br>C -2.269570 1.316230 0.384290<br>C -1.595230 -0.407356 -1.173056<br>C -3.574494 0.555675 0.575676<br>H -2.414102 2.084537 -0.392274<br>H -1.994158 1.816530 1.316780<br>C -2.929277 -1.091012 -0.923055<br>H -0.828945 -1.156101 -1.369431<br>H -1.676376 0.256813 -2.046839<br>O -3.931493 -0.144468 -0.595615<br>H -2.811350 -1.821961 -0.112344<br>H -3.261203 -1.602896 -1.829093<br>H -4.384411 1.257114 0.789658<br>H -3.477246 -0.138809 1.423090<br>H -1.188677 -1.150845 1.402241<br>O -1.231297 -2.053682 1.757354<br>H -0.496630 -2.478531 1.298127
Energy (E_h)	-1025.00767608	-1101.41337691<br>Counterpoise corrected energy:<br>-1101.405952529418
Zero-point correction<br>Thermal corrections to:<br>Energy<br>Enthalpy<br>Gibbs Free Energy	0.209304<br><br>0.220719<br>0.221663<br>0.171186	0.234429<br><br>0.248923<br>0.249867<br>0.192586

State	ground state (S0)	
Compound	**8** (gas phase)	**8** + 1 H_2O
Cartesian coordinates (Å)	C -3.070395 1.718259 0.134596	C 3.158371 1.822933 -0.018852
	C -2.022053 2.578456 0.220006	C 2.110050 2.683435 0.057140
	C -0.660577 2.143654 0.205899	C 0.748555 2.242173 0.051244
	C -2.763154 0.333465 0.012396	C 2.856711 0.432661 -0.084640
	C -1.392228 -0.121003 -0.015803	C 1.489014 -0.025258 -0.077811
	C -0.301087 0.823649 0.088641	C 0.400458 0.920059 -0.019828
	N -3.644779 -0.654427 -0.112894	N 3.741121 -0.560109 -0.125091
	N -1.279977 -1.435277 -0.172891	N 1.378946 -1.350326 -0.098848
	S -2.793612 -2.034496 -0.261256	S 2.896689 -1.949464 -0.142257
	H 0.107192 2.900187 0.306840	H -0.024673 2.998563 0.095791
	H -4.102817 2.043313 0.147555	H 4.190012 2.151152 -0.018401
	H -2.208500 3.643325 0.315695	H 2.293922 3.751179 0.114304
	N 1.016144 0.372204 -0.009676	N -0.923193 0.434384 0.023398
	C 2.043943 1.362929 -0.295231	C -1.930303 1.379294 0.512256
	C 1.439638 -0.666050 0.936854	C -1.341755 -0.281604 -1.201125
	C 3.260092 0.732486 -0.964056	C -3.218269 0.671274 0.913060
	H 2.359489 1.898168 0.615365	H -2.155948 2.149690 -0.241562
	H 1.623009 2.096466 -0.987394	H -1.515323 1.876529 1.393541
	C 2.578289 -1.508874 0.385949	C -2.574144 -1.142358 -0.968726
	H 0.591039 -1.318362 1.132977	H -0.517583 -0.925624 -1.507906
	H 1.752848 -0.203470 1.885808	H -1.535048 0.444363 -2.004695
	S 4.073168 -0.523211 0.067567	S -4.023317 -0.150870 -0.493981
	H 2.259731 -2.005881 -0.534059	H -2.368131 -1.874169 -0.184362
	H 2.859312 -2.274446 1.112307	H -2.835867 -1.665259 -1.890863
	H 4.008559 1.501409 -1.167781	H -3.929954 1.402954 1.301012
	H 2.962726 0.283278 -1.915555	H -3.017464 -0.065760 1.694789
		H -0.913086 -1.062878 1.361290
		O -1.100733 -1.873165 1.866229
		H -0.409179 -2.473383 1.564528
Energy (E_h)	-1348.00288398	-1424.40949928<br>Counterpoise corrected energy:<br>-1424.401866181308
Zero-point correction	0.205642	0.230947
<u>Thermal corrections to:</u>		
Energy	0.217678	0.245997
Enthalpy	0.218623	0.246941
Gibbs Free Energy	0.166357	0.188196

State	ground state (S0)	
Compound	**9** (gas phase)	**9** + 1 H_2O
Cartesian coordinates (Å)	C -3.182111 1.667838 0.089497<br>C -2.147149 2.546247 0.148400<br>C -0.779514 2.134667 0.138498<br>C -2.851373 0.286490 -0.000317<br>C -1.472354 -0.147917 -0.022858<br>C -0.395246 0.817371 0.054267<br>N -3.718679 -0.716969 -0.097418<br>N -1.343739 -1.464008 -0.148663<br>S -2.848177 -2.087929 -0.215585<br>H -0.026051 2.907475 0.215049<br>H -4.219825 1.975487 0.099367<br>H -2.350450 3.610175 0.218017<br>N 0.929833 0.400560 -0.033911<br>C 1.945376 1.424489 -0.218949<br>C 1.386264 -0.660900 0.869297<br>C 3.247686 0.796861 -0.694967<br>H 2.131268 1.982582 0.713683<br>H 1.599047 2.132552 -0.975480<br>C 2.691905 -1.254178 0.370316<br>H 0.629101 -1.439577 0.917062<br>H 1.542459 -0.250973 1.879659<br>N 3.705477 -0.220858 0.233589<br>H 2.509131 -1.774418 -0.588527<br>H 3.041783 -1.999404 1.091829<br>H 4.010399 1.578756 -0.769989<br>H 3.094078 0.381679 -1.709208<br>C 4.987432 -0.768932 -0.158575<br>H 5.733779 0.029041 -0.204647<br>H 4.960881 -1.269564 -1.143647<br>H 5.319489 -1.500229 0.583368	C 3.263082 1.768401 -0.005335<br>C 2.235635 2.651482 0.091316<br>C 0.864913 2.243407 0.086148<br>C 2.926496 0.387810 -0.091347<br>C 1.547768 -0.039979 -0.083892<br>C 0.480754 0.930896 -0.005241<br>N 3.789142 -0.622812 -0.152440<br>N 1.412873 -1.362709 -0.124442<br>S 2.916964 -1.993871 -0.185417<br>H 0.112084 3.018368 0.146811<br>H 4.302485 2.070845 -0.006120<br>H 2.444766 3.713664 0.164755<br>N -0.853814 0.492093 0.039147<br>C -1.852816 1.488174 0.419866<br>C -1.307944 -0.280971 -1.133938<br>C -3.190994 0.813819 0.681968<br>H -1.981883 2.244289 -0.370862<br>H -1.514828 1.993387 1.328148<br>C -2.656084 -0.923160 -0.855304<br>H -0.572612 -1.053381 -1.352289<br>H -1.390675 0.388047 -2.003523<br>N -3.638556 0.091592 -0.496792<br>H -2.532231 -1.675338 -0.058249<br>H -2.993720 -1.434883 -1.762338<br>H -3.929003 1.584431 0.927173<br>H -3.098119 0.149372 1.560620<br>C -4.955546 -0.482744 -0.305034<br>H -5.674861 0.308957 -0.077224<br>H -4.989247 -1.223838 0.512895<br>H -5.278933 -0.977979 -1.224749<br>H -0.907514 -1.022533 1.468871<br>O -0.940150 -1.918963 1.840356<br>H -0.245034 -2.359733 1.336347
Energy (E_h)	-1044.43090582	-1120.83681257<br>Counterpoise corrected energy:<br>-1120.829409771097
Zero-point correction<br>Thermal corrections to:<br>Energy<br>Enthalpy<br>Gibbs Free Energy	0.249855<br><br>0.262848<br>0.263792<br>0.209929	0.275157<br><br>0.291206<br>0.292150<br>0.231671

State	ground state (S0)	
Compound	**10** (gas phase)	**10** + 1 H_2O
Cartesian coordinates (Å)	C 2.296948 1.929502 0.081845	C -2.445890 2.003437 0.083905
	C 1.141539 2.650152 0.087624	C -1.318839 2.742969 0.265687
	C -0.150797 2.057487 0.051167	C -0.007754 2.181132 0.248588
	C 2.168417 0.515440 0.028871	C -2.274524 0.602934 -0.093841
	C 0.864174 -0.114604 -0.011014	C -0.955440 0.012628 -0.099365
	C -0.342452 0.688255 0.005305	C 0.221869 0.838807 0.045637
	N 3.169096 -0.360904 -0.002503	N -3.247340 -0.295384 -0.225461
	N 0.925511 -1.439326 -0.079066	N -0.975857 -1.312728 -0.208678
	S 2.503965 -1.846225 -0.082614	S -2.542685 -1.757506 -0.324552
	H -1.008574 2.716199 0.070823	H 0.829129 2.852071 0.390129
	H 3.278430 2.384128 0.109693	H -3.440285 2.430388 0.090513
	H 1.191292 3.734126 0.125851	H -1.402117 3.814084 0.419921
	N -1.584309 0.123514 -0.048888	N 1.482610 0.269955 0.043851
	C -2.780065 0.948789 -0.151736	C 2.648540 1.120505 0.278847
	C -1.906181 -1.295986 0.132574	C 1.838824 -0.846084 -0.872140
	C -3.905872 -0.063285 -0.352069	C 3.820568 0.183842 0.019024
	H -2.693550 1.654662 -0.985290	H 2.626650 1.528572 1.293768
	H -2.931234 1.540189 0.764221	H 2.674374 1.968737 -0.423627
	C -3.407153 -1.282810 0.423110	C 3.321753 -0.619220 -1.182671
	H -1.322349 -1.732674 0.943400	H 1.215267 -0.828796 -1.769070
	H -1.674437 -1.868999 -0.772325	H 1.681190 -1.796049 -0.361852
	H -4.003927 -0.305642 -1.414871	H 3.973105 -0.473289 0.880646
	H -4.869809 0.311642 -0.002386	H 4.750975 0.722275 -0.171688
	H -3.578843 -1.140392 1.494942	H 3.434809 -0.026496 -2.096152
	H -3.893884 -2.214269 0.126907	H 3.856446 -1.559978 -1.326794
		H 1.300744 -1.196428 1.744490
		O 1.116978 -2.121821 1.959311
		H 0.336320 -2.299025 1.418049
Energy (E_h)	-949.823240186	-1026.22534753<br>Counterpoise corrected energy:<br>-1026.218840696475
Zero-point correction	0.203479	0.228574
<u>Thermal corrections to:</u>		
Energy	0.214475	0.242668
Enthalpy	0.215419	0.243612
Gibbs Free Energy	0.165736	0.186806

State	ground state (S0)	
Compound	**11** (gas phase)	**11** + 1 H_2O
Cartesian coordinates (Å)	C 2.824285 2.046062 -0.194412<br>C 1.562213 2.536771 -0.347664<br>C 0.388896 1.723586 -0.357700<br>C 2.951721 0.636842 -0.035513<br>C 1.779841 -0.200488 -0.049429<br>C 0.459768 0.355397 -0.223006<br>N 4.073752 -0.057045 0.137954<br>N 2.032609 -1.490676 0.113671<br>S 3.653542 -1.628110 0.271530<br>H -0.571885 2.208419 -0.478320<br>H 3.703225 2.677119 -0.186038<br>H 1.424195 3.607148 -0.466758<br>N -0.583407 -0.521055 -0.260947<br>C -1.980057 -0.140762 -0.275727<br>H -0.352675 -1.476224 -0.028667<br>C -2.500618 0.352559 1.079303<br>C -2.826216 -1.325577 -0.773105<br>H -2.080840 0.680569 -0.996745<br>C -3.920234 0.933028 0.939475<br>H -1.817386 1.097219 1.496425<br>H -2.497201 -0.495020 1.774873<br>C -4.732291 0.226627 -0.164542<br>H -3.865006 2.004802 0.722101<br>H -4.439201 0.842943 1.898858<br>C -4.293776 -1.229532 -0.316681<br>H -4.592459 0.741598 -1.122501<br>H -5.801819 0.285414 0.057189<br>H -4.938816 -1.758227 -1.024184<br>H -4.417526 -1.736938 0.646887<br>H -2.389733 -2.257826 -0.394269<br>H -2.761434 -1.376876 -1.864079	C -2.759374 -2.329769 -0.195297<br>C -1.480231 -2.781897 -0.324133<br>C -0.337508 -1.932938 -0.318052<br>C -2.927093 -0.926129 -0.048013<br>C -1.782784 -0.043588 -0.045119<br>C -0.437725 -0.560848 -0.191903<br>N -4.080035 -0.277730 0.098285<br>N -2.108335 1.235583 0.102415<br>S -3.740278 1.306915 0.229980<br>H 0.638043 -2.391096 -0.416796<br>H -3.621472 -2.983251 -0.197297<br>H -1.305760 -3.847873 -0.434584<br>N 0.602697 0.311492 -0.215479<br>C 1.995329 -0.087062 -0.270785<br>H 0.417240 1.294640 -0.032629<br>C 2.535149 -0.605478 1.067632<br>C 2.842161 1.103484 -0.758265<br>H 2.079446 -0.897136 -1.007533<br>C 3.947406 -1.194023 0.895125<br>H 1.854695 -1.351215 1.487509<br>H 2.549097 0.232575 1.773686<br>C 4.749702 -0.468618 -0.203780<br>H 3.881173 -2.260488 0.654741<br>H 4.479914 -1.129322 1.849215<br>C 4.314269 0.991567 -0.321088<br>H 4.595988 -0.963730 -1.170205<br>H 5.821634 -0.536602 0.003966<br>H 4.953590 1.532162 -1.025077<br>H 4.450690 1.479728 0.650639<br>H 2.411415 2.028244 -0.359562<br>H 2.769570 1.166867 -1.848851<br>H -0.880027 2.759264 0.252092<br>O -0.012467 3.195552 0.159464<br>H -0.007146 3.518563 -0.749199
Energy (E_h)	-1028.41364085	-1104.82395723<br>Counterpoise corrected energy:<br>-1104.816866626463
Zero-point correction<br>Thermal corrections to:<br>Energy<br>Enthalpy<br>Gibbs Free Energy	0.261940<br><br>0.275373<br>0.276318<br>0.220517	0.287336<br><br>0.303662<br>0.304606<br>0.242537

State	ground state (S0)	
Compound	**12** (gas phase)	**12** + 1 H_2O
Cartesian coordinates (Å)	C 2.617377 2.047590 -0.230152<br>C 1.347398 2.523924 -0.354419<br>C 0.181431 1.699530 -0.305175<br>C 2.763653 0.642734 -0.052379<br>C 1.599452 -0.201393 0.004624<br>C 0.270258 0.342352 -0.115232<br>N 3.898620 -0.041094 0.069766<br>N 1.871261 -1.489059 0.162537<br>S 3.498540 -1.612831 0.235866<br>H -0.784244 2.165940 -0.444185<br>H 3.490471 2.685803 -0.268171<br>H 1.194278 3.588054 -0.504336<br>N -0.754106 -0.572450 -0.061209<br>C -2.137093 -0.358042 -0.006025<br>H -0.447244 -1.533994 -0.104642<br>C -2.705816 0.739332 0.645736<br>C -2.977872 -1.317387 -0.578668<br>C -4.087221 0.878029 0.695147<br>C -4.920687 -0.072781 0.119694<br>C -4.355360 -1.177077 -0.509069<br>H -2.539451 -2.171483 -1.086305<br>H -5.998122 0.041489 0.165124<br>H -4.990888 -1.932874 -0.959150<br>H -2.071486 1.463982 1.140044<br>H -4.513490 1.735752 1.205620	C 2.569496 2.322118 -0.263184<br>C 1.287532 2.760773 -0.399558<br>C 0.151044 1.903509 -0.335414<br>C 2.748824 0.926780 -0.060571<br>C 1.610783 0.040340 0.009075<br>C 0.262469 0.548186 -0.119184<br>N 3.910254 0.291712 0.076454<br>N 1.950144 -1.231627 0.193968<br>S 3.584746 -1.287996 0.270974<br>H -0.827059 2.339607 -0.484015<br>H 3.428043 2.978842 -0.310149<br>H 1.103450 3.816625 -0.571566<br>N -0.767324 -0.353797 -0.039597<br>C -2.145402 -0.099963 0.011033<br>H -0.528956 -1.340299 -0.142816<br>C -2.699671 0.998296 0.674532<br>C -2.999580 -1.043436 -0.571328<br>C -4.079181 1.157761 0.722258<br>C -4.925233 0.227042 0.132682<br>C -4.374589 -0.880141 -0.504886<br>H -2.566590 -1.904305 -1.071172<br>H -6.000978 0.358434 0.174913<br>H -5.020963 -1.621781 -0.963375<br>H -2.056969 1.709309 1.178276<br>H -4.493828 2.016484 1.240826<br>H -0.370329 -3.511230 0.694652<br>O -0.110589 -3.192955 -0.177994<br>H 0.772481 -2.801722 -0.045729
Energy (E_h)	-1024.79437809	-1101.20495978<br>Counterpoise corrected energy:<br>-1101.198215871613
Zero-point correction<br><u>Thermal corrections to:</u><br>Energy<br>Enthalpy<br>Gibbs Free Energy	0.191388<br><br>0.203224<br>0.204168<br>0.151778	0.216691<br><br>0.231412<br>0.232356<br>0.173662

State	ground state (S0)	
Compound	**13** (gas phase)	**13** + 1 H_2O
Cartesian coordinates (Å)	C 2.710778 -2.455587 1.046273	C -3.041837 -1.865656 -1.533141
	C 1.461753 -2.728155 1.512444	C -1.872003 -2.116344 -2.182728
	C 0.331724 -1.912314 1.203243	C -0.635909 -1.513637 -1.792091
	C 2.860297 -1.319519 0.201463	C -2.996474 -1.000023 -0.403257
	C 1.722434 -0.497935 -0.137026	C -1.750221 -0.409959 0.011554
	C 0.420642 -0.807404 0.398151	C -0.545346 -0.658845 -0.728656
	N 3.988145 -0.913449 -0.376865	N -4.014951 -0.684254 0.394599
	N 2.015635 0.494864 -0.968058	N -1.851762 0.313871 1.122133
	S 3.611138 0.392623 -1.274020	S -3.418578 0.262188 1.573741
	H -0.633668 -2.180471 1.616582	H 0.261717 -1.737064 -2.358443
	H 3.571790 -3.067996 1.282657	H -3.982831 -2.310140 -1.832385
	H 1.304324 -3.586892 2.156830	H -1.865590 -2.780015 -3.040867
	N -0.690066 -0.020645 0.055055	N 0.652942 -0.034645 -0.325440
	C -0.622552 1.393256 0.192687	C 0.718585 1.392286 -0.423527
	C -1.954373 -0.641671 -0.137087	C 1.820514 -0.797823 -0.110873
	C -1.268512 2.212923 -0.733810	C 1.189055 2.145613 0.652275
	C 0.075553 1.972642 1.250867	C 0.305559 2.028417 -1.591801
	C -1.215395 3.591718 -0.597620	C 1.261364 3.526861 0.540857
	C -0.506291 4.171549 0.450158	C 0.849320 4.166746 -0.624716
	C 0.141218 3.355330 1.368742	C 0.363321 3.414339 -1.686232
	H 0.565266 1.336553 1.980354	H -0.059728 1.435233 -2.423373
	H -0.459732 5.250861 0.548189	H 0.899978 5.247902 -0.700707
	H 0.692475 3.794319 2.194143	H 0.036581 3.903717 -2.598081
	H -1.804754 1.760092 -1.560141	H 1.448838 1.641710 1.577518
	H -1.720498 4.218997 -1.325091	H 1.624013 4.109762 1.381303
	C -3.095521 -0.157720 0.503810	C 3.085827 -0.284209 -0.410287
	C -2.064409 -1.745530 -0.986351	C 1.726604 -2.084464 0.436247
	C -4.326190 -0.766222 0.290422	C 4.223395 -1.039582 -0.160683
	C -4.432697 -1.870260 -0.546286	C 4.128838 -2.317984 0.376331
	C -3.293739 -2.357963 -1.179560	C 2.871835 -2.833961 0.668861
	H -3.364171 -3.214223 -1.842641	H 2.775989 -3.828180 1.093456
	H -1.179553 -2.115775 -1.492048	H 0.751700 -2.497740 0.671076
	H -3.013981 0.698109 1.163987	H 3.174078 0.708051 -0.835189
	H -5.205721 -0.377843 0.793834	H 5.196214 -0.621629 -0.399286
	H -5.394305 -2.345928 -0.706375	H 5.021718 -2.903130 0.566450
		H -0.170756 0.222568 2.464677
		O 0.598300 0.023216 3.020525
		H 1.080737 -0.647234 2.521972
Energy (E_h)	-1255.70209234	-1332.10651123<br>Counterpoise corrected energy:<br>-1332.099742501843
Zero-point correction	0.272246	0.297109
<u>Thermal corrections to:</u>		
Energy	0.288716	0.316777
Enthalpy	0.289660	0.317721
Gibbs Free Energy	0.225963	0.246864

IX. References and Notes

[1] J. Neumann, S. Elangovan, A. Spannenberg, K. Junge, M. Beller, *Chem. Eur. J.* **2017**, *23*, 5410-5413.

[2] N. M. Slavachevskaya, I. A. Belen'kaya, N. S. Tsepova, E. I. Levocheskaya, I. I. Krasil'nikov, *Pharm. Chem. J.* **1976**, *10*, 327-331.

[3] V. Kumar, M. Kumar, S. Sharma, N. Kumar, *RSC Advances* **2014**, *4*, 11826-11830.

[4] C. Reddy, N. Bisht, R. Parella, S. A. Babu, *J. Org. Chem.* **2016**, *81*, 12143-12168.

[5] T. Suzuki, T. Tsuji, T. Okubo, A. Okada, Y. Obana, T. Fukushima, T. Miyashi, Y. Yamashita, *J. Org. Chem.* **2001**, *66*, 8954-8960.

[6] G. R. Fulmer, A. J. M. Miller, N. H. Sherden, H. E. Gottlieb, A. Nudelman, B. M. Stoltz, J. E. Bercaw, K. I. Goldberg, *Organometallics* **2010**, *29*, 2176-2179.

[7] R. Sjöback, J. Nygren, M. Kubista, *Spectrochim. Acta, Part A* **1995**, *51*, L7-L21.

[8] A. M. Brouwer, *Pure Appl. Chem.* **2011**, *83*, 2213-2228.

[9] I. Z. Kozma, P. Krok, E. Riedle, *J. Opt. Soc. Am. B* **2005**, *22*, 1479-1485.

[10] K. Moutzouris, M. Papamichael, S. C. Betsis, I. Stavrakas, G. Hloupis, D. Triantis, *Appl. Phys. B* **2014**, *116*, 617-622.

[11] S. Kedenburg, M. Vieweg, T. Gissibl, H. Giessen, *Opt. Mater. Express* **2012**, *2*, 1588-1611.

[12] Molinspiration Cheminformatics free web services, https://molinspiration.com/, last accessed March 2021.

[13] Gaussian 09, Revision D.01, M. J. Frisch, G. W. Trucks, H. B. Schlegel, G. E. Scuseria, M. A. Robb, J. R. Cheeseman, G. Scalmani, V. Barone, B. Mennucci, G. A. Petersson, H. Nakatsuji, M. Caricato, X. Li, H. P. Hratchian, A. F. Izmaylov, J. Bloino, G. Zheng, J. L. Sonnenberg, M. Hada, M. Ehara, K. Toyota, R. Fukuda, J. Hasegawa, M. Ishida, T. Nakajima, Y. Honda, O. Kitao, H. Nakai, T. Vreven, J. A. Montgomery, Jr., J. E. Peralta, F. Ogliaro, M. Bearpark, J. J. Heyd, E. Brothers, K. N. Kudin, V. N. Staroverov, T. Keith, R. Kobayashi, J. Normand, K. Raghavachari, A. Rendell, J. C. Burant, S. S. Iyengar, J. Tomasi, M. Cossi, N. Rega, J. M. Millam, M. Klene, J. E. Knox, J. B. Cross, V. Bakken, C. Adamo, J. Jaramillo, R. Gomperts, R. E. Stratmann, O. Yazyev, A. J. Austin, R. Cammi, C. Pomelli, J. W. Ochterski, R. L. Martin, K. Morokuma, V. G. Zakrzewski, G. A. Voth, P. Salvador, J. J. Dannenberg, S. Dapprich, A. D. Daniels, O. Farkas, J. B. Foresman, J. V. Ortiz, J. Cioslowski, and D. J. Fox, Gaussian Inc., Wallingford CT, **2013**.

[14] M. D. Hanwell, D. E. Curtis, D. C. Lonie, T. Vandermeersch, E. Zurek, G. R. Hutchison, *J. Cheminform.* **2012**, *4*, 17.

[15] Avogadro: an open-source molecular builder and visualization tool. Version 1.2.0. http://avogadro.cc/.

[16] We noticed that permeabilization with Triton X was actually not necessary. Incubation with the compounds (1 h, rt) directly after fixation resulted in good staining.

[17] Used fluorescent microscope channels: blue, ex. 325-375 nm, em. 435-485 nm; green, ex. 460-500 nm, em. 512-542 nm; red, ex. 590-650 nm, em. 662-738 nm.

[18] J. Schindelin, I. Arganda-Carreras, E. Frise, V. Kaynig, M. Longair, T. Pietzsch, S. Preibisch, C. Rueden, S. Saalfeld, B. Schmid, J.-Y. Tinevez, D. J. White, V. Hartenstein, K. Eliceiri, P. Tomancak, A. Cardona, *Nat. Methods* **2012**, *9*, 676-682.

[19] C. A. Schneider, W. S. Rasband, K. W. Eliceiri, *Nat. Methods* **2012**, *9*, 671-675.

[20] Used antibodies and associated organelles: ADFP (lipid droplets); AIF (mitochondria); EEA1 (endosomes); LAMP1 (lysosomes); PDI (endoplasmic reticulum); RCAS1 (Golgi).

[21] W. Chen, L. Wang, S. Yan, D. J. Loury, Z. J. Jia, L. L. Frye, J. R. Greenwood, M. Y. Shelley, G. B. Atallah, R. Zanaletti, M. P. Catalani, L. F. Raveglia, *International Patent, WO 2016/004272 A1*, **2016**.

[22] A.-C. C. Carlsson, K. Mehmeti, M. Uhrbom, A. Karim, M. Bedin, R. Puttreddy, R. Kleinmaier, A. A. Neverov, B. Nekoueishahraki, J. Gräfenstein, K. Rissanen, M. Erdélyi, *J. Am. Chem. Soc.* **2016**, *138*, 9853-9863.

[23] B. Latli, M. Hrapchak, M. Chevliakov, G. Li, S. Campbell, C. A. Busacca, C. H. Senanayake, *J. Labelled Compd. Radiopharm.* **2015**, *58*, 250-260.
[24] N. Miyake, S. Okada, J. Irisawa, T. Konishi, K. Matsuura, *U.S. Patent Application, US 2018/0346729 A1*, **2018**.
[25] Y. Yu, Z. Xu, Z. Zhao, H. Zhang, D. Ma, J. W. Y. Lam, A. Qin, B. Z. Tang, *ACS Omega* **2018**, *3*, 16347-16356.
[26] Y. Xu, X. Yang, Y. Chen, H. Chen, H. Sun, W. Li, Q. Xie, L. Yu, L. Shao, *Bioorg. Med. Chem. Lett.* **2018**, *28*, 2148-2152.
[27] H. Appelqvist, K. Stranius, K. Börjesson, K. P. R. Nilsson, C. Dyrager, *Bioconjugate Chem.* **2017**, *28*, 1363-1370.
